# Crop diversification and parasitic weed abundance: a global meta-analysis

**DOI:** 10.1038/s41598-022-24047-2

**Published:** 2022-11-12

**Authors:** D. Scott, R. P. Freckleton

**Affiliations:** Department of Animal and Plant Sciences, University of Sheffield, Sheffield, S10 2TN UK

**Keywords:** Agroecology, Invasive species

## Abstract

Parasitic weeds cause huge annual losses to food production globally. A small number of species from the genera *Cuscuta, Orobanche, Phelipanche* and *Striga* have proliferated across many agroecological zones. Their control is compromised due to the lack of efficacy of conventional herbicides and their rapid adaptation to new resistant crop cultivars. A broad range of studies suggest consistent reductions in parasitic weed densities owing to increased spatial (intercropping) and temporal diversity (crop rotation). However, to date, no synthesis of this body of research has been published. Here we report the results of a meta-analysis using 1525 paired observations from 67 studies across 24 countries, comparing parasitic weed density and crop yields from monocrop and more diverse cropping systems. We found both spatial and temporal crop diversification had a significant effect on parasitic weed density reduction. Furthermore, our results show effects of spatial diversification are stronger in suppressing parasitic weeds than temporal effects. Furthermore, the analysis indicates intercrops which alter both microclimate and soil chemistry (e.g. *Crotalaria, Stylosanthes*, Berseem clover and *Desmodium*) are most effective in parasitic weed management. This analysis serves to underline the viability of crop diversification as a tool to enhance food security globally.

## Introduction

Weeds currently represent the most significant factor limiting agricultural production, with crop yield reductions attributable to weeds estimated at 34% globally^1,2^. Amongst the most serious weeds are a small number of parasitic plants of the genera *Cuscuta*, *Orobanche, Phelipanche* and *Striga* have proliferated, impacting food production worldwide^3–5^. Parasitic weeds disproportionately affect subsistence farming in the developed world^6^, exacerbating food insecurity and confounding poverty alleviation initiatives. Simplification of cropping systems has been recognised as a key driver of agricultural weeds in general^7^. This is also the case for parasitic weeds, which predominantly affect low-diversity agricultural systems, with large-scale monocultures providing a continuous supply of host plants, facilitating their spread^5,8^.

Agrodiversity describes diversity within varieties and species of cultivated crops, crop-management systems and techniques, as well as insect and soil biodiversity^9,10^. A key element of agrodiversity is the diversity of cultivated crop species^11^, including genetic diversity at the varietal and landrace level^12^. Techniques used to enhance crop diversity include crop rotation^13^, intercropping^14^, relay cropping^15^, cover cropping^16^ and the use of cultivar mixes of the same species^12^. Under a broad range of conditions it is possible to maintain yields whilst reducing the use of chemical fertilisers and herbicides^17,18^. Furthermore, diversification has been shown to stabilise and increase yields when compared with less diverse systems^19^. Such effects have been demonstrated on field, landscape and national scales^18,20,21^ as well as across climatic gradients^22,23^.

Diversification of crop rotations has been shown to have a significant positive effect on weed control^24,25^. For example, Weisberger et al.^7^ found an average weed density reduction of 49% in diverse crop rotations, compared with monocrops. Similarly, the role of intercrops in the suppression of weeds has been demonstrated across a wide range of crop types within both tropical and temperate biomes (e.g.^26–29^). However, in some studies this effect has been less evident, with significant variability in results between crops, years and locations (e.g.^30–32^).

Parastic weeds damage crops when they attach to the host plant, and before they become visible above ground. The unique rootlike haustorium of a parasitic weed penetrates the host plant’s vascular system, and allows the parasite to assimilate nutrients and water^33^. Host plant attachment reduces the photosynthetic requirements of parasitic weeds either completely in the case of holoparasites such as *Cuscuta* or partially in the case of hemiparasites such as *Orobanche* and *Striga*^33^.

Conventional weed management typically targets above-ground growth so that techniques such as herbicide application and mechanical weeding are frequently ineffective when applied to parasitic weeds. Methods of parasitic weed control must therefore focus on the reduction of germination and primary growth. Mechanisms to reduce parasitic weed recruitment include alteration of soil chemistry^34,35^, germination in the absence of available hosts (suicidal germination)^36,37^ and altering soil microclimate^38–41^.

Intercropping can suppress weeds, including parasitic species, through several mechanisms. The effect of niche complementarity has been observed in intercrops, particularly for cereal-legume combinations, because legumes facilitate increased input of fixed N_2_ cropping systems whilst not affecting uptake N uptake for the associated cereal crop^42^. Increased resource use efficiency by intercrops through differing nutrient requirements between crops has also been shown to assist in weed suppression. For example, Haugaard-Nielsen et al.^28^ found enhanced interception of N by when intercropped with pea, compared to barley monocrop, which resulted in reduced weed incidence. Another important mechanism is the allelopathic effects of some crops on weeds when grown in rotation (e.g.^43,44^).

Additionally, a suite of alternative tactics can also help mitigate yield losses from parasitic weeds, including the use of resistant crop varieties^45–47^ and post attachment tolerance of parasitic weeds by host crops^48^. Combinations of crops, intercrops, rotation crops and varieties thereof may therefore manage or mitigate the effects of parasitic weeds in any number of ways listed above. A huge number of field trials have been undertaken to analyse the effectiveness of these approaches to reducing parasitic weed densities and mitigating yield losses. However to date there has been no synthesis of this work and, to our knowledge, no analysis has been undertaken of the relative effects of rotation and intercropping on economically significant parasitic weeds. Such an analysis will improve our ability to manage parasitic weeds through improving our understanding of the efficacy of different components of agrodiversity.

Here we present the results of a meta-analysis of the effects of crop diversity on parasitic weeds using an extensive data set derived from laboratory, field, farm and landscape studies. This represents the first quantitative synthesis of the effects of crop diversification on parasitic weeds and associated crop yields. We address the following questions: Does crop diversity, expressed as the incorporation of additional crops within a system, affect parasitic weed density or crop yield? In terms of management factors, what are the strongest predictors of variation in parasitic weed density and crop yield? Which are the best-performing combinations of crops/intercrops and/or rotation crops in terms of weed reduction and yield increase? Finally, as an ancillary analysis, we analyse the effect of climate and altitude on weed densities.

## Meta-analysis methods

### Pilot study

A pilot study was undertaken to determine terms for the main search. This comprised the systematic search for relevant studies of electronic databases: Web of Science, Scopus and AGRICOLA using a range of Boolean search terms. The pilot study used combinations of provisional terms in conjunction with the genera: *Striga* and *Orobanche* (being among the most economically significant parasitic weed genera). The number of returns for each search combination, accompanied by an assessment of relevance based on the title of each study, indicated their relevance. This determined the final list of terms for inclusion, as some terms were too broad and returned too many unrelated results. Search combinations returning very high (e.g. > 400) numbers of records with a very large proportion of non-relevant studies indicated that the term was too broad and was omitted from the main search (e.g.: “Taxon” AND inter*, “Taxon” AND Legum*).

The choice of taxa for inclusion in the main search was determined by a review of economically significant parasitic plants using several sources^33,49,50^. The list was then subject to triage based on the nature of their parasitism, removing weeds affecting woody crops (trees and shrubs). Genera that returned no results for the search combinations were removed from the main search. In the case of genera containing high numbers of economically important species (e.g.: *Cuscuta, Striga*), the genus was included as a search term alone without going to the species level. Widely adopted synonyms at the family and genus level were also included. Appendix 1 details search combinations used for the pilot, results, list of taxa, synonyms, and full search methodology.

### Main search

Main searches were performed in February 2021. Search strings were constructed as follows: First, the name for each parasitic weed taxon was used (*Aeginetia, Alectra, Christisonia, Cuscuta, Grammica, Orobanche, Phelipanche, Scrophulariaceae, Striga*). Each taxon name was then added to the following search term combinations: cover AND crop, taxon name AND Intercrop, taxon name AND trap*, taxon name AND push AND pull, taxon name AND companion, taxon name AND conservation AND agriculture *, taxon name AND integrated weed management, taxon name AND cultural AND control, taxon name AND suicidal*, taxon name AND legume, taxon name AND no AND till, taxon name AND zero AND till.

Additional searches were performed between May 2021 and February 2022 by manually searching for citations within relevant sections of 20 review studies of control methods for all economically significant parasitic weed taxa. The full list of review studies consulted is included in Appendix 2. Experts in the field of parasitic weed agronomy were also contacted to identify possible sources of data (including primary data) and to verify the thoroughness of our literature coverage. The list of studies and subsequent data were updated periodically as additional sources became available.

### Criteria for inclusion of studies

Studies were included if they fulfilled the following relevance criteria:

**Subjects studied:** Any annual parasitic weed species, any combination of host and intercrops.

**Treatment used:** Intercropping or rotation cropping.

**Study type:** Any primary landscape, laboratory, field trials, farm trials, pot, bag and rhizotron studies with appropriate comparators.

**Data type:** Continuous data with means, information on sample sizes, available/calculable measures of variance or sufficient information to impute values.

**Response(s):** Host yield (t ha^−1^/kg ha^−1^), stover yield (t ha^−1^), weed dry weight (t ha^−1^/g pot/g plant/gm^2^), weed/weed seed density (per petri dish/pot/plant/M^2^/log_10_M^2^/density/severity score), percentage weed reduction/ratio (versus control/from original density).

**Comparator:** Appropriate controls: experimental units in which no intercrop was grown with the host crop, or monocrop/fallow/bare earth in the case of rotation studies.

### Data extraction

Weed density and yield data were standardised to weeds/m^−2^ or t ha^−1^, respectively. Where reported, the long or short rainy season was also recorded. In the case of data presented in graph form, numeric data were extracted using data extraction software ‘im2graph’^51^. Data from studies were recorded to either intercrop or rotation cropping systems, as the mechanisms of impact of these on both parasitic weed density and yield are ecologically distinct.

Coordinates for study locations were directly extracted where available, or were estimated based on central coordinates of place names and extracted using Google maps^52^. In a handful of instances where it was not possible to determine separate coordinates for locations very close together (e.g. villages), data were aggregated and mean values calculated.

Studies in which there were no reported controls for the main treatment, or where data were not presented in a useable form were rejected. However, measures of variance were not reported in 53% of intercrop and 50% of rotation studies. Rejection of this proportion of studies due to missing variance risks the loss of significant volumes of data^53^. Furthermore, such omission can result in both losses of statistical power and errors in parameter estimates^54^ as well as a risk of bias toward studies that report significant results^55^. We, therefore, imputed missing variances as this has been shown to improve the reliability of meta-analysis^53^. Imputation was undertaken using the “mice” package in R using the predictive mean matching method^56^. This method was chosen as it selects values from the complete studies in the dataset predicted to be closest to values which are missing^57^. Other methods produced imputed values which were either not realistic or were negative (e.g. Random sample, Linear regression). Imputed values were estimated by averaging across ten iterations undertaken for each missing variance.

### Climate and altitude

Climate data were obtained from the WorldClim2 dataset^58^. Climate variables recorded were mean annual rainfall, mean annual temperature and precipitation seasonality. Precipitation seasonality is defined as the coefficient of variation of mean monthly precipitation^59^. Altitudes for individual study sites were obtained from the SRTM 90m Digital Elevation Database v4.1^60,61^ and were extracted using QGIS^62^.

### Statistical methods

Analyses were undertaken using linear mixed effect models weighted by the variance of effect sizes of each measure. Linear mixed effect models were used to test the overall effects of the cropping system on weed density and yield across studies. This was done by grouping the aggregated response data by cropping system.

Linear mixed-effect models were also used to identify the effect of management factors on weed density and yield across studies. Two groups of factors were included in these models with effect size as the response (weighted by the study variance), and study ID included as a random effect. Significance tests were conducted using Satterthwaites approximation in the R-package lmerTest^63^. This approach yields lower Type I error rates than alternatives^64^.

The effect size was estimated as Hedge’s g and its variance (standardised mean difference). This was done by calculating the difference between the treatment and control (weed density, weed dry weight or host yield) divided by the pooled standard deviation using the “compute.es” package in R^65^.

Linear models were used to determine the effect of rainfall CV, mean annual temperature, mean annual rainfall, and altitude on parasitic weed density and crop yield. These analyses were undertaken using a subset of studies where initial weed density was not deemed to have been manipulated (i.e.: farm, field trial or landscape). We also used local polynomial regression (LOESS) to visualize the effects of climatic variables.

Statistics were calculated using R 3.6.3^66^ and the packages: dplyr^67^, lme4^68^, lmerTest^63^. The fully reproducible code is available in the appendices.

## Results

### Meta-analysis search

A total of 3722 bibliographical references were retrieved using our search strategy. An initial assessment of the relevance of each study was made based on the title and abstract of each paper. This reduced the list to 83 original studies directly relating to the effect of either intercrops or rotation crops on parasitic weed density. After examining the full text of these papers, 67 were deemed to fulfil the inclusion criteria and provide all information needed. The remaining 16 were rejected as having either no experimental control or insufficient detail regarding the effects of response variables. The full list of studies included in the meta-analysis is included in Appendix 2.

**Figure 1:**
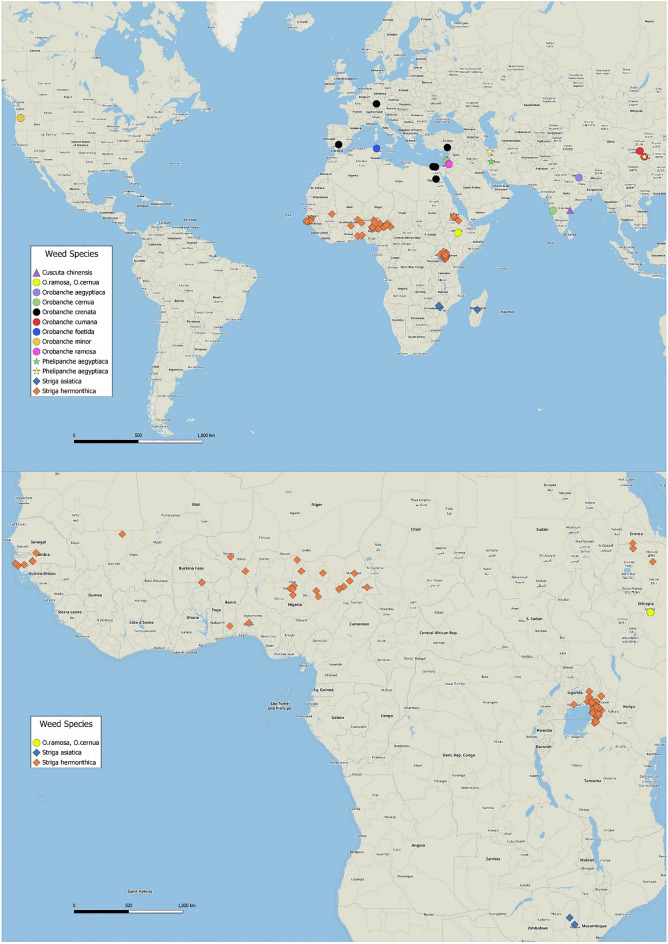
Maps of weed species locations for studies used for this meta-analysis. As the majority of studies focus on sub-Saharan Africa, the lower map has been used to further identify their distribution within this region. Basemap: Open Street Map Basic base map (obtained through QuickMapServices QGIS plugin), Map data: Open StreetMap contributors.

The final dataset encompassed research across 24 countries and 89 localities (Fig. 1) and yielded 1525 individual data points. In terms of weed and crop diversity, it included 11 parasitic weed species, 70 varieties across 18 host crops and 115 intercrop rotation varieties across 105 trap crops (Appendix 3). Contingency tables for both intercrops and rotation crops are shown in Appendix 4.

The studies are predominantly located across sub-Saharan Africa, with a smaller number in North Africa and the Middle East, the Indian subcontinent and China and only three conducted in the United States and Europe. This distribution reflects the severity of the problem of parasitic weeds affecting annual crops across these regions, driving research efforts in search of solutions.

### Cropping system

**Figure 2:**
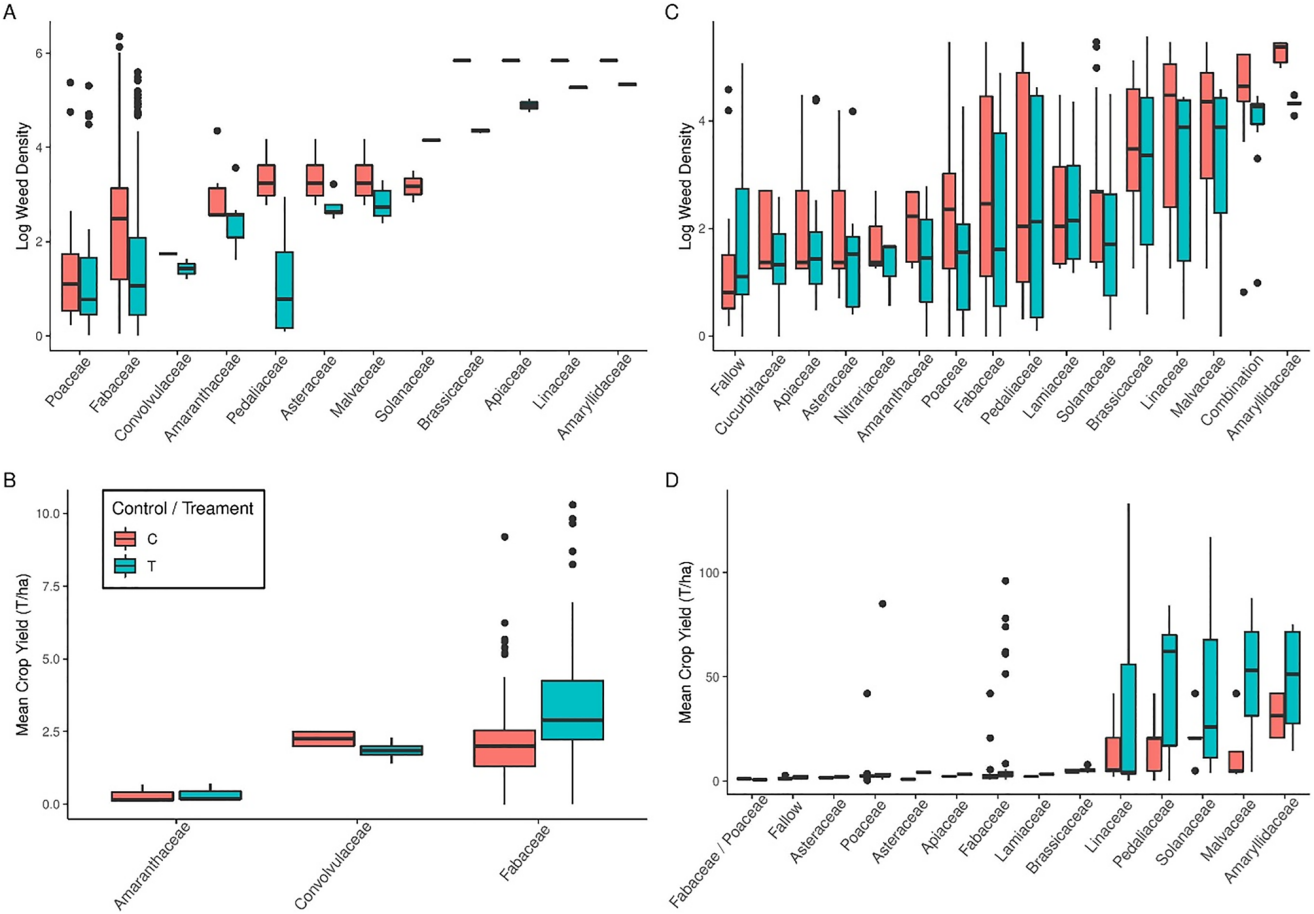
(**A**) Log weed densities in intercrops grouped by family, (**B**) Mean crop yields in intercrops, (**C**) Log weed densities in crop rotation and (**D**) Mean crop yields in rotation crops. Fallow is also included. The same set of figures grouped by crop species are included in Appendix 4.

Our analysis reveals strong overall effects of both intercropping and crop rotation on weed density reduction and crop yields (Table 1). Consistent reductions in weed densities are associated with the use of intercrops across a diverse range of crops (Fig. 2A). Crop yields are also generally higher for intercrops (Fig. 2B), albeit for a smaller range of crop families. The use of multiple crops in the rotation has a consistently negative effect on weed density for a comparably large range of crops (Fig. 2C). Figure 2D indicates higher yields for rotation crops, in particular with respect to *Pedaliaceae* (sesame), *Malvaceae* (cotton) and *Amaryllidaceae* (garlic/onion). Indications of the relative size of these overall effects is provided in Table 2.

**Table 1:** Summary of linear mixed-effects models testing overall effects of cropping system reported across studies. Climatic factors and altitude were tested against non-manipulated, initial weed densities from intercropping and rotation studies in open systems (farm, field trials and landscape). Yield data were obtained from studies with no manipulation of climatic conditions. Significant values are in bold.

Cropping system	Response	Variable	Effect	(df)	P
Intercropping	Weed density	Control/treatment	930.34	1, 596	**< 2.2e−16**
	Yield	Control/treatment	595.07	1, 393	**< 2.2e−16**
Crop rotation	Weed density	Control/treatment	258.03	1, 351	**< 2.2e−16**
	% Change in weed density	Crop diversity	1e−04	1, 110	0.9906
	Yield	Control/treatment	0.1276	1, 112	0.7217
Combined data	Weed density	Rainfall CV	13.6	1, 701	**0.0002**
		Mean rainfall	32.6	1, 701	**1.7e−08**
		Mean temperature	0.4	1, 701	0.5182
		Altitude	14.8	1, 701	**0.0001**
	Yield	Rainfall CV	4.7	1, 488	**0.0311**
		Mean rainfall	6.9	1, 488	**0.0084**
		Mean temperature	14.5	1, 488	**0.0002**
		Altitude	6.8	1, 488	**0.0096**

**Table 2:** Summary of linear mixed-effects models relating parasitic weed density and crop yield to a range of management and ecological predictors with significant probabilities reported in bold. These were used to determine which management factors explained the most variance within models and were, therefore, most significant in influencing both weed density and crop yields. Crop diversity refers to the total number of crops used in rotation.

Cropping system	Response	Variable(s)	Effect	(df)	P
Intercropping	Weed density	Weed species	3.1	7, 56	**0.0086**
		Host crop	3.7	9, 203	**0.0002**
		Intercrop	3.7	34, 170	**7.6e−09**
		Host crop variety	3.7	21, 2	0.2339
		Intercrop variety	0.9	38, 2	0.6436
	Yield	Weed species	2.7	5, 36	**0.0339**
		Host crop	0.4	3, 43	0.7629
		Intercrop	1.7	23, 65	**0.0410**
		Host crop variety	1.2	9, 103	0.2745
		Intercrop variety	1.1	19, 103	0.4510
Crop rotation	Weed density	Weed species	2.1	8,10	0.1255
		Host crop	1.9	7, 15	0.1320
		Rotation crop 1	1.1	81, 217	0.2596
		Crop diversity	0.1	1, 181	0.8965
		Host crop variety	2	15, 43	**0.0439**
		Rotation crop variety 1	0.5	42, 43	0.9826
	Yield	Weed species	1	6, 69	0.4346
		Host crop	0.1	3, 69	0.9441
		Rotation crop 1	0.6	52, 69	0.9824
		Crop diversity	0.5	1, 125	0.503
		Host crop variety	1.5	7, 8	0.2772
		Rotation crop variety 1	0.5	18, 8	0.8637

**Figure 3:**
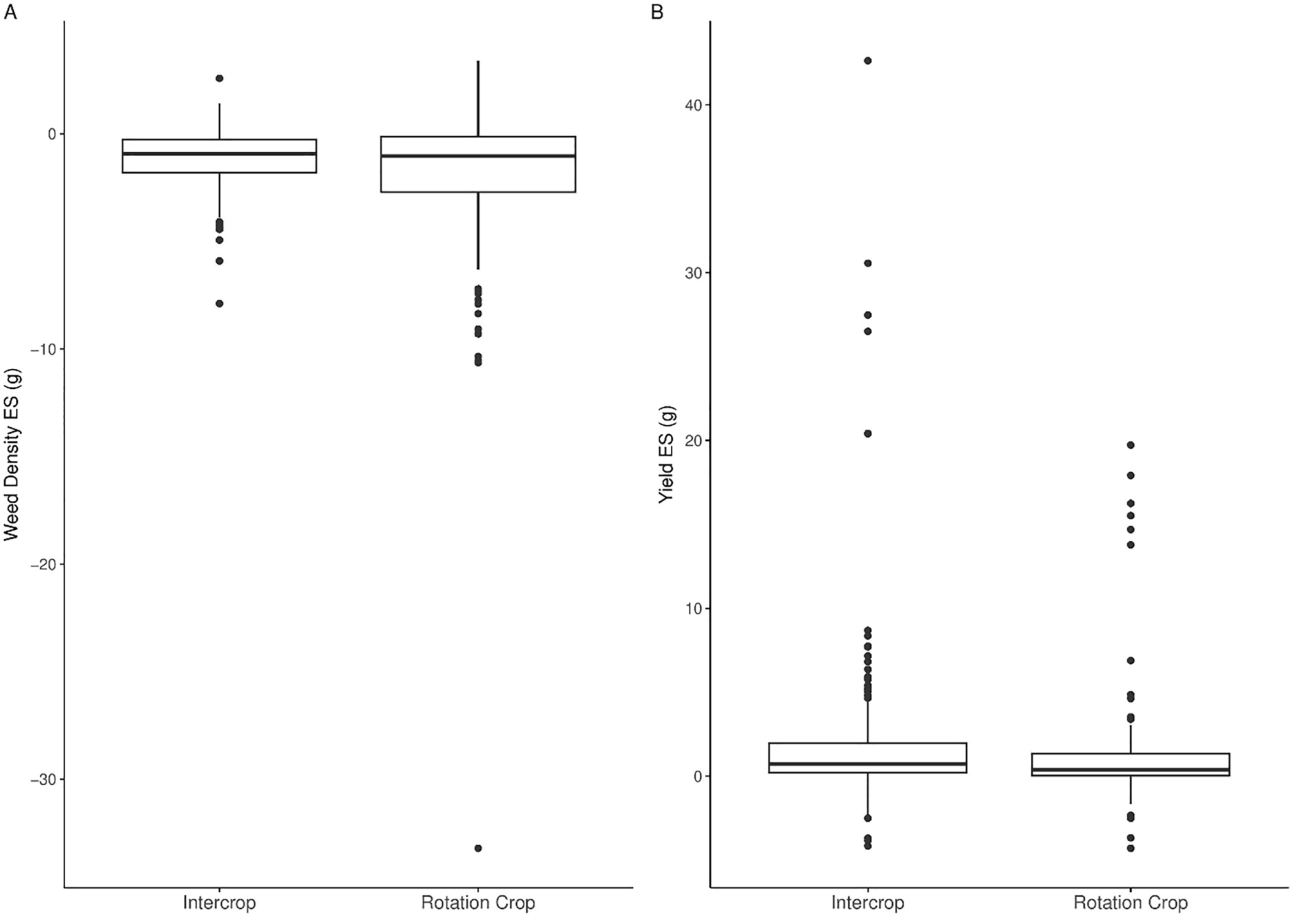
(**A**) The effect of cropping system (intercrop/rotation) on weed density. (**B**) The effect of cropping system (intercrop/rotation) on crop yield with crops grouped by family. Effect size (ES) expressed by Hedges g, multiplied by -1 to aid interpretation.

Analysis of effect sizes (Hedges g) indicated broadly similar mean effect sizes for both systems, with marginally greater weed reduction for rotation cropping and yield increase for intercropping (Fig. 3A,B). The number of crops used in rotation, denoted as diversity, did not have any significant effect on the percentage change in weed density for the mixed effect model (Table 1). Similarly, the mixed effect model for diversity did not show significant differences in weed reduction effect size between the numbers of rotation crops used (Table 2).

### Management factors

The linear mixed-effects models did not detect significant differences in effect sizes for the majority of factors (Table 2). This does not mean the rotation had no effect on the responses, but that effect sizes did not differ greatly enough between the factors. Mean effect sizes for both weed reduction and yield were in fact greater than 0.5 for over 75% of factors tested (Table 2).

**Figure 4:**
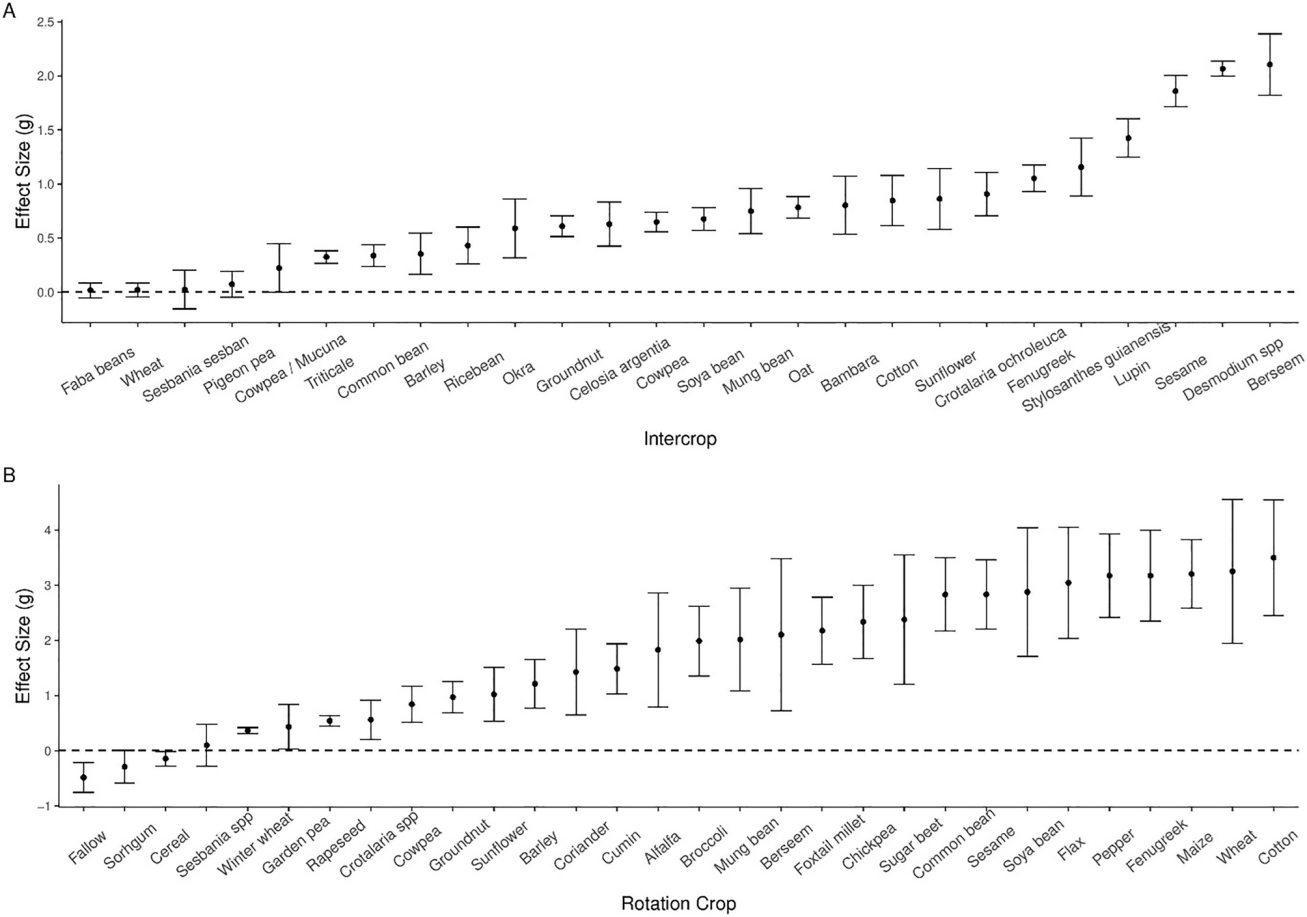
(**A**) Intercrop effects on weed density ordered by effect size ± SE. Faba beans: n = 4, Wheat n = 30, *Sesbania sesban* n = 6, Pigeon pea n = 6, Cowpea/*Mucuna* n = 8, Triticale n = 9, Common bean n = 27, Barley n = 7, Ricebean n = 8, Okra n = 4, Groundnut n = 54, *Celosia argentea* n = 8, Cowpea n = 66, Soya bean n = 21, Mung bean n = 24, Oat n = 21, Bambara n = 9, Cotton n = 4, Sunflower n = 4, *Crotalaria ochroleuca* n = 24, Fenugreek n = 27, *Stylosanthes guianensis* n = 8, Lupin n = 5, Sesame n = 4, *Desmodium *spp. n = 204, Berseem n = 23. (**B**) The effects of rotation crops on crop on weed density ordered by effect size ± SE. Fallow n = 11, Sorhgum n = 7, Cereal n = 9, *Sesbania *spp. n = 11, Winter wheat n = 6, Garden pea n = 4, Rapeseed n = 8, *Crotalaria *spp. n = 4, Cowpea n = 10, Groundnut n = 14, Sunflower n = 4, Barley n = 4, Coriander n = 4, Cumin n = 4, Alfalfa n = 6, Broccoli n = 5, Mung bean n = 4, Berseem n = 6, Foxtail millet n = 6, Chickpea n = 4, Sugar beet n = 6, Common bean n = 8, Sesame n = 10, Soya bean n = 30, Flax n = 8, Pepper n = 13, Fenugreek n = 6, Maize n = 22, Wheat n = 4, Cotton n = 6. Effect size (ES) expressed by Hedges g. Crops with ≤ 3 data points were omitted for concise presentation.

Our models indicated that weed, crop and intercrop species, as well as intercrop variety, had significant effects on weed density effect sizes in intercropping systems. Weed and intercrop species also had a significant effect on yield effect sizes in intercropping systems (Fig. 4A,B). Mixed-effects models for crop rotation also indicated significant effect sizes for weed and host crop species and host crop variety. Notable effects on weed reduction included, inter alia, *Desmodium* and *Stylosanthes* in intercropping and maize, wheat and cotton in rotations. Mixed effect models for factors pertaining to yield in rotation systems did not indicate any individual significance for effect sizes.

### Climatic factors

**Figure 5:**
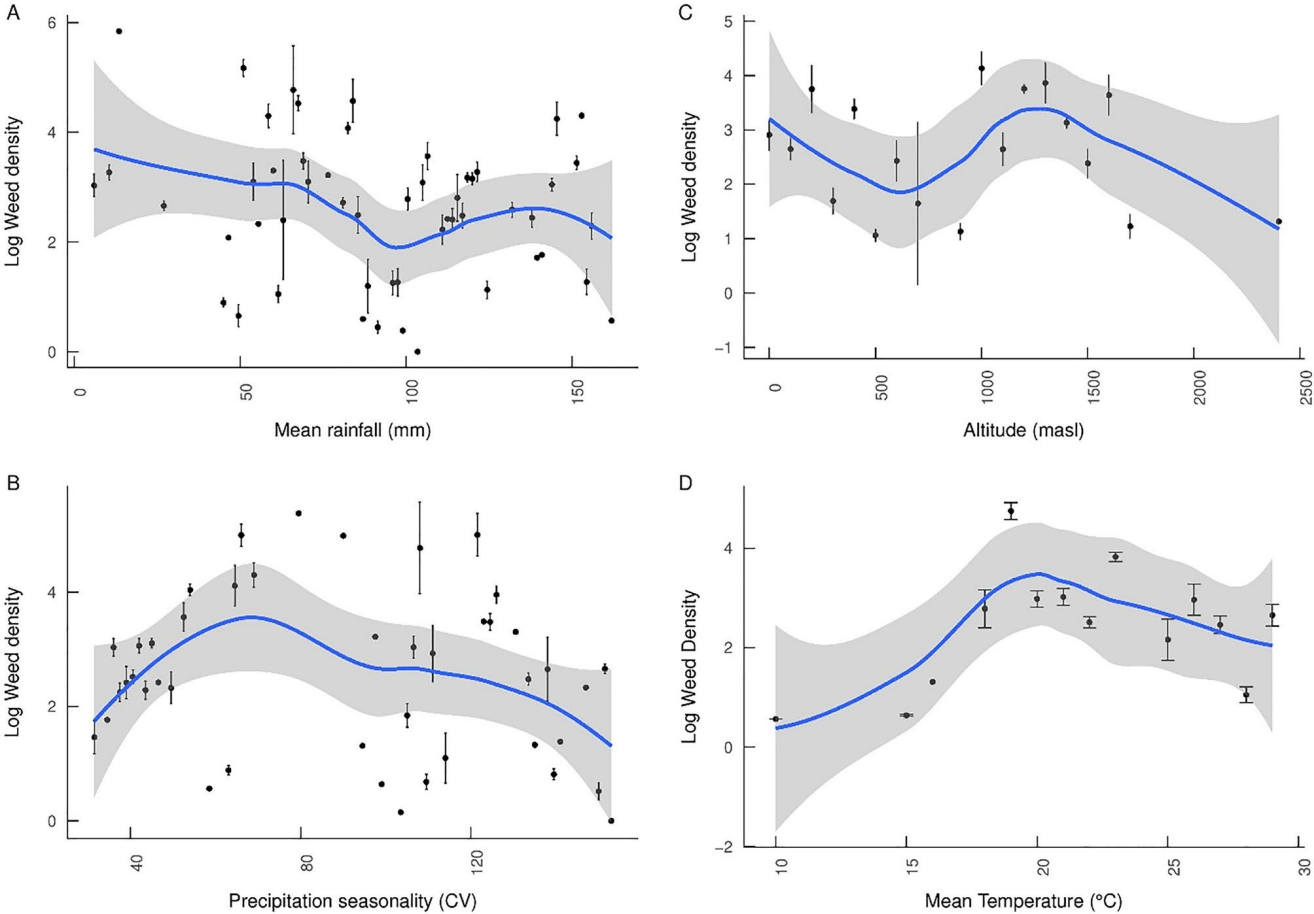
(**A**) Parasitic weed densities and mean annual rainfall ± SE, (**B**) Weed densities and precipitation seasonality (coefficient of variation for rainfall) ± SE, (**C**) Weed densities and altitude ± SE, (**D**) Weed densities and mean annual temperature ± SE. The effects of climatic altitude and altitude on weed densities were significant for several linear models (see Table 2). Data were obtained from non-manipulated initial weed densities in field/farm trials or landscape studies.

In terms of climatic factors, rainfall seasonality (CV), mean annual rainfall and altitude were significant factors for both weed density and yield for intercropping systems (see Table 1 and Fig. 5A,B). For rotation cropping, rainfall seasonality, mean annual rainfall, mean temperature and altitude were significant factors in determining weed density. Mean annual rainfall and mean temperature were significant factors for yields.

A clear negative relationship is seen between log weed density and mean rainfall (Fig. 5A). In addition, an increase in rainfall variability is linked to increases in weed density up to an intermediate level, beyond which densities appear to drop off (Fig. 5B). There are two clear peaks in weed density around zero and 1250 m above sea level, relating generally to the distribution of *Orobanche* and *Striga* species respectively (Fig. 5C). Peak mean temperatures for parasitic weeds is around 20 °C, with very few recorded observations below approximately 17.5 °C. Many species have significant densities in excess of 20 °C however (Fig. 5D).

### Publication bias

Egger’s tests for funnel plot asymmetry indicated a significant degree of heterogeneity within the effect sizes of the data set (random-effects model: p = < 0.0001, mixed-effects meta-regression model p = 0.0449). This indicates that the distribution of effect sizes for studies included in this meta-analysis differs sufficiently from that expected to suggest a bias in the reporting of results. The additional fail-safe N test undertaken indicated however that the impact of any potential bias within the data was low (Rosenberg significance Level = < 0.0001, fail-safe N: 311,129, Rosenthal significance Level = < 0.0001, fail-safe N: 447,309), Orwin fail-safe N = 1517.

## Discussion

Our results demonstrate that crop diversification has consistent effects in reducing parasitic weed density and increasing crop yield. Effects are significant for increases in both spatial (intercropping) and temporal (rotation cropping) crop diversification, though there are notable differences between the two systems. The linear mixed-effects models show the greater effect of weed suppression and yield for intercrops.

The significant effect of crop diversification on weed density is supported by several comparable meta-analyses. For example, in reductions of weed densities in general^7,69^, increased crop yields due to intercropping^19^ and improved yield stability^17^ noted that intercropping. Meta-analyses of agroforestry (which can also be considered a form of diversification) have also found reductions in parasitic and non-parasitic weeds^70^, and crop yield increases^71^.

A recent, meta-analysis of weed responses to crop diversification by Weisberger et al.^7^ found that weed reduction correlated with temporal diversity expressed as the variance of sowing dates between different crops. The metric of temporal crop diversification can encompass elements of intercropping (such as relay cropping) as well as rotation cropping. However, our results suggest that the effects of spatial diversification are stronger than temporal in suppressing parasitic weeds.

Our results further suggests that soil microclimate and host crop pre-attachment resistance effects may be stronger than effects more clearly attributable to rotation such as alteration of soil N_2_. Suicidal germination and allelopathy can occur within both intercropping and rotation cropping systems and could therefore be equally important mechanisms. Different combinations of crops and intercrops will produce different combinations of effects influencing weed density. Intercrops combining strong shading properties and favourably affect soil N_2_ show particularly strong effects in reducing parasitic weed density here, such as *Crotalaria ochroleuca*, *Stylosanthes*, Berseem clover and Lupin. Likewise, crops affording shade with allelopathic properties, antagonistic to parasitic weeds, such as Fenugreek^72^ have large effect sizes in both rotation and intercropping studies. *Desmodium* is effective in three ways, shading, enhancing N_2_ and stimulating suicidal germination by root exudates^72,73^, reflected by its’ significant effect size in this analysis.

Publication bias, in particular the potential over-reporting of significant results, can compromise the validity of the results of meta-analyses^74^. The Egger’s tests undertaken indicated a significant level of potential publication bias within the dataset, supported by the strong concurrence of results from a wide combination of systems, crops and weed species in terms of general trends. Although caution should be exercised in the inference of fail-safe N values, the results of the fail-safe N tests indicate that the data are sufficiently robust in terms of the impact of potential bias^74^.

### Management

Effect sizes for both weed reduction and yield were significant (i.e. nonzero) for all models, and greater than 0.5 for over 75% of factors tested. The most notable effects were those of host crop and host crop variety, intercrop, and to a lesser extent rotation crop. Caution should be exercised with a simplistic, interpretation of effect sizes in terms of small, medium and large in quantitative studies^75^. However, these results clearly show individual crops which perform better than others. The notable effects of crop variety on parasitic weed density support studies of individual parasitic weeds^45–47,76,77^. This effect also supports the rationale of a broader effort to identify and breed crop varieties resistant or tolerant to a wide range of parasitic weed pests^4,78^.

Our models did not detect significant differences in effect sizes for the majority of management factors. This does not indicate these factors should be discounted, but just that effect sizes did not differ greatly enough between the individual elements of these factors. The effects of management factors on yield may not be directly related to weed density, as there is no way to demonstrate the link in this analysis. Other factors are likely involved in influencing yields, as it is clearly understood that different crops, intercrops and crop varieties produce different yields independently of weed density.

### Climate

The significant negative effect of precipitation on parasitic weeds is the most notable climatic effect revealed within the analysis undertaken here. There was also some evidence of a role for precipitation variation. The importance of rainfall and soil moisture is also shown across reviews of future weed distribution trends (e.g.^79^), niche modelling^80–82^ and landscape-scale studies of parasitic weeds^77^. Drier, warmer climates across many areas of Eurasia, South and North America, combined with more erratic rainfall patterns will favour the spread of many of the most problematic parasitic weeds such as *Striga* and *Orobanche*. This underlines the importance of monitoring and biosecurity measures to prevent or contain the introduction into currently uninfested agricultural zones.

## Conclusion

This meta-analysis underlines the important role that temporal and spatial crop diversification has in the reduction of economically important parasitic weeds. This effect is consistent across a wide range of geographic locations, crops, varieties and weed species. There is also strong evidence of the positive effect of diversification on crop yield, although this may involve factors other than weed reduction. This analysis further serves to underline the viability of crop diversification as a tool to enhance global food security. This will become increasingly relevant given projections of the future proliferation of many parasitic weeds to areas currently not under infestation driven by globalisation and climate change.

The concentration of studies undertaken in sub-Saharan Africa indicates, however, that crop diversification is still largely focused on subsistence farmers in the developing world. While this is an entirely valid concentration of efforts, increased research should focus on the effects of diversification on the industrial production of staple crops within agroecological zones possessing Mediterranean climates globally. This will likely happen in response to the evolution of global patterns of weed distributions. However, proactive research strategies informed by predictive risk modelling could help in gaining the upper hand in the crop-weed “arms race”.

## Supplementary Information

Supplementary Information 1.

Supplementary Information 2.

Supplementary Information 3.

Supplementary Information 4.

Supplementary Information 5.

Supplementary Information 6.

Supplementary Information 7.

Supplementary Information 8.

Supplementary Information 9.

Supplementary Information 10.

Supplementary Information 11.

Supplementary Information 12.

Supplementary Information 13.

Supplementary Information 14.

## Data Availability

The datasets generated and/or analysed during the current study are available in the ORDA repository: https://figshare.shef.ac.uk/articles/software/R_Scripts_for_Crop_diversification_and_parasitic_weed_abundance_a_global_meta-analysis/20443896/1 and https://figshare.shef.ac.uk/articles/dataset/Data_files_for_Crop_diversification_and_parasitic_weed_abundance_a_global_meta-analysis/20443923/1.
