# Supplementary material for: Crop diversification and parasitic weed abundance: a global meta-analysis
Source: Sci Rep. 2022 Nov 12;12:19413. doi: 10.1038/s41598-022-24047-2 (PMC9653488; doi:10.1038/s41598-022-24047-2)
Supplement: Supplementary file 1 — Supplementary Information 1. [file 41598_2022_24047_MOESM1_ESM.docx]

## Appendix 2A: Review papers searched

Abang, M.M., Bayaa, B., Abu-Irmaileh, B. and Yahyaoui, A., 2007. A participatory farming system approach for sustainable broomrape (Orobanche spp.) management in the Near East and North Africa. *Crop Protection*, *26*(12), pp.1723-1732.

Awaad, H. and El-Naggar, N., 2018. Potential Role of Intercropping in Maintaining and Facilitating Environmental Sustainability. In *Sustainability of Agricultural Environment in Egypt: Part I* (pp. 81-100). Springer, Cham.

Berner, D.K., Kling, J.G. and Singh, B.B., 1995. Striga research and control. A perspective from Africa. *Plant Disease*, *79*(7), pp.652-660.

Bilalis, D., Papastylianou, P., Konstantas, A., Patsiali, S., Karkanis, A. and Efthimiadou, A., 2010. Weed-suppressive effects of maize–legume intercropping in organic farming. *International Journal of Pest Management*, *56*(2), pp.173-181.

Cimmino, A., Masi, M., Rubiales, D., Evidente, A. and Fernández-Aparicio, M., 2018. Allelopathy for parasitic plant management. *Natural Product Communications*, *13*(3), p.1934578X1801300307.

Goldwasser, Y. and Rodenburg, J., 2013. Integrated agronomic management of parasitic weed seed banks. In *Parasitic Orobanchaceae* (pp. 393-413). Springer, Berlin, Heidelberg.

Habimana, S., Nduwumuremyi, A. and Chinama R, J.D., 2014. Managementof orobanche in field crops: A review. *Journal of soil science and plant nutrition*, *14*(1), pp.43-62.

Hershenhorn, J., Eizenberg, H., Dor, E., Kapulnik, Y. and Goldwasser, Y., 2009. Phelipanche aegyptiaca management in tomato. *Weed research*, *49*, pp.34-47.

Joel, D.M., Hershenhorn, J., Eizenberg, H., Aly, R., Ejeta, G., Rich, P.J., Ransom, J.K., Sauerborn, J. and Rubiales, D., 2007. Biology and management of weedy root parasites. *HORTICULTURAL REVIEWS-WESTPORT THEN NEW YORK-*, *33*, p.267.

Khan, Z.R., Midega, C.A., Bruce, T.J., Hooper, A.M. and Pickett, J.A., 2010. Exploiting phytochemicals for developing a ‘push–pull’crop protection strategy for cereal farmers in Africa. *Journal of experimental botany*, *61*(15), pp.4185-4196.

Mishra, J.S., 2009. Biology and management of Cuscuta species. *Indian Journal of Weed Science*, *41*(1-2), pp.1-11.

Pérez-de-Luque, A., Eizenberg, H., Grenz, J.H., Sillero, J.C., Ávila, C., Sauerborn, J. and Rubiales, D., 2010. Broomrape management in faba bean. *Field crops research*, *115*(3), pp.319-328.

Pickett, J.A., Hamilton, M.L., Hooper, A.M., Khan, Z.R. and Midega, C.A., 2010. Companion cropping to manage parasitic plants. *Annual Review of Phytopathology*, *48*, pp.161-177.

Ransom, J.K., 2000. Long-term approaches for the control of Striga in cereals: field management options. *Crop Protection*, *19*(8-10), pp.759-763.

Restuccia, A., Marchese, M., Mauromicale, G. and Restuccia, G., 2009. Biological characteristics and control of Orobanche crenata Forsk., a review. *Italian Journal of Agronomy*, pp.53-68.

Rubiales, D., Fernández‐Aparicio, M., Wegmann, K. and Joel, D.M., 2009. Revisiting strategies for reducing the seedbank of Orobanche and Phelipanche spp. *Weed Research*, *49*, pp.23-33.

Rubiales, D. and Fernández-Aparicio, M., 2012. Innovations in parasitic weeds management in legume crops. A review. *Agronomy for Sustainable Development*, *32*(2), pp.433-449.

Shekhawat, K., Rathore, S.S., Dass, A., Das, T.K., Mahajan, G. and Chauhan, B.S., 2017. Weed menace and management strategies for enhancing oilseed brassicas production in the Indian sub-continent: A review. *Crop Protection*, *96*, pp.245-257.

Silberg, T.R., Chimonyo, V.G.P., Richardson, R.B., Snapp, S.S. and Renner, K., 2019. Legume diversification and weed management in African cereal-based systems. *Agricultural Systems*, *174*, pp.83-94.

Stoddard, F.L., Nicholas, A.H., Rubiales, D., Thomas, J. and Villegas-Fernández, A.M., 2010. Integrated pest management in faba bean. *Field crops research*, *115*(3), pp.308-318.

## Appendix 2B: Studies Used in Meta-Analysis

Abbes, Z., Trabelsi, I., Kharrat, M. and Amri, M., 2019. Intercropping with fenugreek (Trigonella foenum-graecum) enhanced seed yield and reduced Orobanche foetida infestation in faba bean (Vicia faba). *Biological Agriculture & Horticulture*, *35*(4), pp.238-247.

Abebe, G., Sahile, G. and Al-Tawaha, A.R.M., 2005. Evaluation of potential trap crops on Orobanche soil seed bank and tomato yield in the central rift valley of Ethiopia. *World Journal of Agricultural Sciences*, *1*(2), pp.148-151.

Abu-Irmaileh, B.E., 1984, May. Effect of planting flax on subsequent infestation of tomato by Orobanche ramosa. In *Proceedings* (pp. 250-255).

Abunyewa, A.A. and Padi, F.K., 2003. Changes in soil fertility and Striga hermonthica prevalence associated with legume and cereal cultivation in the Sudan savannah zone of Ghana. *Land Degradation & Development*, *14*(3), pp.335-343.

Abu-Shall, A.M.H. and Ragheb, E.I.M., 2014. Management of Orobanche crenata Using Trap Crops and Phytomyza orobanchia Kalt. in Broad Bean (Vicia faba) Field in Egypt. *Egyptian Journal of Biological Pest Control*, *24*(1).

Acharya, B.D., Khattri, G.B., Chettri, M.K. and Srivastava, S.C., 2002. Effect of Brassica campestris var. toria as a catch crop on Orobanche aegyptiaca seed bank. *Crop protection*, *21*(7), pp.533-537.

Ajeigbe, H.A., Ndaghu, N., Kamsang, L., Ademulegun, T. and Solomon, R., 2019. Using a participatory approach and legume integration to increase the productivity of early maturing maize in the Nigerian Sudan Savannas. *International Journal of Agronomy*, *2019*.

Aksoy, E., Arslan, Z.F., Tetik, Ö. and Eymirli, S., 2016. Using the possibilities of some trap, catch and Brassicaceaen crops for controlling crenate broomrape a problem in lentil fields. *International Journal of Plant Production*, *10*(1), pp.53-62.

Babaei, S., Alizadeh, H., Jahansouz, M.R., Mashhadi, H.R. and Moeini, M.M., 2010. Management of'Phelipanche aegyptiaca'Pomel: Using Trap Crops in Rotation with Tomato ('Solanum lycopersicom'L.). *Australian Journal of Crop Science*, *4*(6), pp.437-442.

Bakheit, B.R., Allam, A.Y. and Galal, A.H., 2002. Intercropping faba bean with some legume crops for control of Orobanche crenata. *Acta Agronomica Hungarica*, *50*(1), pp.1-6.

Birhane, E., Gebremeskel, K., Taddesse, T., Hailemariam, M., Hadgu, K.M., Norgrove, L. and Negussie, A., 2018. Integrating Faidherbia albida trees into a sorghum field reduces striga infestation and improves mycorrhiza spore density and colonization. *Agroforestry Systems*, *92*(3), pp.643-653.

Carsky, R.J., Singh, L. and Ndikawa, R., 1994. Suppression of Striga hermonthica on sorghum using a cowpea intercrop. *Experimental Agriculture*, *30*(3), pp.349-358.

Carsky, R.J., Berner, D.K., Oyewole, B.D., Dashiell, K. and Schulz, S., 2000. Reduction of Striga hermonthica parasitism on maize using soybean rotation. *International journal of pest management*, *46*(2), pp.115-120.

Carson, A.G., 1988. Detailed survey of the parasitic weed Striga hermonthica in The Gambia. *International Journal of Pest Management*, *34*(2), pp.162-164.

Carson, A.G., 1989. Effect of intercropping sorghum and groundnuts on density of Striga hermonthica in the Gambia. *International Journal of Pest Management*, *35*(2), pp.130-132.

Chivinge, O. A., E. Kasembe, I. K. Mariga, and S. Mabasa. "The effect of different cowpea cultivars on witchweed and maize yield under dryland conditions." In *The BCPC Conference: Weeds, 2001, Volume 1 and Volume 2. Proceedings of an international conference held at the Brighton Hilton Metropole Hotel, Brighton, UK, 12-15 November 2001*, pp. 163-168. British Crop Protection Council, 2001.

Dhanapal, G.N. 1996. Management of broomrape (Orobanche cernua) in tobacco (Nicotiana tabacum). PhD thesis. p 183 University of Wageningen, NL

Dugje, I.Y., Kamara, A.Y. and Omoigui, L.O., 2008. Influence of FarmersCrop Management Practices on Striga hermonthica Infestation and Grain Yield of Maize (Zea mays L.) in the Savanna Zones of Northeast Nigeria. *Journal of agronomy*.

Ellis-Jones, J., Schulz, S., Douthwaite, B., Hussaini, M.A., Oyewole, B.D., Olanrewaju, A.S. and White, R., 2004. An assessment of integrated Striga hermonthica control and early adoption by farmers in northern Nigeria.

Fenández-Aparicio, M., Sillero, J.C. and Rubiales, D., 2007. Intercropping with cereals reduces infection by Orobanche crenata in legumes. *Crop protection*, *26*(8), pp.1166-1172.

Fernández-Aparicio, M., Emeran, A.A. and Rubiales, D., 2008. Control of Orobanche crenata in legumes intercropped with fenugreek (Trigonella foenum-graecum). *Crop protection*, *27*(3-5), pp.653-659.

Fernández-Aparicio, M., Emeran, A.A. and Rubiales, D., 2010. Inter-cropping with berseem clover (Trifolium alexandrinum) reduces infection by Orobanche crenata in legumes. *Crop protection*, *29*(8), pp.867-871.

Franke, A.C., Ellis-Jones, J., Tarawali, G., Schulz, S., Hussaini, M.A., Kureh, I., White, R., Chikoye, D., Douthwaite, B., Oyewole, B.D. and Olanrewaju, A.S., 2006. Evaluating and scaling-up integrated Striga hermonthica control technologies among farmers in northern Nigeria. *Crop protection*, *25*(8), pp.868-878.

Gacheru, E. and Rao, M.R., 2005. The potential of planted shrub fallows to combat Striga infestation on maize. *International Journal of Pest Management*, *51*(2), pp.91-100.

Hershenhorn, J., Goldwasser, Y., Plakhine, D., Herzlinger, G., Golan, S., Russo, R. and Kleifeld, Y., 1996. Role of pepper (Capsicum annuum) as a trap and catch crop for control of Orobanche aegyptiaca and O. cernua. *Weed science*, pp.948-951.

Hailu, G., Niassy, S., Zeyaur, K.R., Ochatum, N. and Subramanian, S., 2018. Maize–legume intercropping and push–pull for management of fall armyworm, stemborers, and striga in Uganda. Agronomy Journal, 110(6), pp.2513-2522.

Hayat, S., Wang, K., Liu, B., Wang, Y., Chen, F., Li, P., Hayat, K. and Ma, Y., 2020. A Two-Year Simulated Crop Rotation Confirmed the Differential Infestation of Broomrape Species in China Is Associated with Crop-Based Biostimulants. *Agronomy*, *10*(1), p.18.

Hess, D.E. and Dodo, H., 2004. Potential for sesame to contribute to integrated control of Striga hermonthica in the West African Sahel. *Crop Protection*, *23*(6), pp.515-522.

Hudu, A.I., 1998. Preliminary results on evaluation of trap crops for Striga hermonthica (Del.) Benth. control in sorghum. *Int. Sorghum Millets Newslett.*, *39*, pp.118-121.

Kamara, A.Y., Ellis-Jones, J., Amaza, P., Omoigui, L.O., Helsen, J., Dugje, I.Y., Kamai, N., Menkir, A. and White, R.W., 2008. A participatory approach to increasing productivity of maize through Striga hermonthica control in northeast Nigeria. *Experimental Agriculture*, *44*(3), p.349.

Kanampiu, F., Makumbi, D., Mageto, E., Omanya, G., Waruingi, S., Musyoka, P. and Ransom, J., 2018. Assessment of management options on Striga infestation and maize grain yield in Kenya. *Weed Science*, *66*(4), pp.516-524.

Khan, Z.R., Pickett, J.A., Wadhams, L. and Muyekho, F., 2001. Habitat management strategies for the control of cereal stemborers and striga in maize in Kenya. *International Journal of Tropical Insect Science*, *21*(4), pp.375-380.

Khan, Z.R., Hassanali, A., Overholt, W., Khamis, T.M., Hooper, A.M., Pickett, J.A., Wadhams, L.J. and Woodcock, C.M., 2002. Control of witchweed Striga hermonthica by intercropping with Desmodium spp., and the mechanism defined as allelopathic. *Journal of chemical ecology*, *28*(9), pp.1871-1885.

Khan, Z.R., Midega, C.A., Hassanali, A., Pickett, J.A. and Wadhams, L.J., 2007. Assessment of different legumes for the control of Striga hermonthica in maize and sorghum. *Crop Science*, *47*(2), pp.730-734. Uganda. *Agronomy Journal*, *110*(6), pp.2513-2522.

Khan, Z.R., Midega, C.A., Hassanali, A., Pickett, J.A., Wadhams, L.J. and Wanjoya, A., 2006. Management of witchweed, Striga hermonthica, and stemborers in sorghum, Sorghum bicolor, through intercropping with greenleaf desmodium, Desmodium intortum. *International Journal of Pest Management*, *52*(4), pp.297-302.

Khan, Z.R., Midega, C.A., Amudavi, D.M., Hassanali, A. and Pickett, J.A., 2008. On-farm evaluation of the ‘push–pull’technology for the control of stemborers and striga weed on maize in western Kenya. *Field Crops Research*, *106*(3), pp.224-233.

Khan, Z.R., Midega, C.A., Wanyama, J.M., Amudavi, D.M., Hassanali, A., Pittchar, J. and Pickett, J.A., 2009. Integration of edible beans (Phaseolus vulgaris L.) into the push–pull technology developed for stemborer and Striga control in maize-based cropping systems. *Crop Protection*, *28*(11), pp.997-1006.

Kleifeld, Y., Goldwasser, Y., Herzlinger, G., Joel, D.M., Golan, S. and Kahana, D., 1994. The effects of flax (Linum usitatissimum L.) and other crops as trap and catch crops for control of Egyptian broomrape (Orobanche aegyptiaca Pers.). *Weed Research*, *34*(1), pp.37-44.

Kuchinda, N.C., Kureh, I., Tarfa, B.D., Shinggu, C. and Omolehin, R., 2003. On-farm evaluation of improved maize varieties intercropped with some legumes in the control of Striga in the Northern Guinea savanna of Nigeria. *Crop protection*, *22*(3), pp.533-538.

Kureh, I., Kamara, A.Y. and Tarfa, B.D., 2006. Influence of cereal-legume rotation on Striga control and maize grain yield in farmers’ fields in the Northern Guinea savanna of Nigeria. *Journal of Agriculture and Rural Development in the Tropics and Subtropics (JARTS)*, *107*(1), pp.41-54.

Lins, R.D., Colquhoun, J.B. and Mallory‐Smith, C.A., 2006. Investigation of wheat as a trap crop for control of Orobanche minor. *Weed research*, *46*(4), pp.313-318.

Midega, C.A., Khan, Z.R., Amudavi, D.M., Pittchar, J. and Pickett, J.A., 2010. Integrated management of Striga hermonthica and cereal stemborers in finger millet (Eleusine coracana (L.) Gaertn.) through intercropping with Desmodium intortum. *International journal of pest management*, *56*(2), pp.145-151.

Midega, C.A., Pittchar, J., Salifu, D., Pickett, J.A. and Khan, Z.R., 2013. Effects of mulching, N-fertilization and intercropping with Desmodium uncinatum on Striga hermonthica infestation in maize. *Crop protection*, *44*, pp.44-49.

Midega, C.A., Salifu, D., Bruce, T.J., Pittchar, J., Pickett, J.A. and Khan, Z.R., 2014. Cumulative effects and economic benefits of intercropping maize with food legumes on Striga hermonthica infestation. *Field Crops Research*, *155*, pp.144-152.

Midega, C.A., Bruce, T.J., Pickett, J.A., Pittchar, J.O., Murage, A. and Khan, Z.R., 2015. Climate-adapted companion cropping increases agricultural productivity in East Africa. *Field Crops Research*, *180*, pp.118-125.

Murdoch, A.J. and Kunjo, E.M., 2003. Depletion of natural soil seedbanks of Striga hermonthica in West Africa under different integrated management regimes.

Musambasi, D., Chivinge, O.A. and Mariga, I.K., 2002. Intercropping maize with grain legumes for Striga control in Zimbabwe. *Afr. Crop Sci. J*, *10*(2), pp.163-171.

Odhiambo, J.A., Vanlauwe, B., Tabu, I.M., Kanampiu, F. and Khan, Z., 2011. Effect of intercropping maize and soybeans on Striga hermonthica parasitism and yield of maize. *Archives of Phytopathology and Plant Protection*, *44*(2), pp.158-167.

Olupot, J.R., Osiru, D.S.O., Oryokot, J. and Gebrekidan, B., 2003. The effectiveness of Celosia argentia (Striga “chaser”) to control Striga on sorghum in Uganda. *Crop protection*, *22*(3), pp.463-468.

Oswald, A. and Ransom, J.K., 2001. Striga control and improved farm productivity using crop rotation. *Crop Protection*, *20*(2), pp.113-120.

Oswald, A., Ransom, J.K., Kroschel, J. and Sauerborn, J., 2002. Intercropping controls Striga in maize based farming systems. *Crop Protection*, *21*(5), pp.367-374.

Parkinson, V., Kim, S.K., Efron, Y., Bello, L. and Dashiell, K., 1989. Potential trap crops as a cultural measure in Striga control for Africa. *Striga-improved Management in Africa*, pp.136-140.

Qasem, J.R., 2019. Branched broomrape (Orobanche ramosa L.) control in tomato (Lycopersicon esculentum Mill.) by trap crops and other plant species in rotation. *Crop Protection*, *120*, pp.75-83.

Randrianjafizanaka, Meva Tahiry, Patrice Autfray, Alain Paul Andrianaivo, Isabelle Ratsimiala Ramonta, and Jonne Rodenburg. "Combined effects of cover crops, mulch, zero-tillage and resistant varieties on Striga asiatica (L.) Kuntze in rice-maize rotation systems." *Agriculture, Ecosystems & Environment* 256 (2018): 23-33.

Rao, P.N. and Reddy, A.R.S., 1987. Effect of china dodder on two pulses: green gram and cluster bean-the latter a possible trap crop to manage china dodder. In *Proceedings of the 4th international symposium on parasitic flowering plants.* (pp. 665-674).

Razavifar, Z., Karimmojeni, H. and Sini, F.G., 2017. Effects of wheat-canola intercropping on Phelipanche aegyptiaca parasitism. *Journal of plant protection research*, *57*(3).

Reda, F., Verkleij, J.A.C. and Ernst, W.H.O., 2005. Relay cropping of sorghum and legume shrubs for crop yield improvement and Striga control in the subsistence agriculture region of Tigray (northern Ethiopia). *Journal of agronomy and crop science*, *191*(1), pp.20-26.

Samake, O., Stomph, T.J., Kropff, M.J. and Smaling, E.M.A., 2006. Integrated pearl millet management in the Sahel: Effects of legume rotation and fallow management on productivity and Striga hermonthica infestation. *Plant and soil*, *286*(1), pp.245-257.

Schnell, H., Linke, K.H. and Sauerborn, J., 1994. Trap cropping and its effect on yield and Orobanche crenata Forsk. infestation on following pea (Pisum sativum L.) crops. *Tropical Science*.

Schulz, S., Hussaini, M.A., Kling, J.G., Berner, D.K. and Ikie, F.O., 2003. Evaluation of integrated Striga hermonthica control technologies under farmer management. *Experimental Agriculture*, *39*(1), p.99.

Sjögren, H., Shepherd, K.D. and Karlsson, A., 2010. Effects of improved fallow with Sesbania sesban on maize productivity and Striga hermonthica infestation in Western Kenya. *Journal of Forestry Research*, *21*(3), pp.379-386.

Tenebe, V.A. and Kamara, H.M., 2002. Effect of Striga hermonthica on the growth characteristics of sorghum intercropped with groundnut varieties. *Journal of agronomy and crop science*, *188*(6), pp.376-381.

Vissoh, P.V., Gbehounou, G., Ahanchede, A., Röling, N.G. and Kuyper, T.W., 2008. Evaluation of integrated crop management strategies employed to cope with Striga infestation in permanent land use systems in southern Benin. *International Journal of Pest Management*, *54*(3), pp.197-206.

Webb, M., Togola, A. and Traore, D., 1993. Effect of intercropping pearl millet and cowpea on the density of Striga hermonthica in Mali. In BRIGHTON CROP PROTECTION CONFERENCE WEEDS (Vol. 2, pp. 925-925). BRIT CROP PROTECTION COUNCIL.

Weisskopf, L., Akello, P., Milleret, R., Khan, Z.R., Schulthess, F., Gobat, J.M. and Le Bayon, R.C., 2009. White lupin leads to increased maize yield through a soil fertility-independent mechanism: a new candidate for fighting Striga hermonthica infestation?. *Plant and soil*, *319*(1), pp.101-114.

Ye, X., Chen, J., McErlean, C.S., Zhang, M., Yu, R. and Ma, Y., 2017. The potential of foxtail millet as a trap crop for sunflower broomrape. *Acta Physiologiae Plantarum*, *39*(1), pp.1-11.

Ye, X., Zhang, M., Zhang, M. and Ma, Y., 2020. Assessing the Performance of Maize (Zea mays L.) as Trap Crops for the Management of Sunflower Broomrape (Orobanche cumana Wallr.). *Agronomy*, *10*(1), p.100.
