# Supplementary material for: Crop diversification and parasitic weed abundance: a global meta-analysis
Source: Sci Rep. 2022 Nov 12;12:19413. doi: 10.1038/s41598-022-24047-2 (PMC9653488; doi:10.1038/s41598-022-24047-2)
Supplement: Supplementary file 2 — Supplementary Information 2. [file 41598_2022_24047_MOESM2_ESM.docx]

# MIXED EFFECT MODELS

# Model 1

Type III Analysis of Variance Table with Satterthwaite's method

Sum Sq Mean Sq NumDF DenDF F value Pr(>F)

W_SP 31.859 4.5512 7 56.545 3.0454 0.0086441

HC_SP 49.883 5.5425 9 203.459 3.7088 0.0002497

IC_SP 188.836 5.5540 34 170.500 3.7164 7.565e-09

W_SP **

HC_SP ***

IC_SP ***

---

Signif. codes:

0 ‘***’ 0.001 ‘**’ 0.01 ‘*’ 0.05 ‘.’ 0.1 ‘ ’ 1

> summary(mixed.mod1)

Linear mixed model fit by REML. t-tests use

Satterthwaite's method [lmerModLmerTest]

Formula:

HEDGES ~ W_SP + HC_SP + IC_SP + (1 | Study_ID)

Data: MST_IC_ASD_IMP_WD

Weights: 1/VAR_G

REML criterion at convergence: 1284.1

Scaled residuals:

Min 1Q Median 3Q Max

-3.3778 -0.3560 -0.0084 0.4510 4.2252

Random effects:

Groups Name Variance Std.Dev.

Study_ID (Intercept) 0.124 0.3522

Residual 1.494 1.2225

Number of obs: 622, groups: Study_ID, 39

Fixed effects:

Estimate Std. Error

(Intercept) 1.07000 0.83182

W_SPO. aegyptiaca -2.47000 1.20151

W_SPO. cernua -3.65000 1.34250

W_SPO. crenata -0.92144 1.37885

W_SPO. foetida -1.39432 1.46916

W_SPPhelipanche aegyptiaca -1.05351 0.91526

W_SPS. asiatica 0.09747 0.92635

W_SPS. hermonthica 0.17781 0.88778

HC_SPChickling pea -0.10282 0.76906

HC_SPFaba bean 0.18700 0.70372

HC_SPFinger millet 1.87438 0.87338

HC_SPGarden pea -0.75966 0.69921

HC_SPLentil 0.57789 0.94482

HC_SPMaize -0.68027 0.17292

HC_SPPea 0.15458 0.73374

HC_SPPearl millet -0.29595 0.52189

HC_SPRice -0.34839 0.32878

IC_SPBarley 0.06137 0.97594

IC_SPBerseem 1.50224 0.87695

IC_SPCelery 0.96684 1.09917

IC_SPCelosia argentia -0.67012 0.46467

IC_SPCommon bean -0.21022 0.33029

IC_SPCotton -0.41612 0.50912

IC_SPCowpea -0.07498 0.29388

IC_SPCowpea / Mucuna -0.46466 0.58308

IC_SPCrotalaria ochroleuca 0.23295 0.34012

IC_SPCrotolaria juncea -0.10596 0.77311

IC_SPD.intortum 1.08782 0.31638

IC_SPD.uncinatum 1.06314 0.32392

IC_SPDesmodium / Common bean 0.90267 0.75497

IC_SPDesmodium spp 0.91406 0.75715

IC_SPFaba beans -0.20715 0.42074

IC_SPFaidherbia albida 2.37219 0.96829

IC_SPFenugreek 0.93734 0.87481

IC_SPFlax 0.57870 1.07877

IC_SPGarlic 0.56030 1.07710

IC_SPGroundnut -0.11043 0.29684

IC_SPLupin 1.07246 0.76633

IC_SPMung bean 0.06824 0.33921

IC_SPOat 0.43400 0.95580

IC_SPOkra -0.68197 0.50155

IC_SPPigeon pea -1.17781 0.52007

IC_SPRadish 1.52199 1.14081

IC_SPRicebean -0.24676 0.58144

IC_SPSesame 0.61495 0.56632

IC_SPSesbania sesban -1.22412 0.52125

IC_SPSoya bean -0.14350 0.32751

IC_SPStylosanthes guianensis 0.38199 0.59010

IC_SPSunflower -0.41249 0.51169

IC_SPSweet potao 0.22957 0.70288

IC_SPTriticale 0.01527 0.96764

df t value

(Intercept) 242.80991 1.286

W_SPO. aegyptiaca 256.69629 -2.056

W_SPO. cernua 332.06707 -2.719

W_SPO. crenata 283.12942 -0.668

W_SPO. foetida 227.59794 -0.949

W_SPPhelipanche aegyptiaca 110.16346 -1.151

W_SPS. asiatica 162.98417 0.105

W_SPS. hermonthica 244.63940 0.200

HC_SPChickling pea 563.69205 -0.134

HC_SPFaba bean 570.54208 0.266

HC_SPFinger millet 243.39312 2.146

HC_SPGarden pea 553.13965 -1.086

HC_SPLentil 568.19561 0.612

HC_SPMaize 112.64481 -3.934

HC_SPPea 570.93435 0.211

HC_SPPearl millet 41.29938 -0.567

HC_SPRice 485.51812 -1.060

IC_SPBarley 95.22443 0.063

IC_SPBerseem 217.53513 1.713

IC_SPCelery 276.60065 0.880

IC_SPCelosia argentia 215.28930 -1.442

IC_SPCommon bean 504.99715 -0.636

IC_SPCotton 553.41737 -0.817

IC_SPCowpea 464.38062 -0.255

IC_SPCowpea / Mucuna 32.51954 -0.797

IC_SPCrotalaria ochroleuca 520.94969 0.685

IC_SPCrotolaria juncea 519.34167 -0.137

IC_SPD.intortum 404.04972 3.438

IC_SPD.uncinatum 350.54714 3.282

IC_SPDesmodium / Common bean 362.90340 1.196

IC_SPDesmodium spp 364.81047 1.207

IC_SPFaba beans 568.22564 -0.492

IC_SPFaidherbia albida 303.01772 2.450

IC_SPFenugreek 219.69991 1.071

IC_SPFlax 264.00157 0.536

IC_SPGarlic 262.96158 0.520

IC_SPGroundnut 506.18816 -0.372

IC_SPLupin 167.19635 1.399

IC_SPMung bean 520.42891 0.201

IC_SPOat 88.26966 0.454

IC_SPOkra 551.21141 -1.360

IC_SPPigeon pea 38.25316 -2.265

IC_SPRadish 301.92318 1.334

IC_SPRicebean 32.16011 -0.424

IC_SPSesame 564.49407 1.086

IC_SPSesbania sesban 38.59642 -2.348

IC_SPSoya bean 530.90934 -0.438

IC_SPStylosanthes guianensis 34.09293 0.647

IC_SPSunflower 554.12109 -0.806

IC_SPSweet potao 458.05229 0.327

IC_SPTriticale 92.32303 0.016

Pr(>|t|)

(Intercept) 0.199551

W_SPO. aegyptiaca 0.040819 *

W_SPO. cernua 0.006896 **

W_SPO. crenata 0.504510

W_SPO. foetida 0.343596

W_SPPhelipanche aegyptiaca 0.252206

W_SPS. asiatica 0.916332

W_SPS. hermonthica 0.841426

HC_SPChickling pea 0.893695

HC_SPFaba bean 0.790543

HC_SPFinger millet 0.032852 *

HC_SPGarden pea 0.277754

HC_SPLentil 0.541022

HC_SPMaize 0.000145 ***

HC_SPPea 0.833212

HC_SPPearl millet 0.573734

HC_SPRice 0.289823

IC_SPBarley 0.949989

IC_SPBerseem 0.088132 .

IC_SPCelery 0.379834

IC_SPCelosia argentia 0.150710

IC_SPCommon bean 0.524751

IC_SPCotton 0.414087

IC_SPCowpea 0.798728

IC_SPCowpea / Mucuna 0.431286

IC_SPCrotalaria ochroleuca 0.493714

IC_SPCrotolaria juncea 0.891040

IC_SPD.intortum 0.000646 ***

IC_SPD.uncinatum 0.001134 **

IC_SPDesmodium / Common bean 0.232619

IC_SPDesmodium spp 0.228123

IC_SPFaba beans 0.622653

IC_SPFaidherbia albida 0.014856 *

IC_SPFenugreek 0.285133

IC_SPFlax 0.592103

IC_SPGarlic 0.603368

IC_SPGroundnut 0.710043

IC_SPLupin 0.163522

IC_SPMung bean 0.840637

IC_SPOat 0.650892

IC_SPOkra 0.174478

IC_SPPigeon pea 0.029278 *

IC_SPRadish 0.183164

IC_SPRicebean 0.674101

IC_SPSesame 0.277997

IC_SPSesbania sesban 0.024073 *

IC_SPSoya bean 0.661440

IC_SPStylosanthes guianensis 0.521751

IC_SPSunflower 0.420517

IC_SPSweet potao 0.744109

IC_SPTriticale 0.987443

# Model 2

# Type III Analysis of Variance Table with Satterthwaite's method

# Sum Sq Mean Sq NumDF DenDF F value Pr(>F)

# HC_V 43.228 2.0585 21 2 3.7056 0.2339

# IC_V 19.912 0.5240 38 2 0.9433 0.6436

# > summary(mixed.mod4)

# Linear mixed model fit by REML. t-tests use

# Satterthwaite's method [lmerModLmerTest]

# Formula: HEDGES ~ HC_V + IC_V + (1 | Study_ID)

# Data: MST_IC_ASD_IMP_WD

# Weights: 1/VAR_G

# REML criterion at convergence: 238.6

# Scaled residuals:

# Min 1Q Median 3Q Max

# -3.1233 -0.2659 0.0000 0.2672 4.3923

# Random effects:

# Groups Name Variance Std.Dev.

# Study_ID (Intercept) 0.04328 0.2080

# Residual 0.55551 0.7453

# Number of obs: 207, groups: Study_ID, 18

# Fixed effects:

# Estimate Std. Error df t value

# (Intercept) 9.500e-02 2.962e-01 1.986e-09 0.321

# HC_VHCV21 9.500e-02 2.981e-01 1.470e+02 0.319

# HC_VHCV22 3.818e-01 5.009e-01 4.063e-09 0.762

# HC_VHCV23 2.712e-01 4.243e-01 2.092e-09 0.639

# HC_VHCV25 1.195e+00 5.885e-01 7.741e-09 2.031

# HC_VHCV27 1.473e-01 3.798e-01 1.343e-09 0.388

# HC_VHCV28 4.518e-01 4.920e-01 3.780e-09 0.918

# HC_VHCV29 -7.226e-02 4.564e-01 2.800e-09 -0.158

# HC_VHCV30 9.000e-01 4.511e-01 2.671e-09 1.995

# HC_VHCV31 1.545e+00 5.195e-01 4.700e-09 2.974

# HC_VHCV32 2.450e-01 5.405e-01 5.506e-09 0.453

# HC_VHCV33 2.350e-01 5.087e-01 4.321e-09 0.462

# HC_VHCV37 8.202e-01 5.017e-01 4.087e-09 1.635

# HC_VHCV38 4.747e-01 4.092e-01 1.810e-09 1.160

# HC_VHCV51 8.022e-01 4.408e-01 2.435e-09 1.820

# HC_VHCV57 1.491e+00 4.170e-01 1.951e-09 3.576

# HC_VHCV58 2.598e-01 4.035e-01 1.711e-09 0.644

# HC_VHCV59 1.977e-01 4.036e-01 1.713e-09 0.490

# HC_VHCV60 5.894e-01 4.159e-01 1.930e-09 1.417

# HC_VHCV61 -1.954e+00 5.231e-01 4.830e-09 -3.736

# HC_VHCV62 -1.500e-02 5.004e-01 4.047e-09 -0.030

# HC_VHCV7 9.661e-01 4.171e-01 1.953e-09 2.316

# IC_VICV11 6.990e-02 1.632e-01 1.470e+02 0.428

# IC_VICV12 -1.423e-01 1.627e-01 1.470e+02 -0.875

# IC_VICV13 2.067e-01 4.756e-01 1.470e+02 0.435

# IC_VICV14 9.547e-02 4.726e-01 1.470e+02 0.202

# IC_VICV15 5.462e-02 4.712e-01 1.470e+02 0.116

# IC_VICV17 1.113e+00 4.030e-01 1.470e+02 2.763

# IC_VICV18 2.457e-02 3.814e-01 1.470e+02 0.064

# IC_VICV19 3.211e-01 3.996e-01 1.470e+02 0.804

# IC_VICV22 6.700e-01 5.164e-01 1.470e+02 1.298

# IC_VICV25 -9.511e-01 4.673e-01 3.078e-09 -2.035

# IC_VICV26 3.240e-01 4.592e-01 2.869e-09 0.705

# IC_VICV27 -1.046e-01 2.272e-01 1.470e+02 -0.460

# IC_VICV30 -4.516e-01 4.800e-01 1.470e+02 -0.941

# IC_VICV31 -2.291e-01 4.853e-01 1.470e+02 -0.472

# IC_VICV32 -2.012e-02 4.930e-01 1.470e+02 -0.041

# IC_VICV33 -2.833e-01 4.814e-01 1.470e+02 -0.589

# IC_VICV34 -4.471e-02 4.906e-01 1.470e+02 -0.091

# IC_VICV36 -2.575e-01 2.867e-01 1.470e+02 -0.898

# IC_VICV37 -3.014e-01 2.873e-01 1.470e+02 -1.049

# IC_VICV39 1.324e-01 1.970e-01 1.470e+02 0.672

# IC_VICV40 -2.522e-02 2.085e-01 1.470e+02 -0.121

# IC_VICV43 -5.284e-01 4.928e-01 1.470e+02 -1.072

# IC_VICV44 -4.287e-01 4.901e-01 1.470e+02 -0.875

# IC_VICV45 -3.700e-01 4.887e-01 1.470e+02 -0.757

# IC_VICV46 5.000e-02 4.887e-01 1.470e+02 0.102

# IC_VICV47 -4.352e-01 4.915e-01 1.470e+02 -0.885

# IC_VICV48 3.815e-01 4.979e-01 1.470e+02 0.766

# IC_VICV49 -3.000e-02 4.887e-01 1.470e+02 -0.061

# IC_VICV5 -5.700e-01 5.676e-01 1.470e+02 -1.004

# IC_VICV50 4.686e-01 4.957e-01 1.470e+02 0.945

# IC_VICV51 -4.290e-01 4.901e-01 1.470e+02 -0.875

# IC_VICV52 4.000e-02 4.887e-01 1.470e+02 0.082

# IC_VICV53 1.284e-01 4.901e-01 1.470e+02 0.262

# IC_VICV54 1.500e-02 4.887e-01 1.470e+02 0.031

# IC_VICV55 -1.246e-15 4.887e-01 1.470e+02 0.000

# IC_VICV56 2.187e-01 4.901e-01 1.470e+02 0.446

# IC_VICV8 -1.130e+00 6.135e-01 1.470e+02 -1.842

# IC_VICV9 -1.304e-01 1.627e-01 1.470e+02 -0.802

# Pr(>|t|)

# (Intercept) 1.00000

# HC_VHCV21 0.75044

# HC_VHCV22 1.00000

# HC_VHCV23 1.00000

# HC_VHCV25 1.00000

# HC_VHCV27 1.00000

# HC_VHCV28 1.00000

# HC_VHCV29 1.00000

# HC_VHCV30 1.00000

# HC_VHCV31 1.00000

# HC_VHCV32 1.00000

# HC_VHCV33 1.00000

# HC_VHCV37 1.00000

# HC_VHCV38 1.00000

# HC_VHCV51 1.00000

# HC_VHCV57 1.00000

# HC_VHCV58 1.00000

# HC_VHCV59 1.00000

# HC_VHCV60 1.00000

# HC_VHCV61 1.00000

# HC_VHCV62 1.00000

# HC_VHCV7 1.00000

# IC_VICV11 0.66911

# IC_VICV12 0.38326

# IC_VICV13 0.66438

# IC_VICV14 0.84019

# IC_VICV15 0.90788

# IC_VICV17 0.00645 **

# IC_VICV18 0.94872

# IC_VICV19 0.42290

# IC_VICV22 0.19649

# IC_VICV25 1.00000

# IC_VICV26 1.00000

# IC_VICV27 0.64604

# IC_VICV30 0.34828

# IC_VICV31 0.63751

# IC_VICV32 0.96751

# IC_VICV33 0.55708

# IC_VICV34 0.92751

# IC_VICV36 0.37070

# IC_VICV37 0.29579

# IC_VICV39 0.50268

# IC_VICV40 0.90387

# IC_VICV43 0.28539

# IC_VICV44 0.38316

# IC_VICV45 0.45023

# IC_VICV46 0.91866

# IC_VICV47 0.37734

# IC_VICV48 0.44473

# IC_VICV49 0.95114

# IC_VICV5 0.31694

# IC_VICV50 0.34603

# IC_VICV51 0.38290

# IC_VICV52 0.93488

# IC_VICV53 0.79373

# IC_VICV54 0.97556

# IC_VICV55 1.00000

# IC_VICV56 0.65606

# IC_VICV8 0.06743 .

# IC_VICV9 0.42412

# ---

# Signif. codes:

# 0 ‘***’ 0.001 ‘**’ 0.01 ‘*’ 0.05 ‘.’ 0.1 ‘ ’ 1

# Correlation matrix not shown by default, as p = 60 > 12.

# Use print(x, correlation=TRUE) or

# vcov(x) if you need it

# fit warnings:

# fixed-effect model matrix is rank deficient so dropping 14 columns / coefficients

# Model 3

Type III Analysis of Variance Table with Satterthwaite's method

Sum Sq Mean Sq NumDF DenDF F value Pr(>F)

W_SP 46.064 9.2128 5 36.016 2.8511 0.02867 *

HC_SP 3.751 1.2503 3 43.060 0.3869 0.76295

IC_SP 132.412 5.7571 23 65.137 1.7817 0.03610 *

---

Signif. codes:

0 ‘***’ 0.001 ‘**’ 0.01 ‘*’ 0.05 ‘.’ 0.1 ‘ ’ 1

> summary(mixed.mod1)

Linear mixed model fit by REML. t-tests use

Satterthwaite's method [lmerModLmerTest]

Formula:

HEDGES ~ W_SP + HC_SP + IC_SP + (1 | Study_ID)

Data: MST_IC_ASD_IMP_YD

Weights: 1/VAR_G

REML criterion at convergence: 1149.7

Scaled residuals:

Min 1Q Median 3Q Max

-3.7725 -0.5209 0.0000 0.3313 3.3046

Random effects:

Groups Name Variance Std.Dev.

Study_ID (Intercept) 0.3315 0.5758

Residual 3.2313 1.7976

Number of obs: 395, groups: Study_ID, 27

Fixed effects:

Estimate

(Intercept) -2.83000

W_SPO. crenata -427.40000

W_SPO. foetida -168.88341

W_SPO.crenata -167.68390

W_SPS.asiatica 2.24566

W_SPS.hermonthica 2.51662

HC_SPFinger millet -0.11733

HC_SPMaize 0.30817

HC_SPPearl millet 0.42127

IC_SPBerseem 170.19080

IC_SPCelery 96.89000

IC_SPCelosia argentia -0.02842

IC_SPCommon bean -0.41496

IC_SPCowpea -0.10737

IC_SPCrotalaria ochroleuca -0.51685

IC_SPD.intortum -1.36929

IC_SPD.uncinatum -1.74834

IC_SPD.uncinatum, D.intortum -0.94843

IC_SPDesmodium spp -3.95161

IC_SPDesmodium spp / Common bean -4.45390

IC_SPFaba beans -0.12947

IC_SPFaidherbia albida -0.71662

IC_SPFenugreek 169.94567

IC_SPFlax 243.69000

IC_SPGarlic 260.73000

IC_SPGroundnut -0.19961

IC_SPLupin 170.10890

IC_SPMung bean -0.29984

IC_SPPigeon pea 0.48433

IC_SPSesbania sesban 0.48322

IC_SPSoya bean -0.28510

IC_SPSweet potao 0.91513

Std. Error

(Intercept) 1.64024

W_SPO. crenata 223.26140

W_SPO. foetida 242.98717

W_SPO.crenata 242.98633

W_SPS.asiatica 1.77738

W_SPS.hermonthica 1.72975

HC_SPFinger millet 1.01959

HC_SPMaize 0.30886

HC_SPPearl millet 0.91573

IC_SPBerseem 242.98026

IC_SPCelery 282.42556

IC_SPCelosia argentia 0.77579

IC_SPCommon bean 0.54583

IC_SPCowpea 0.48241

IC_SPCrotalaria ochroleuca 0.55254

IC_SPD.intortum 0.56260

IC_SPD.uncinatum 0.67551

IC_SPD.uncinatum, D.intortum 0.78310

IC_SPDesmodium spp 1.65831

IC_SPDesmodium spp / Common bean 1.81149

IC_SPFaba beans 0.61329

IC_SPFaidherbia albida 1.17319

IC_SPFenugreek 242.97997

IC_SPFlax 243.33867

IC_SPGarlic 239.95994

IC_SPGroundnut 0.49123

IC_SPLupin 242.98088

IC_SPMung bean 0.54838

IC_SPPigeon pea 0.86377

IC_SPSesbania sesban 0.86524

IC_SPSoya bean 0.56360

IC_SPSweet potao 0.93044

df t value

(Intercept) 222.69075 -1.725

W_SPO. crenata 348.76370 -1.914

W_SPO. foetida 348.76162 -0.695

W_SPO.crenata 348.76162 -0.690

W_SPS.asiatica 155.85911 1.263

W_SPS.hermonthica 218.55287 1.455

HC_SPFinger millet 43.74385 -0.115

HC_SPMaize 84.25764 0.998

HC_SPPearl millet 29.01202 0.460

IC_SPBerseem 348.74933 0.700

IC_SPCelery 348.74876 0.343

IC_SPCelosia argentia 274.73720 -0.037

IC_SPCommon bean 355.25035 -0.760

IC_SPCowpea 351.79864 -0.223

IC_SPCrotalaria ochroleuca 359.41314 -0.935

IC_SPD.intortum 353.24381 -2.434

IC_SPD.uncinatum 28.01361 -2.588

IC_SPD.uncinatum, D.intortum 15.95393 -1.211

IC_SPDesmodium spp 335.74196 -2.383

IC_SPDesmodium spp / Common bean 348.21738 -2.459

IC_SPFaba beans 353.33693 -0.211

IC_SPFaidherbia albida 72.33491 -0.611

IC_SPFenugreek 348.74933 0.699

IC_SPFlax 348.74898 1.001

IC_SPGarlic 348.74900 1.087

IC_SPGroundnut 362.14946 -0.406

IC_SPLupin 348.74933 0.700

IC_SPMung bean 358.89696 -0.547

IC_SPPigeon pea 22.73423 0.561

IC_SPSesbania sesban 22.88741 0.558

IC_SPSoya bean 259.29413 -0.506

IC_SPSweet potao 286.31969 0.984

Pr(>|t|)

(Intercept) 0.0858 .

W_SPO. crenata 0.0564 .

W_SPO. foetida 0.4875

W_SPO.crenata 0.4906

W_SPS.asiatica 0.2083

W_SPS.hermonthica 0.1471

HC_SPFinger millet 0.9089

HC_SPMaize 0.3212

HC_SPPearl millet 0.6489

IC_SPBerseem 0.4841

IC_SPCelery 0.7318

IC_SPCelosia argentia 0.9708

IC_SPCommon bean 0.4476

IC_SPCowpea 0.8240

IC_SPCrotalaria ochroleuca 0.3502

IC_SPD.intortum 0.0154 *

IC_SPD.uncinatum 0.0151 *

IC_SPD.uncinatum, D.intortum 0.2435

IC_SPDesmodium spp 0.0177 *

IC_SPDesmodium spp / Common bean 0.0144 *

IC_SPFaba beans 0.8329

IC_SPFaidherbia albida 0.5432

IC_SPFenugreek 0.4848

IC_SPFlax 0.3173

IC_SPGarlic 0.2780

IC_SPGroundnut 0.6847

IC_SPLupin 0.4843

IC_SPMung bean 0.5849

IC_SPPigeon pea 0.5805

IC_SPSesbania sesban 0.5819

IC_SPSoya bean 0.6134

IC_SPSweet potao 0.3262

---

Signif. codes:

0 ‘***’ 0.001 ‘**’ 0.01 ‘*’ 0.05 ‘.’ 0.1 ‘ ’ 1

# Model 4

Type III Analysis of Variance Table with Satterthwaite's method

Sum Sq Mean Sq NumDF DenDF F value Pr(>F)

HC_V 4.5258 0.50286 9 103 1.2481 0.2745

IC_V 7.7694 0.40892 19 103 1.0149 0.4510

> summary(mixed.mod4)

Linear mixed model fit by REML. t-tests use

Satterthwaite's method [lmerModLmerTest]

Formula: HEDGES ~ HC_V + IC_V + (1 | Study_ID)

Data: MST_IC_ASD_IMP_YD

Weights: 1/VAR_G

REML criterion at convergence: 134.3

Scaled residuals:

Min 1Q Median 3Q Max

-2.7376 -0.5631 0.0000 0.5934 3.0778

Random effects:

Groups Name Variance Std.Dev.

Study_ID (Intercept) 2.7248 1.6507

Residual 0.4029 0.6348

Number of obs: 132, groups: Study_ID, 11

Fixed effects:

Estimate Std. Error df t value

(Intercept) -0.471106 1.662691 103.000000 -0.283

HC_VHCV23 1.548276 2.354740 103.000000 0.658

HC_VHCV27 0.063940 2.345029 103.000000 0.027

HC_VHCV28 -0.343205 2.352100 103.000000 -0.146

HC_VHCV29 0.814799 0.256907 103.000000 3.172

HC_VHCV33 0.211106 2.362625 103.000000 0.089

HC_VHCV37 -0.334006 2.361734 103.000000 -0.141

HC_VHCV38 0.111820 2.348097 103.000000 0.048

HC_VHCV57 0.148497 2.352780 103.000000 0.063

HC_VHCV7 0.018248 2.348200 103.000000 0.008

IC_VICV11 -0.007929 0.140982 103.000000 -0.056

IC_VICV12 -0.022837 0.140611 103.000000 -0.162

IC_VICV13 0.217576 0.289754 103.000000 0.751

IC_VICV14 0.461108 0.286491 103.000000 1.610

IC_VICV15 0.224215 0.288967 103.000000 0.776

IC_VICV17 -0.197938 0.228476 103.000000 -0.866

IC_VICV21 -0.117143 2.346598 103.000000 -0.050

IC_VICV22 -0.880000 0.444328 103.000000 -1.981

IC_VICV25 0.542857 2.353456 103.000000 0.231

IC_VICV26 0.190915 2.353676 103.000000 0.081

IC_VICV27 0.223571 0.180209 103.000000 1.241

IC_VICV30 -1.064805 0.456421 103.000000 -2.333

IC_VICV31 -0.139487 0.419624 103.000000 -0.332

IC_VICV32 -0.323350 0.433175 103.000000 -0.746

IC_VICV33 0.070111 0.413593 103.000000 0.170

IC_VICV34 -0.774110 0.461886 103.000000 -1.676

IC_VICV36 -0.082391 0.304274 103.000000 -0.271

IC_VICV37 -0.246115 0.305889 103.000000 -0.805

IC_VICV9 -0.032393 0.141237 103.000000 -0.229

Pr(>|t|)

(Intercept) 0.7775

HC_VHCV23 0.5123

HC_VHCV27 0.9783

HC_VHCV28 0.8843

HC_VHCV29 0.0020 **

HC_VHCV33 0.9290

HC_VHCV37 0.8878

HC_VHCV38 0.9621

HC_VHCV57 0.9498

HC_VHCV7 0.9938

IC_VICV11 0.9553

IC_VICV12 0.8713

IC_VICV13 0.4544

IC_VICV14 0.1106

IC_VICV15 0.4396

IC_VICV17 0.3883

IC_VICV21 0.9603

IC_VICV22 0.0503 .

IC_VICV25 0.8180

IC_VICV26 0.9355

IC_VICV27 0.2176

IC_VICV30 0.0216 *

IC_VICV31 0.7403

IC_VICV32 0.4571

IC_VICV33 0.8657

IC_VICV34 0.0968 .

IC_VICV36 0.7871

IC_VICV37 0.4229

IC_VICV9 0.8191

---

Signif. codes:

0 ‘***’ 0.001 ‘**’ 0.01 ‘*’ 0.05 ‘.’ 0.1 ‘ ’ 1

# Model 5

Type III Analysis of Variance Table with Satterthwaite's method

Sum Sq Mean Sq NumDF DenDF F value Pr(>F)

W_SP 60.026 7.5033 8 10.205 2.1522 0.1255

HC_SP 47.349 6.7642 7 15.278 1.9402 0.1320

RC_1_SP 316.112 3.9026 81 217.374 1.1194 0.2596

> summary(mixed.mod5)

Linear mixed model fit by REML. t-tests use

Satterthwaite's method [lmerModLmerTest]

Formula:

HEDGES ~ W_SP + HC_SP + RC_1_SP + (1 | Study_ID)

Data: MST_RC_ASD_IMP_WD

Weights: 1/VAR_G

REML criterion at convergence: 1008.2

Scaled residuals:

Min 1Q Median 3Q Max

-2.02677 -0.32372 0.00039 0.54804 2.74769

Random effects:

Groups Name Variance Std.Dev.

Study_ID (Intercept) 0.878 0.937

Residual 3.486 1.867

Number of obs: 368, groups: Study_ID, 29

Fixed effects:

Estimate

(Intercept) 2.48651

W_SPO.aegyptiaca 1.52543

W_SPO.cernua -2.60847

W_SPO.crenata -1.69762

W_SPO.cumana -0.07121

W_SPO.minor -2.11985

W_SPO.ramosa -2.43402

W_SPPhelipanche aegyptiaca -2.41952

W_SPS.hermonthica -3.42183

HC_SPLentil 0.40682

HC_SPMaize 0.93894

HC_SPPearl millet 0.96890

HC_SPRapeseed -3.48480

HC_SPSorghum 0.21947

HC_SPSorghum / Millet 5.36019

HC_SPSorhgum/Maize 1.27443

RC_1_SPAniseed 0.46001

RC_1_SPBarley 0.90233

RC_1_SPBasil 0.36111

RC_1_SPBeet 0.78693

RC_1_SPBerseem 0.06580

RC_1_SPBitter apple 1.52240

RC_1_SPBlack-eyed pea 9.25947

RC_1_SPBroccoli 1.10197

RC_1_SPBrown Indian Hemp -0.48053

RC_1_SPBrussel sprout -1.52005

RC_1_SPButternut squash 0.53970

RC_1_SPCabbage -1.38784

RC_1_SPCanola -1.59871

RC_1_SPCauliflower -0.75023

RC_1_SPCereal 0.86854

RC_1_SPChickpea 1.68419

RC_1_SPChilli 1.39620

RC_1_SPCommon bean 1.43351

RC_1_SPCommon vetch -4.33194

RC_1_SPCoriander 0.67266

RC_1_SPCotton 1.63835

RC_1_SPCowpea 0.83909

RC_1_SPCrotalaria grahamiana 0.53214

RC_1_SPCrotalaria juncea 0.74005

RC_1_SPCucumber 0.37989

RC_1_SPCucumis prophetarum 0.97365

RC_1_SPCumin 1.03207

RC_1_SPD. distortum 6.12405

RC_1_SPDill 0.30043

RC_1_SPEndive 0.62263

RC_1_SPFallow -0.35920

RC_1_SPFenugreek 1.25592

RC_1_SPFlax 1.08544

RC_1_SPFoxtail millet -0.78804

RC_1_SPGarden pea 0.35459

RC_1_SPGarlic 0.72979

RC_1_SPGiant spinach 0.72633

RC_1_SPGourd 0.76710

RC_1_SPGroundnut 0.67885

RC_1_SPLentil 1.06837

RC_1_SPLinseed 0.12242

RC_1_SPLupin 1.21901

RC_1_SPMaize 0.52520

RC_1_SPMaize/Cowpea/ Soya bean/ Groundnut -0.09221

RC_1_SPMelon 0.35637

RC_1_SPMillet / Cotton 0.68585

RC_1_SPMung bean 1.04092

RC_1_SPMustard 1.36606

RC_1_SPNarbon vetch 1.92111

RC_1_SPOnion 0.63923

RC_1_SPParsley 0.84228

RC_1_SPPepper 1.22106

RC_1_SPPigeon pea 1.01946

RC_1_SPProso millet 0.11289

RC_1_SPRed cabbage 0.63769

RC_1_SPRoselle 1.51455

RC_1_SPSenna didymobotrya 1.67405

RC_1_SPSenna occidentalis -0.20595

RC_1_SPSenna spectabilis -0.14595

RC_1_SPSesame 0.73732

RC_1_SPSesbania cinerascens 2.12405

RC_1_SPSesbania sesban -0.16134

RC_1_SPSilverleaf nightshade -0.51547

RC_1_SPSnap bean 1.39874

RC_1_SPSorhgum -0.81175

RC_1_SPSoya bean 0.75089

RC_1_SPSpinach 0.18280

RC_1_SPSquash 0.50402

RC_1_SPSquirting cucumber 0.97300

RC_1_SPSugar beet 0.50710

RC_1_SPSunflower 0.87298

RC_1_SPSyrian oregano -0.17583

RC_1_SPTephrosia vogelii 1.59405

RC_1_SPTithonia diversifolia 0.17405

RC_1_SPTriticale 0.47333

RC_1_SPTurnip -0.67396

RC_1_SPVigna mungo 1.36196

RC_1_SPWatermelon 0.08604

RC_1_SPWheat 1.58301

RC_1_SPWild rue 0.71510

RC_1_SPWinter durum wheat 0.81333

Std. Error

(Intercept) 1.23381

W_SPO.aegyptiaca 2.08377

W_SPO.cernua 1.57759

W_SPO.crenata 1.53674

W_SPO.cumana 1.40217

W_SPO.minor 1.60092

W_SPO.ramosa 1.48282

W_SPPhelipanche aegyptiaca 1.39199

W_SPS.hermonthica 1.57040

HC_SPLentil 1.58412

HC_SPMaize 1.08907

HC_SPPearl millet 1.62047

HC_SPRapeseed 2.11632

HC_SPSorghum 2.04908

HC_SPSorghum / Millet 1.84461

HC_SPSorhgum/Maize 1.61868

RC_1_SPAniseed 0.80611

RC_1_SPBarley 0.83398

RC_1_SPBasil 1.17446

RC_1_SPBeet 0.92969

RC_1_SPBerseem 0.77365

RC_1_SPBitter apple 0.87794

RC_1_SPBlack-eyed pea 5.07723

RC_1_SPBroccoli 0.89287

RC_1_SPBrown Indian Hemp 1.58866

RC_1_SPBrussel sprout 1.46208

RC_1_SPButternut squash 0.80366

RC_1_SPCabbage 1.35975

RC_1_SPCanola 1.35959

RC_1_SPCauliflower 1.34294

RC_1_SPCereal 0.84025

RC_1_SPChickpea 0.88676

RC_1_SPChilli 0.85469

RC_1_SPCommon bean 0.77158

RC_1_SPCommon vetch 1.88027

RC_1_SPCoriander 0.79603

RC_1_SPCotton 1.45938

RC_1_SPCowpea 0.73082

RC_1_SPCrotalaria grahamiana 1.29798

RC_1_SPCrotalaria juncea 1.04896

RC_1_SPCucumber 0.80366

RC_1_SPCucumis prophetarum 0.92813

RC_1_SPCumin 0.79706

RC_1_SPD. distortum 3.17101

RC_1_SPDill 0.79947

RC_1_SPEndive 0.94030

RC_1_SPFallow 0.97120

RC_1_SPFenugreek 0.81859

RC_1_SPFlax 0.77656

RC_1_SPFoxtail millet 1.46652

RC_1_SPGarden pea 0.83752

RC_1_SPGarlic 1.47720

RC_1_SPGiant spinach 0.94036

RC_1_SPGourd 0.90409

RC_1_SPGroundnut 0.71635

RC_1_SPLentil 1.08521

RC_1_SPLinseed 1.36006

RC_1_SPLupin 0.86586

RC_1_SPMaize 0.80935

RC_1_SPMaize/Cowpea/ Soya bean/ Groundnut 1.22711

RC_1_SPMelon 0.80163

RC_1_SPMillet / Cotton 1.61799

RC_1_SPMung bean 0.92589

RC_1_SPMustard 0.87426

RC_1_SPNarbon vetch 1.40429

RC_1_SPOnion 1.49957

RC_1_SPParsley 0.84582

RC_1_SPPepper 0.83658

RC_1_SPPigeon pea 1.18191

RC_1_SPProso millet 0.90244

RC_1_SPRed cabbage 0.91246

RC_1_SPRoselle 0.93013

RC_1_SPSenna didymobotrya 1.66393

RC_1_SPSenna occidentalis 1.51016

RC_1_SPSenna spectabilis 1.49857

RC_1_SPSesame 0.71278

RC_1_SPSesbania cinerascens 1.76559

RC_1_SPSesbania sesban 0.99122

RC_1_SPSilverleaf nightshade 0.79947

RC_1_SPSnap bean 1.63615

RC_1_SPSorhgum 0.71910

RC_1_SPSoya bean 0.69482

RC_1_SPSpinach 0.79475

RC_1_SPSquash 0.80366

RC_1_SPSquirting cucumber 0.90665

RC_1_SPSugar beet 1.17597

RC_1_SPSunflower 0.85755

RC_1_SPSyrian oregano 0.86260

RC_1_SPTephrosia vogelii 1.64285

RC_1_SPTithonia diversifolia 1.49857

RC_1_SPTriticale 1.27554

RC_1_SPTurnip 1.33174

RC_1_SPVigna mungo 1.52626

RC_1_SPWatermelon 0.79475

RC_1_SPWheat 1.31001

RC_1_SPWild rue 0.93818

RC_1_SPWinter durum wheat 1.32908

df

(Intercept) 14.55899

W_SPO.aegyptiaca 29.32604

W_SPO.cernua 9.64984

W_SPO.crenata 8.72065

W_SPO.cumana 12.13011

W_SPO.minor 10.31467

W_SPO.ramosa 7.55541

W_SPPhelipanche aegyptiaca 12.97707

W_SPS.hermonthica 9.43802

HC_SPLentil 9.90423

HC_SPMaize 7.28671

HC_SPPearl millet 10.80617

HC_SPRapeseed 31.26987

HC_SPSorghum 27.28503

HC_SPSorghum / Millet 18.14197

HC_SPSorhgum/Maize 10.78288

RC_1_SPAniseed 255.08801

RC_1_SPBarley 255.17294

RC_1_SPBasil 255.29737

RC_1_SPBeet 255.05135

RC_1_SPBerseem 255.47595

RC_1_SPBitter apple 255.06483

RC_1_SPBlack-eyed pea 261.07578

RC_1_SPBroccoli 255.17638

RC_1_SPBrown Indian Hemp 245.65854

RC_1_SPBrussel sprout 255.24464

RC_1_SPButternut squash 255.08891

RC_1_SPCabbage 255.29198

RC_1_SPCanola 255.29207

RC_1_SPCauliflower 255.30081

RC_1_SPCereal 261.58135

RC_1_SPChickpea 255.16472

RC_1_SPChilli 255.07170

RC_1_SPCommon bean 259.01981

RC_1_SPCommon vetch 255.32693

RC_1_SPCoriander 255.21323

RC_1_SPCotton 255.82922

RC_1_SPCowpea 265.26014

RC_1_SPCrotalaria grahamiana 124.62143

RC_1_SPCrotalaria juncea 258.06976

RC_1_SPCucumber 255.08891

RC_1_SPCucumis prophetarum 255.05172

RC_1_SPCumin 255.19570

RC_1_SPD. distortum 270.79746

RC_1_SPDill 255.09047

RC_1_SPEndive 255.04886

RC_1_SPFallow 60.10614

RC_1_SPFenugreek 254.96212

RC_1_SPFlax 259.85286

RC_1_SPFoxtail millet 14.30638

RC_1_SPGarden pea 255.41651

RC_1_SPGarlic 255.70657

RC_1_SPGiant spinach 255.04884

RC_1_SPGourd 255.05773

RC_1_SPGroundnut 263.66942

RC_1_SPLentil 255.40935

RC_1_SPLinseed 255.84266

RC_1_SPLupin 255.06833

RC_1_SPMaize 189.50262

RC_1_SPMaize/Cowpea/ Soya bean/ Groundnut 259.29785

RC_1_SPMelon 255.08966

RC_1_SPMillet / Cotton 255.66501

RC_1_SPMung bean 258.85231

RC_1_SPMustard 255.06588

RC_1_SPNarbon vetch 255.19034

RC_1_SPOnion 255.68409

RC_1_SPParsley 255.07447

RC_1_SPPepper 268.40685

RC_1_SPPigeon pea 246.85780

RC_1_SPProso millet 255.05816

RC_1_SPRed cabbage 255.05558

RC_1_SPRoselle 255.05124

RC_1_SPSenna didymobotrya 206.08761

RC_1_SPSenna occidentalis 175.93830

RC_1_SPSenna spectabilis 173.37876

RC_1_SPSesame 256.79261

RC_1_SPSesbania cinerascens 221.90112

RC_1_SPSesbania sesban 56.21417

RC_1_SPSilverleaf nightshade 255.09047

RC_1_SPSnap bean 255.56603

RC_1_SPSorhgum 261.76591

RC_1_SPSoya bean 264.74336

RC_1_SPSpinach 255.09226

RC_1_SPSquash 255.08891

RC_1_SPSquirting cucumber 255.05707

RC_1_SPSugar beet 230.51333

RC_1_SPSunflower 255.43527

RC_1_SPSyrian oregano 255.06930

RC_1_SPTephrosia vogelii 202.39461

RC_1_SPTithonia diversifolia 173.37876

RC_1_SPTriticale 254.94009

RC_1_SPTurnip 255.30687

RC_1_SPVigna mungo 256.45344

RC_1_SPWatermelon 255.09226

RC_1_SPWheat 250.59574

RC_1_SPWild rue 255.04935

RC_1_SPWinter durum wheat 254.94009

t value

(Intercept) 2.015

W_SPO.aegyptiaca 0.732

W_SPO.cernua -1.653

W_SPO.crenata -1.105

W_SPO.cumana -0.051

W_SPO.minor -1.324

W_SPO.ramosa -1.641

W_SPPhelipanche aegyptiaca -1.738

W_SPS.hermonthica -2.179

HC_SPLentil 0.257

HC_SPMaize 0.862

HC_SPPearl millet 0.598

HC_SPRapeseed -1.647

HC_SPSorghum 0.107

HC_SPSorghum / Millet 2.906

HC_SPSorhgum/Maize 0.787

RC_1_SPAniseed 0.571

RC_1_SPBarley 1.082

RC_1_SPBasil 0.307

RC_1_SPBeet 0.846

RC_1_SPBerseem 0.085

RC_1_SPBitter apple 1.734

RC_1_SPBlack-eyed pea 1.824

RC_1_SPBroccoli 1.234

RC_1_SPBrown Indian Hemp -0.302

RC_1_SPBrussel sprout -1.040

RC_1_SPButternut squash 0.672

RC_1_SPCabbage -1.021

RC_1_SPCanola -1.176

RC_1_SPCauliflower -0.559

RC_1_SPCereal 1.034

RC_1_SPChickpea 1.899

RC_1_SPChilli 1.634

RC_1_SPCommon bean 1.858

RC_1_SPCommon vetch -2.304

RC_1_SPCoriander 0.845

RC_1_SPCotton 1.123

RC_1_SPCowpea 1.148

RC_1_SPCrotalaria grahamiana 0.410

RC_1_SPCrotalaria juncea 0.706

RC_1_SPCucumber 0.473

RC_1_SPCucumis prophetarum 1.049

RC_1_SPCumin 1.295

RC_1_SPD. distortum 1.931

RC_1_SPDill 0.376

RC_1_SPEndive 0.662

RC_1_SPFallow -0.370

RC_1_SPFenugreek 1.534

RC_1_SPFlax 1.398

RC_1_SPFoxtail millet -0.537

RC_1_SPGarden pea 0.423

RC_1_SPGarlic 0.494

RC_1_SPGiant spinach 0.772

RC_1_SPGourd 0.848

RC_1_SPGroundnut 0.948

RC_1_SPLentil 0.984

RC_1_SPLinseed 0.090

RC_1_SPLupin 1.408

RC_1_SPMaize 0.649

RC_1_SPMaize/Cowpea/ Soya bean/ Groundnut -0.075

RC_1_SPMelon 0.445

RC_1_SPMillet / Cotton 0.424

RC_1_SPMung bean 1.124

RC_1_SPMustard 1.563

RC_1_SPNarbon vetch 1.368

RC_1_SPOnion 0.426

RC_1_SPParsley 0.996

RC_1_SPPepper 1.460

RC_1_SPPigeon pea 0.863

RC_1_SPProso millet 0.125

RC_1_SPRed cabbage 0.699

RC_1_SPRoselle 1.628

RC_1_SPSenna didymobotrya 1.006

RC_1_SPSenna occidentalis -0.136

RC_1_SPSenna spectabilis -0.097

RC_1_SPSesame 1.034

RC_1_SPSesbania cinerascens 1.203

RC_1_SPSesbania sesban -0.163

RC_1_SPSilverleaf nightshade -0.645

RC_1_SPSnap bean 0.855

RC_1_SPSorhgum -1.129

RC_1_SPSoya bean 1.081

RC_1_SPSpinach 0.230

RC_1_SPSquash 0.627

RC_1_SPSquirting cucumber 1.073

RC_1_SPSugar beet 0.431

RC_1_SPSunflower 1.018

RC_1_SPSyrian oregano -0.204

RC_1_SPTephrosia vogelii 0.970

RC_1_SPTithonia diversifolia 0.116

RC_1_SPTriticale 0.371

RC_1_SPTurnip -0.506

RC_1_SPVigna mungo 0.892

RC_1_SPWatermelon 0.108

RC_1_SPWheat 1.208

RC_1_SPWild rue 0.762

RC_1_SPWinter durum wheat 0.612

Pr(>|t|)

(Intercept) 0.06272 .

W_SPO.aegyptiaca 0.46995

W_SPO.cernua 0.13035

W_SPO.crenata 0.29884

W_SPO.cumana 0.96033

W_SPO.minor 0.21405

W_SPO.ramosa 0.14154

W_SPPhelipanche aegyptiaca 0.10583

W_SPS.hermonthica 0.05591 .

HC_SPLentil 0.80258

HC_SPMaize 0.41607

HC_SPPearl millet 0.56222

HC_SPRapeseed 0.10965

HC_SPSorghum 0.91549

HC_SPSorghum / Millet 0.00937 **

HC_SPSorhgum/Maize 0.44805

RC_1_SPAniseed 0.56874

RC_1_SPBarley 0.28030

RC_1_SPBasil 0.75874

RC_1_SPBeet 0.39810

RC_1_SPBerseem 0.93229

RC_1_SPBitter apple 0.08412 .

RC_1_SPBlack-eyed pea 0.06934 .

RC_1_SPBroccoli 0.21827

RC_1_SPBrown Indian Hemp 0.76255

RC_1_SPBrussel sprout 0.29949

RC_1_SPButternut squash 0.50248

RC_1_SPCabbage 0.30838

RC_1_SPCanola 0.24074

RC_1_SPCauliflower 0.57689

RC_1_SPCereal 0.30225

RC_1_SPChickpea 0.05866 .

RC_1_SPChilli 0.10358

RC_1_SPCommon bean 0.06432 .

RC_1_SPCommon vetch 0.02203 *

RC_1_SPCoriander 0.39889

RC_1_SPCotton 0.26264

RC_1_SPCowpea 0.25194

RC_1_SPCrotalaria grahamiana 0.68253

RC_1_SPCrotalaria juncea 0.48113

RC_1_SPCucumber 0.63683

RC_1_SPCucumis prophetarum 0.29515

RC_1_SPCumin 0.19654

RC_1_SPD. distortum 0.05449 .

RC_1_SPDill 0.70739

RC_1_SPEndive 0.50847

RC_1_SPFallow 0.71279

RC_1_SPFenugreek 0.12621

RC_1_SPFlax 0.16338

RC_1_SPFoxtail millet 0.59928

RC_1_SPGarden pea 0.67238

RC_1_SPGarlic 0.62170

RC_1_SPGiant spinach 0.44060

RC_1_SPGourd 0.39697

RC_1_SPGroundnut 0.34418

RC_1_SPLentil 0.32581

RC_1_SPLinseed 0.92835

RC_1_SPLupin 0.16039

RC_1_SPMaize 0.51718

RC_1_SPMaize/Cowpea/ Soya bean/ Groundnut 0.94016

RC_1_SPMelon 0.65702

RC_1_SPMillet / Cotton 0.67200

RC_1_SPMung bean 0.26195

RC_1_SPMustard 0.11940

RC_1_SPNarbon vetch 0.17251

RC_1_SPOnion 0.67027

RC_1_SPParsley 0.32028

RC_1_SPPepper 0.14557

RC_1_SPPigeon pea 0.38922

RC_1_SPProso millet 0.90054

RC_1_SPRed cabbage 0.48528

RC_1_SPRoselle 0.10469

RC_1_SPSenna didymobotrya 0.31556

RC_1_SPSenna occidentalis 0.89168

RC_1_SPSenna spectabilis 0.92252

RC_1_SPSesame 0.30191

RC_1_SPSesbania cinerascens 0.23025

RC_1_SPSesbania sesban 0.87128

RC_1_SPSilverleaf nightshade 0.51966

RC_1_SPSnap bean 0.39341

RC_1_SPSorhgum 0.26000

RC_1_SPSoya bean 0.28082

RC_1_SPSpinach 0.81827

RC_1_SPSquash 0.53112

RC_1_SPSquirting cucumber 0.28420

RC_1_SPSugar beet 0.66671

RC_1_SPSunflower 0.30964

RC_1_SPSyrian oregano 0.83865

RC_1_SPTephrosia vogelii 0.33306

RC_1_SPTithonia diversifolia 0.90767

RC_1_SPTriticale 0.71088

RC_1_SPTurnip 0.61324

RC_1_SPVigna mungo 0.37304

RC_1_SPWatermelon 0.91388

RC_1_SPWheat 0.22804

RC_1_SPWild rue 0.44663

RC_1_SPWinter durum wheat 0.54111

# Model 6

Type III Analysis of Variance Table with Satterthwaite's method

Sum Sq Mean Sq NumDF DenDF F value Pr(>F)

HC_V 173.32 11.5544 15 43 1.9530 0.04392 *

RC_1_V 122.55 2.9178 42 43 0.4932 0.98819

---

Signif. codes:

0 ‘***’ 0.001 ‘**’ 0.01 ‘*’ 0.05 ‘.’ 0.1 ‘ ’ 1

> summary(mixed.mod6)

Linear mixed model fit by REML. t-tests use

Satterthwaite's method [lmerModLmerTest]

Formula: HEDGES ~ HC_V + RC_1_V + (1 | Study_ID)

Data: MST_RC_ASD_IMP_WD

Weights: 1/VAR_G

REML criterion at convergence: 203.5

Scaled residuals:

Min 1Q Median 3Q Max

-1.3160 -0.1490 0.0000 0.3867 2.2090

Random effects:

Groups Name Variance Std.Dev.

Study_ID (Intercept) 0.000 0.000

Residual 5.916 2.432

Number of obs: 101, groups: Study_ID, 16

Fixed effects:

Estimate Std. Error df

(Intercept) 4.68023 1.99089 43.00000

HC_VHCV2 -3.49171 1.35255 43.00000

HC_VHCV20 -5.29538 2.10980 43.00000

HC_VHCV21 -5.47083 2.10980 43.00000

HC_VHCV4 -4.43023 2.12040 43.00000

HC_VHCV5 -3.30023 2.48045 43.00000

HC_VHCV6 -4.42023 2.12040 43.00000

HC_VHCV61 -0.01023 3.31723 43.00000

HC_VHCV64 -2.98661 2.30260 43.00000

HC_VHCV66 -3.50023 2.58556 43.00000

HC_VHCV67 -2.54183 2.45118 43.00000

HC_VHCV7 -3.39523 2.77325 43.00000

HC_VHCV71 5.66977 6.76696 43.00000

HC_VHCV73 -4.71023 2.08523 43.00000

HC_VHCV8 -3.90140 2.19832 43.00000

HC_VHCV9 -2.09340 2.13392 43.00000

RC_1_VRCV11 -1.26500 2.16191 43.00000

RC_1_VRCV12 -1.25500 1.66753 43.00000

RC_1_VRCV12/RCV58 -0.91500 2.06391 43.00000

RC_1_VRCV13 -0.92000 2.09238 43.00000

RC_1_VRCV14 -0.93000 2.09238 43.00000

RC_1_VRCV15 -0.69000 2.09238 43.00000

RC_1_VRCV16 -0.94000 2.09238 43.00000

RC_1_VRCV17 -0.73000 2.09238 43.00000

RC_1_VRCV18 -0.67000 2.09238 43.00000

RC_1_VRCV19 -1.61890 2.18078 43.00000

RC_1_VRCV2 -4.99000 2.95907 43.00000

RC_1_VRCV20 -1.04071 2.20429 43.00000

RC_1_VRCV21 -0.72047 2.20006 43.00000

RC_1_VRCV22 -0.03072 2.27951 43.00000

RC_1_VRCV23 -1.45246 2.10618 43.00000

RC_1_VRCV24 -0.32471 2.22650 43.00000

RC_1_VRCV25 -0.08592 2.26855 43.00000

RC_1_VRCV27 -0.38227 1.33581 43.00000

RC_1_VRCV28 0.34930 1.38751 43.00000

RC_1_VRCV29 -2.46274 1.96033 43.00000

RC_1_VRCV30 -0.11856 1.98065 43.00000

RC_1_VRCV31 -1.69292 1.80992 43.00000

RC_1_VRCV32 -2.54140 1.83089 43.00000

RC_1_VRCV33 -2.33053 1.83109 43.00000

RC_1_VRCV34 -1.61665 1.79582 43.00000

RC_1_VRCV37 -0.42000 1.71993 43.00000

RC_1_VRCV39 -0.88500 2.02045 43.00000

RC_1_VRCV4 -0.34000 2.28174 43.00000

RC_1_VRCV40 -1.30000 1.97604 43.00000

RC_1_VRCV41 0.50595 1.32285 43.00000

RC_1_VRCV42 -0.10370 1.31824 43.00000

RC_1_VRCV48 -1.06499 1.04173 43.00000

RC_1_VRCV51 -5.94000 7.15787 43.00000

RC_1_VRCV52 -8.87000 6.70109 43.00000

RC_1_VRCV53 -9.74000 6.64791 43.00000

RC_1_VRCV54 -2.44000 8.19811 43.00000

RC_1_VRCV55 0.19000 9.22688 43.00000

RC_1_VRCV57 -0.88500 2.13437 43.00000

RC_1_VRCV59 0.95250 0.91010 43.00000

RC_1_VRCV6 -0.18620 1.60174 43.00000

RC_1_VRCV7 0.42150 1.62435 43.00000

RC_1_VRCV8 2.07648 1.89511 43.00000

t value Pr(>|t|)

(Intercept) 2.351 0.0234 *

HC_VHCV2 -2.582 0.0133 *

HC_VHCV20 -2.510 0.0159 *

HC_VHCV21 -2.593 0.0129 *

HC_VHCV4 -2.089 0.0426 *

HC_VHCV5 -1.330 0.1904

HC_VHCV6 -2.085 0.0431 *

HC_VHCV61 -0.003 0.9976

HC_VHCV64 -1.297 0.2015

HC_VHCV66 -1.354 0.1829

HC_VHCV67 -1.037 0.3055

HC_VHCV7 -1.224 0.2275

HC_VHCV71 0.838 0.4067

HC_VHCV73 -2.259 0.0290 *

HC_VHCV8 -1.775 0.0830 .

HC_VHCV9 -0.981 0.3321

RC_1_VRCV11 -0.585 0.5615

RC_1_VRCV12 -0.753 0.4558

RC_1_VRCV12/RCV58 -0.443 0.6597

RC_1_VRCV13 -0.440 0.6624

RC_1_VRCV14 -0.444 0.6589

RC_1_VRCV15 -0.330 0.7432

RC_1_VRCV16 -0.449 0.6555

RC_1_VRCV17 -0.349 0.7289

RC_1_VRCV18 -0.320 0.7504

RC_1_VRCV19 -0.742 0.4619

RC_1_VRCV2 -1.686 0.0990 .

RC_1_VRCV20 -0.472 0.6392

RC_1_VRCV21 -0.327 0.7449

RC_1_VRCV22 -0.013 0.9893

RC_1_VRCV23 -0.690 0.4941

RC_1_VRCV24 -0.146 0.8847

RC_1_VRCV25 -0.038 0.9700

RC_1_VRCV27 -0.286 0.7761

RC_1_VRCV28 0.252 0.8024

RC_1_VRCV29 -1.256 0.2158

RC_1_VRCV30 -0.060 0.9525

RC_1_VRCV31 -0.935 0.3548

RC_1_VRCV32 -1.388 0.1723

RC_1_VRCV33 -1.273 0.2099

RC_1_VRCV34 -0.900 0.3730

RC_1_VRCV37 -0.244 0.8082

RC_1_VRCV39 -0.438 0.6636

RC_1_VRCV4 -0.149 0.8822

RC_1_VRCV40 -0.658 0.5141

RC_1_VRCV41 0.382 0.7040

RC_1_VRCV42 -0.079 0.9377

RC_1_VRCV48 -1.022 0.3123

RC_1_VRCV51 -0.830 0.4112

RC_1_VRCV52 -1.324 0.1926

RC_1_VRCV53 -1.465 0.1502

RC_1_VRCV54 -0.298 0.7674

RC_1_VRCV55 0.021 0.9837

RC_1_VRCV57 -0.415 0.6805

RC_1_VRCV59 1.047 0.3011

RC_1_VRCV6 -0.116 0.9080

RC_1_VRCV7 0.259 0.7965

RC_1_VRCV8 1.096 0.2793

# Model 7

Type III Analysis of Variance Table with Satterthwaite's method

Sum Sq Mean Sq NumDF DenDF F value Pr(>F)

DIV 0.062847 0.062847 1 181.92 0.017 0.8965

> summary(mixed.mod7)

Linear mixed model fit by REML. t-tests use

Satterthwaite's method [lmerModLmerTest]

Formula: HEDGES ~ DIV + (1 | Study_ID)

Data: MST_RC_ASD_IMP_WD

Weights: 1/VAR_G

REML criterion at convergence: 1263

Scaled residuals:

Min 1Q Median 3Q Max

-3.3575 -0.4104 0.0825 0.7565 3.1825

Random effects:

Groups Name Variance Std.Dev.

Study_ID (Intercept) 0.8663 0.9307

Residual 3.7039 1.9246

Number of obs: 368, groups: Study_ID, 29

Fixed effects:

Estimate Std. Error df t value

(Intercept) 1.01486 0.66485 144.65689 1.526

DIV -0.04031 0.30945 181.92414 -0.130

Pr(>|t|)

(Intercept) 0.129

DIV 0.897

Correlation of Fixed Effects:

(Intr)

DIV -0.957

# Model 8

Type III Analysis of Variance Table with Satterthwaite's method

Sum Sq Mean Sq NumDF DenDF F value Pr(>F)

W_SP 22.392 3.7320 6 69 0.9968 0.4346

HC_SP 1.420 0.4735 3 69 0.1265 0.9441

RC_1_SP 110.846 2.1317 52 69 0.5694 0.9824

> summary(mixed.mod5)

Linear mixed model fit by REML. t-tests use

Satterthwaite's method [lmerModLmerTest]

Formula:

HEDGES ~ W_SP + HC_SP + RC_1_SP + (1 | Study_ID)

Data: MST_RC_ASD_IMP_YD

Weights: 1/VAR_G

REML criterion at convergence: 249.7

Scaled residuals:

Min 1Q Median 3Q Max

-2.2813 -0.1420 0.0000 0.1533 2.1696

Random effects:

Groups Name Variance Std.Dev.

Study_ID (Intercept) 0.000 0.000

Residual 3.744 1.935

Number of obs: 131, groups: Study_ID, 18

Fixed effects:

Estimate

(Intercept) -0.73000

W_SPO.aegyptiaca 0.64108

W_SPO.cernua 0.57829

W_SPO.crenata -0.22143

W_SPO.ramosa 0.72935

W_SPPhelipanche aegyptiaca -1.90950

W_SPS.hermonthica 1.44046

HC_SPMaize -0.67773

HC_SPPea -0.41639

HC_SPPearl millet 0.02066

RC_1_SPAubergine -0.02435

RC_1_SPBarley 1.41782

RC_1_SPBasil 0.61782

RC_1_SPBerseem 0.96476

RC_1_SPBlack-eyed pea -12.88050

RC_1_SPBroccoli 1.09143

RC_1_SPBrown Indian Hemp -17.09050

RC_1_SPBrussel sprout 1.23555

RC_1_SPCabbage 1.20338

RC_1_SPCanola 1.30210

RC_1_SPCauliflower 1.26324

RC_1_SPChickpea 1.13782

RC_1_SPCommon bean 0.41881

RC_1_SPCommon vetch 0.34892

RC_1_SPCoriander 0.84782

RC_1_SPCotton -1.28046

RC_1_SPCowpea -0.22484

RC_1_SPCrotalaria grahamiana -0.62891

RC_1_SPCrotalaria juncea -0.48829

RC_1_SPCumin 0.83782

RC_1_SPD. distortum -3.43273

RC_1_SPFaba bean 0.03782

RC_1_SPFallow -0.07770

RC_1_SPFenugreek -0.60000

RC_1_SPFlax 0.09927

RC_1_SPGarden pea -0.10463

RC_1_SPGarlic -0.16025

RC_1_SPGroundnut -0.17735

RC_1_SPLentil 0.76861

RC_1_SPLinseed -0.12000

RC_1_SPMaize -0.16624

RC_1_SPMaize/Cowpea/ Soya bean/ Groundnut 0.27727

RC_1_SPMung bean 0.07902

RC_1_SPNarbon vetch 0.70782

RC_1_SPOnion -0.11000

RC_1_SPPepper -0.19980

RC_1_SPPigeon pea -0.86533

RC_1_SPSenna didymobotrya -1.52273

RC_1_SPSenna occidentalis 0.77727

RC_1_SPSenna spectabilis -1.67773

RC_1_SPSesame -0.84112

RC_1_SPSesbania cinerascens -2.28273

RC_1_SPSesbania sesban -1.20603

RC_1_SPSmooth vetch 0.55782

RC_1_SPSnap bean -0.86000

RC_1_SPSorhgum -0.03784

RC_1_SPSoya bean -0.29305

RC_1_SPSunflower -0.19829

RC_1_SPTephrosia vogelii -3.05273

RC_1_SPTithonia diversifolia -4.89273

RC_1_SPTomato 0.14576

RC_1_SPTurnip 1.31196

Std. Error

(Intercept) 1.32651

W_SPO.aegyptiaca 1.46762

W_SPO.cernua 1.46591

W_SPO.crenata 1.42555

W_SPO.ramosa 0.92888

W_SPPhelipanche aegyptiaca 1.64740

W_SPS.hermonthica 1.60523

HC_SPMaize 2.19122

HC_SPPea 0.84991

HC_SPPearl millet 2.13434

RC_1_SPAubergine 1.81558

RC_1_SPBarley 2.01521

RC_1_SPBasil 2.03370

RC_1_SPBerseem 1.93124

RC_1_SPBlack-eyed pea 7.89172

RC_1_SPBroccoli 2.12207

RC_1_SPBrown Indian Hemp 9.84620

RC_1_SPBrussel sprout 2.14056

RC_1_SPCabbage 2.12424

RC_1_SPCanola 2.14924

RC_1_SPCauliflower 2.13615

RC_1_SPChickpea 2.01521

RC_1_SPCommon bean 1.81565

RC_1_SPCommon vetch 2.23590

RC_1_SPCoriander 2.02448

RC_1_SPCotton 2.00920

RC_1_SPCowpea 2.02883

RC_1_SPCrotalaria grahamiana 2.14667

RC_1_SPCrotalaria juncea 2.32510

RC_1_SPCumin 2.02448

RC_1_SPD. distortum 2.85334

RC_1_SPFaba bean 2.06113

RC_1_SPFallow 2.10388

RC_1_SPFenugreek 1.97323

RC_1_SPFlax 1.84918

RC_1_SPGarden pea 1.97068

RC_1_SPGarlic 1.64025

RC_1_SPGroundnut 2.02191

RC_1_SPLentil 1.93862

RC_1_SPLinseed 1.89582

RC_1_SPMaize 1.99909

RC_1_SPMaize/Cowpea/ Soya bean/ Groundnut 2.20157

RC_1_SPMung bean 1.95049

RC_1_SPNarbon vetch 2.02448

RC_1_SPOnion 1.89582

RC_1_SPPepper 1.66494

RC_1_SPPigeon pea 2.06578

RC_1_SPSenna didymobotrya 2.44337

RC_1_SPSenna occidentalis 2.36552

RC_1_SPSenna spectabilis 2.24785

RC_1_SPSesame 1.68603

RC_1_SPSesbania cinerascens 2.57032

RC_1_SPSesbania sesban 2.16264

RC_1_SPSmooth vetch 2.03370

RC_1_SPSnap bean 2.03856

RC_1_SPSorhgum 2.03008

RC_1_SPSoya bean 2.01644

RC_1_SPSunflower 2.31703

RC_1_SPTephrosia vogelii 2.75317

RC_1_SPTithonia diversifolia 3.31465

RC_1_SPTomato 1.85075

RC_1_SPTurnip 2.15843

df

(Intercept) 69.00000

W_SPO.aegyptiaca 69.00000

W_SPO.cernua 69.00000

W_SPO.crenata 69.00000

W_SPO.ramosa 69.00000

W_SPPhelipanche aegyptiaca 69.00000

W_SPS.hermonthica 69.00000

HC_SPMaize 69.00000

HC_SPPea 69.00000

HC_SPPearl millet 69.00000

RC_1_SPAubergine 69.00000

RC_1_SPBarley 69.00000

RC_1_SPBasil 69.00000

RC_1_SPBerseem 69.00000

RC_1_SPBlack-eyed pea 69.00000

RC_1_SPBroccoli 69.00000

RC_1_SPBrown Indian Hemp 69.00000

RC_1_SPBrussel sprout 69.00000

RC_1_SPCabbage 69.00000

RC_1_SPCanola 69.00000

RC_1_SPCauliflower 69.00000

RC_1_SPChickpea 69.00000

RC_1_SPCommon bean 69.00000

RC_1_SPCommon vetch 69.00000

RC_1_SPCoriander 69.00000

RC_1_SPCotton 69.00000

RC_1_SPCowpea 69.00000

RC_1_SPCrotalaria grahamiana 69.00000

RC_1_SPCrotalaria juncea 69.00000

RC_1_SPCumin 69.00000

RC_1_SPD. distortum 69.00000

RC_1_SPFaba bean 69.00000

RC_1_SPFallow 69.00000

RC_1_SPFenugreek 69.00000

RC_1_SPFlax 69.00000

RC_1_SPGarden pea 69.00000

RC_1_SPGarlic 69.00000

RC_1_SPGroundnut 69.00000

RC_1_SPLentil 69.00000

RC_1_SPLinseed 69.00000

RC_1_SPMaize 69.00000

RC_1_SPMaize/Cowpea/ Soya bean/ Groundnut 69.00000

RC_1_SPMung bean 69.00000

RC_1_SPNarbon vetch 69.00000

RC_1_SPOnion 69.00000

RC_1_SPPepper 69.00000

RC_1_SPPigeon pea 69.00000

RC_1_SPSenna didymobotrya 69.00000

RC_1_SPSenna occidentalis 69.00000

RC_1_SPSenna spectabilis 69.00000

RC_1_SPSesame 69.00000

RC_1_SPSesbania cinerascens 69.00000

RC_1_SPSesbania sesban 69.00000

RC_1_SPSmooth vetch 69.00000

RC_1_SPSnap bean 69.00000

RC_1_SPSorhgum 69.00000

RC_1_SPSoya bean 69.00000

RC_1_SPSunflower 69.00000

RC_1_SPTephrosia vogelii 69.00000

RC_1_SPTithonia diversifolia 69.00000

RC_1_SPTomato 69.00000

RC_1_SPTurnip 69.00000

t value

(Intercept) -0.550

W_SPO.aegyptiaca 0.437

W_SPO.cernua 0.394

W_SPO.crenata -0.155

W_SPO.ramosa 0.785

W_SPPhelipanche aegyptiaca -1.159

W_SPS.hermonthica 0.897

HC_SPMaize -0.309

HC_SPPea -0.490

HC_SPPearl millet 0.010

RC_1_SPAubergine -0.013

RC_1_SPBarley 0.704

RC_1_SPBasil 0.304

RC_1_SPBerseem 0.500

RC_1_SPBlack-eyed pea -1.632

RC_1_SPBroccoli 0.514

RC_1_SPBrown Indian Hemp -1.736

RC_1_SPBrussel sprout 0.577

RC_1_SPCabbage 0.566

RC_1_SPCanola 0.606

RC_1_SPCauliflower 0.591

RC_1_SPChickpea 0.565

RC_1_SPCommon bean 0.231

RC_1_SPCommon vetch 0.156

RC_1_SPCoriander 0.419

RC_1_SPCotton -0.637

RC_1_SPCowpea -0.111

RC_1_SPCrotalaria grahamiana -0.293

RC_1_SPCrotalaria juncea -0.210

RC_1_SPCumin 0.414

RC_1_SPD. distortum -1.203

RC_1_SPFaba bean 0.018

RC_1_SPFallow -0.037

RC_1_SPFenugreek -0.304

RC_1_SPFlax 0.054

RC_1_SPGarden pea -0.053

RC_1_SPGarlic -0.098

RC_1_SPGroundnut -0.088

RC_1_SPLentil 0.396

RC_1_SPLinseed -0.063

RC_1_SPMaize -0.083

RC_1_SPMaize/Cowpea/ Soya bean/ Groundnut 0.126

RC_1_SPMung bean 0.041

RC_1_SPNarbon vetch 0.350

RC_1_SPOnion -0.058

RC_1_SPPepper -0.120

RC_1_SPPigeon pea -0.419

RC_1_SPSenna didymobotrya -0.623

RC_1_SPSenna occidentalis 0.329

RC_1_SPSenna spectabilis -0.746

RC_1_SPSesame -0.499

RC_1_SPSesbania cinerascens -0.888

RC_1_SPSesbania sesban -0.558

RC_1_SPSmooth vetch 0.274

RC_1_SPSnap bean -0.422

RC_1_SPSorhgum -0.019

RC_1_SPSoya bean -0.145

RC_1_SPSunflower -0.086

RC_1_SPTephrosia vogelii -1.109

RC_1_SPTithonia diversifolia -1.476

RC_1_SPTomato 0.079

RC_1_SPTurnip 0.608

Pr(>|t|)

(Intercept) 0.5839

W_SPO.aegyptiaca 0.6636

W_SPO.cernua 0.6944

W_SPO.crenata 0.8770

W_SPO.ramosa 0.4350

W_SPPhelipanche aegyptiaca 0.2504

W_SPS.hermonthica 0.3727

HC_SPMaize 0.7580

HC_SPPea 0.6257

HC_SPPearl millet 0.9923

RC_1_SPAubergine 0.9893

RC_1_SPBarley 0.4841

RC_1_SPBasil 0.7622

RC_1_SPBerseem 0.6190

RC_1_SPBlack-eyed pea 0.1072

RC_1_SPBroccoli 0.6087

RC_1_SPBrown Indian Hemp 0.0871 .

RC_1_SPBrussel sprout 0.5657

RC_1_SPCabbage 0.5729

RC_1_SPCanola 0.5466

RC_1_SPCauliflower 0.5562

RC_1_SPChickpea 0.5742

RC_1_SPCommon bean 0.8183

RC_1_SPCommon vetch 0.8764

RC_1_SPCoriander 0.6767

RC_1_SPCotton 0.5260

RC_1_SPCowpea 0.9121

RC_1_SPCrotalaria grahamiana 0.7704

RC_1_SPCrotalaria juncea 0.8343

RC_1_SPCumin 0.6803

RC_1_SPD. distortum 0.2331

RC_1_SPFaba bean 0.9854

RC_1_SPFallow 0.9706

RC_1_SPFenugreek 0.7620

RC_1_SPFlax 0.9573

RC_1_SPGarden pea 0.9578

RC_1_SPGarlic 0.9225

RC_1_SPGroundnut 0.9304

RC_1_SPLentil 0.6930

RC_1_SPLinseed 0.9497

RC_1_SPMaize 0.9340

RC_1_SPMaize/Cowpea/ Soya bean/ Groundnut 0.9001

RC_1_SPMung bean 0.9678

RC_1_SPNarbon vetch 0.7277

RC_1_SPOnion 0.9539

RC_1_SPPepper 0.9048

RC_1_SPPigeon pea 0.6766

RC_1_SPSenna didymobotrya 0.5352

RC_1_SPSenna occidentalis 0.7435

RC_1_SPSenna spectabilis 0.4580

RC_1_SPSesame 0.6195

RC_1_SPSesbania cinerascens 0.3776

RC_1_SPSesbania sesban 0.5789

RC_1_SPSmooth vetch 0.7847

RC_1_SPSnap bean 0.6744

RC_1_SPSorhgum 0.9852

RC_1_SPSoya bean 0.8849

RC_1_SPSunflower 0.9320

RC_1_SPTephrosia vogelii 0.2714

RC_1_SPTithonia diversifolia 0.1445

RC_1_SPTomato 0.9375

RC_1_SPTurnip 0.5453

---

Signif. codes:

0 ‘***’ 0.001 ‘**’ 0.01 ‘*’ 0.05 ‘.’ 0.1 ‘ ’ 1

Correlation matrix not shown by default, as p = 62 > 12.

Use print(x, correlation=TRUE) or

vcov(x) if you need it

fit warnings:

fixed-effect model matrix is rank deficient so dropping 3 columns / coefficients

optimizer (nloptwrap) convergence code: 0 (OK)

boundary (singular) fit: see ?isSingular

# Model 9

Type III Analysis of Variance Table with Satterthwaite's method

Sum Sq Mean Sq NumDF DenDF F value Pr(>F)

DIV 1.3665 1.3665 1 125.4 0.4513 0.503

> summary(mixed.mod9)

Linear mixed model fit by REML. t-tests use

Satterthwaite's method [lmerModLmerTest]

Formula: HEDGES ~ DIV + (1 | Study_ID)

Data: MST_RC_ASD_IMP_YD

Weights: 1/VAR_G

REML criterion at convergence: 399.2

Scaled residuals:

Min 1Q Median 3Q Max

-2.3063 -0.6924 0.0000 0.3082 2.5576

Random effects:

Groups Name Variance Std.Dev.

Study_ID (Intercept) 0.06568 0.2563

Residual 3.02806 1.7401

Number of obs: 131, groups: Study_ID, 18

Fixed effects:

Estimate Std. Error df t value

(Intercept) -0.9285 0.8711 121.3603 -1.066

DIV 0.2866 0.4266 125.3965 0.672

Pr(>|t|)

(Intercept) 0.289

DIV 0.503

Correlation of Fixed Effects:

(Intr)

DIV -0.992

# Model 10

Type III Analysis of Variance Table with Satterthwaite's method

Sum Sq Mean Sq NumDF DenDF F value Pr(>F)

HC_V 20.481 2.9259 7 8 1.5439 0.2772

RC_1_V 18.622 1.0345 18 8 0.5459 0.8637

> summary(mixed.mod10)

Linear mixed model fit by REML. t-tests use

Satterthwaite's method [lmerModLmerTest]

Formula: HEDGES ~ HC_V + RC_1_V + (1 | Study_ID)

Data: MST_RC_ASD_IMP_YD

Weights: 1/VAR_G

REML criterion at convergence: 25.6

Scaled residuals:

Min 1Q Median 3Q Max

-1.069 0.000 0.000 0.000 1.253

Random effects:

Groups Name Variance Std.Dev.

Study_ID (Intercept) 0.000 0.000

Residual 1.895 1.377

Number of obs: 34, groups: Study_ID, 8

Fixed effects:

Estimate Std. Error df t value

(Intercept) -0.14000 0.41299 8.00000 -0.339

HC_VHCV5 0.42000 0.85970 8.00000 0.489

HC_VHCV6 0.03000 0.58405 8.00000 0.051

HC_VHCV61 0.83000 0.85970 8.00000 0.965

HC_VHCV67 -0.08792 0.73165 8.00000 -0.120

HC_VHCV7 0.51000 1.10987 8.00000 0.460

HC_VHCV71 -15.38000 5.42503 8.00000 -2.835

HC_VHCV73 -0.43000 0.56760 8.00000 -0.758

RC_1_VRCV11 -0.43000 1.16811 8.00000 -0.368

RC_1_VRCV12 -0.49000 0.87066 8.00000 -0.563

RC_1_VRCV2 -0.43000 1.05741 8.00000 -0.407

RC_1_VRCV29 0.51204 0.87448 8.00000 0.586

RC_1_VRCV30 0.36792 0.85136 8.00000 0.432

RC_1_VRCV31 0.53973 0.86901 8.00000 0.621

RC_1_VRCV32 0.57859 0.88519 8.00000 0.654

RC_1_VRCV33 0.47987 0.85410 8.00000 0.562

RC_1_VRCV34 0.58845 0.89644 8.00000 0.656

RC_1_VRCV37 -0.32000 0.90271 8.00000 -0.354

RC_1_VRCV39 -0.46000 1.08396 8.00000 -0.424

RC_1_VRCV40 -1.18000 1.09266 8.00000 -1.080

RC_1_VRCV50 1.74000 7.24399 8.00000 0.240

RC_1_VRCV51 0.82000 7.45542 8.00000 0.110

RC_1_VRCV52 -2.40000 8.24598 8.00000 -0.291

RC_1_VRCV53 -4.21000 8.72178 8.00000 -0.483

RC_1_VRCV54 -0.73000 7.82501 8.00000 -0.093

RC_1_VRCV55 8.63000 5.96733 8.00000 1.446

Pr(>|t|)

(Intercept) 0.743

HC_VHCV5 0.638

HC_VHCV6 0.960

HC_VHCV61 0.363

HC_VHCV67 0.907

HC_VHCV7 0.658

HC_VHCV71 0.022 *

HC_VHCV73 0.470

RC_1_VRCV11 0.722

RC_1_VRCV12 0.589

RC_1_VRCV2 0.695

RC_1_VRCV29 0.574

RC_1_VRCV30 0.677

RC_1_VRCV31 0.552

RC_1_VRCV32 0.532

RC_1_VRCV33 0.590

RC_1_VRCV34 0.530

RC_1_VRCV37 0.732

RC_1_VRCV39 0.682

RC_1_VRCV40 0.312

RC_1_VRCV50 0.816

RC_1_VRCV51 0.915

RC_1_VRCV52 0.778

RC_1_VRCV53 0.642

RC_1_VRCV54 0.928

RC_1_VRCV55 0.186

anova(mixed.mod9)

Type III Analysis of Variance Table with Satterthwaite's method

Sum Sq Mean Sq NumDF DenDF F value

Treat_Mean 1166825 1166825 1 596.9 930.34

Pr(>F)

Treat_Mean < 2.2e-16 ***

---

Signif. codes:

0 ‘***’ 0.001 ‘**’ 0.01 ‘*’ 0.05 ‘.’ 0.1 ‘ ’ 1

> summary(mixed.mod9)

Linear mixed model fit by REML. t-tests use

Satterthwaite's method [lmerModLmerTest]

Formula: Control_Mean ~ Treat_Mean + (1 | Study_ID)

Data: MST_IC_ASD_IMP_WD

Weights: 1/VAR_G

REML criterion at convergence: 5627.2

Scaled residuals:

Min 1Q Median 3Q Max

-6.6268 -0.2529 -0.0215 0.0664 7.7265

Random effects:

Groups Name Variance Std.Dev.

Study_ID (Intercept) 3051 55.23

Residual 1254 35.41

Number of obs: 622, groups: Study_ID, 39

Fixed effects:

Estimate Std. Error df t value

(Intercept) 23.84318 9.00931 37.46104 2.647

Treat_Mean 1.04242 0.03418 596.89695 30.501

Pr(>|t|)

(Intercept) 0.0118 *

Treat_Mean <2e-16 ***

---

Signif. codes:

0 ‘***’ 0.001 ‘**’ 0.01 ‘*’ 0.05 ‘.’ 0.1 ‘ ’ 1

Correlation of Fixed Effects:

(Intr)

Treat_Mean -0.073

>

> MST_IC_ASD_IMP_YD<-read.csv("MST_IC_ASD_IMP_YD.CSV")

>

> mixed.mod10 <- lmer(Control_Mean ~

+ Treat_Mean +

+ (1|Study_ID),

+ data=MST_IC_ASD_IMP_YD,weights = 1/VAR_G, # this is the weighting variable required for a meta-analysis (var.g = the individual effect size variances)

+ na.action = "na.omit")

> anova(mixed.mod10)

Type III Analysis of Variance Table with Satterthwaite's method

Sum Sq Mean Sq NumDF DenDF F value

Treat_Mean 963.9 963.9 1 393 595.07

Pr(>F)

Treat_Mean < 2.2e-16 ***

---

Signif. codes:

0 ‘***’ 0.001 ‘**’ 0.01 ‘*’ 0.05 ‘.’ 0.1 ‘ ’ 1

> summary(mixed.mod10)

Linear mixed model fit by REML. t-tests use

Satterthwaite's method [lmerModLmerTest]

Formula: Control_Mean ~ Treat_Mean + (1 | Study_ID)

Data: MST_IC_ASD_IMP_YD

Weights: 1/VAR_G

REML criterion at convergence: 987.2

Scaled residuals:

Min 1Q Median 3Q Max

-3.9861 -0.5073 -0.0818 0.4075 5.5256

Random effects:

Groups Name Variance Std.Dev.

Study_ID (Intercept) 0.5758 0.7588

Residual 1.6198 1.2727

Number of obs: 395, groups: Study_ID, 27

Fixed effects:

Estimate Std. Error df t value

(Intercept) 0.18753 0.17696 37.59851 1.06

Treat_Mean 0.66807 0.02739 392.99942 24.39

Pr(>|t|)

(Intercept) 0.296

Treat_Mean <2e-16 ***

---

Signif. codes:

0 ‘***’ 0.001 ‘**’ 0.01 ‘*’ 0.05 ‘.’ 0.1 ‘ ’ 1

Correlation of Fixed Effects:

(Intr)

Treat_Mean -0.388

>

> MST_RC_ASD_IMP_WD<-read.csv("MST_RC_ASD_IMP_WD.CSV")

>

> mixed.mod11 <- lmer(Control_Mean ~

+ Treat_Mean +

+ (1|Study_ID),

+ data=MST_RC_ASD_IMP_WD,weights = 1/VAR_G, # this is the weighting variable required for a meta-analysis (var.g = the individual effect size variances)

+ na.action = "na.omit")

> anova(mixed.mod11)

Type III Analysis of Variance Table with Satterthwaite's method

Sum Sq Mean Sq NumDF DenDF F value

Treat_Mean 120950 120950 1 351.89 258.03

Pr(>F)

Treat_Mean < 2.2e-16 ***

---

Signif. codes:

0 ‘***’ 0.001 ‘**’ 0.01 ‘*’ 0.05 ‘.’ 0.1 ‘ ’ 1

> summary(mixed.mod11)

Linear mixed model fit by REML. t-tests use

Satterthwaite's method [lmerModLmerTest]

Formula: Control_Mean ~ Treat_Mean + (1 | Study_ID)

Data: MST_RC_ASD_IMP_WD

Weights: 1/VAR_G

REML criterion at convergence: 3112.3

Scaled residuals:

Min 1Q Median 3Q Max

-3.8640 -0.1812 -0.0270 0.4081 4.2404

Random effects:

Groups Name Variance Std.Dev.

Study_ID (Intercept) 1590.1 39.88

Residual 468.7 21.65

Number of obs: 368, groups: Study_ID, 29

Fixed effects:

Estimate Std. Error df t value

(Intercept) 26.39078 7.55346 27.48383 3.494

Treat_Mean 0.59327 0.03693 351.89019 16.063

Pr(>|t|)

(Intercept) 0.00163 **

Treat_Mean < 2e-16 ***

---

Signif. codes:

0 ‘***’ 0.001 ‘**’ 0.01 ‘*’ 0.05 ‘.’ 0.1 ‘ ’ 1

Correlation of Fixed Effects:

(Intr)

Treat_Mean -0.136

>

> MST_RC_ASD_IMP_YD<-read.csv("MST_RC_ASD_IMP_YD.CSV")

>

> mixed.mod12 <- lmer(Control_Mean ~

+ Treat_Mean +

+ (1|Study_ID),

+ data=MST_RC_ASD_IMP_YD,weights = 1/VAR_G, # this is the weighting variable required for a meta-analysis (var.g = the individual effect size variances)

+ na.action = "na.omit")

> anova(mixed.mod12)

Type III Analysis of Variance Table with Satterthwaite's method

Sum Sq Mean Sq NumDF DenDF F value

Treat_Mean 0.091297 0.091297 1 112.18 0.1276

Pr(>F)

Treat_Mean 0.7217

> summary(mixed.mod12)

Linear mixed model fit by REML. t-tests use

Satterthwaite's method [lmerModLmerTest]

Formula: Control_Mean ~ Treat_Mean + (1 | Study_ID)

Data: MST_RC_ASD_IMP_YD

Weights: 1/VAR_G

REML criterion at convergence: 347.3

Scaled residuals:

Min 1Q Median 3Q Max

-3.7912 -0.1588 -0.0031 0.0472 2.8222

Random effects:

Groups Name Variance Std.Dev.

Study_ID (Intercept) 103.1257 10.155

Residual 0.7157 0.846

Number of obs: 131, groups: Study_ID, 18

Fixed effects:

Estimate Std. Error df t value

(Intercept) 5.486e+00 2.395e+00 1.698e+01 2.291

Treat_Mean 1.540e-03 4.311e-03 1.122e+02 0.357

Pr(>|t|)

(Intercept) 0.035 *

Treat_Mean 0.722

---

Signif. codes:

0 ‘***’ 0.001 ‘**’ 0.01 ‘*’ 0.05 ‘.’ 0.1 ‘ ’ 1

Correlation of Fixed Effects:

(Intr)

Treat_Mean -0.016

>

> #Divide treatment by control to make weed density % difference

> MST_RC_ASD_IMP_WD$WDDif<- (MST_RC_ASD_IMP_WD$Treat_Mean/MST_RC_ASD_IMP_WD$Control_Mean)*100

>

> #Look at diversity and change in weed density

> mixed.mod13 <- lmer( WDDif ~ DIV + (1|Study_ID),data=MST_RC_ASD_IMP_WD,weights = 1/VAR_G, # this is the weighting variable required for a meta-analysis (var.g = the individual effect size variances)

+ na.action = "na.omit")

> anova(mixed.mod13)

Type III Analysis of Variance Table with Satterthwaite's method

Sum Sq Mean Sq NumDF DenDF F value Pr(>F)

DIV 19.524 19.524 1 110.7 1e-04 0.9906

> summary(mixed.mod13)

Linear mixed model fit by REML. t-tests use

Satterthwaite's method [lmerModLmerTest]

Formula: WDDif ~ DIV + (1 | Study_ID)

Data: MST_RC_ASD_IMP_WD

Weights: 1/VAR_G

REML criterion at convergence: 5096.5

Scaled residuals:

Min 1Q Median 3Q Max

-3.0913 -0.1607 -0.0795 0.0188 9.9005

Random effects:

Groups Name Variance Std.Dev.

Study_ID (Intercept) 8923 94.46

Residual 140155 374.37

Number of obs: 368, groups: Study_ID, 29

Fixed effects:

Estimate Std. Error df t value

(Intercept) 103.3261 104.0829 105.8979 0.993

DIV -0.5804 49.1776 110.7024 -0.012

Pr(>|t|)

(Intercept) 0.323

DIV 0.991

Correlation of Fixed Effects:

(Intr)

DIV -0.975

>
