# Supplementary material for: Crop diversification and parasitic weed abundance: a global meta-analysis
Source: Sci Rep. 2022 Nov 12;12:19413. doi: 10.1038/s41598-022-24047-2 (PMC9653488; doi:10.1038/s41598-022-24047-2)
Supplement: Supplementary file 5 — Supplementary Information 5. [file 41598_2022_24047_MOESM5_ESM.docx]

| **Term 1** | **Term 2** | **Term 3** | **Term 4** | **Term 5** | **Term 6** | **Term 7** | **# Refs gross** | | **# Refs net** | **Notes** |
| --- | --- | --- | --- | --- | --- | --- | --- | --- | --- | --- |
| Orobanche | AND | Cover | AND | Crop |  |  | 38 |  | 37 |  |
| Orobanche | AND | Intercrop |  |  |  |  | 20 |  | 18 |  |
| Orobanche | AND | Inter* |  |  |  |  | 737 |  | 461 | Too broad. Only first 500 exported |
| Orobanche | AND | Trap* |  |  |  |  | 66 |  | 47 |  |
| Orobanche | AND | Push | AND | Pull |  |  | 2 |  | 1 |  |
| Orobanche | AND | Companion |  |  |  |  | 3 |  | 1 |  |
| Orobanche | AND | Conservation | AND | Agriculture |  |  | 10 |  | 7 |  |
| Orobanche | AND | Integrated | AND | Weed | AND | Management | 1 |  | 1 |  |
| Orobanche | AND | Cultural | AND | Control |  |  | 103 |  | 61 | Perhaps too broad. Needs triage |
| Orobanche | AND | Suicid* |  |  |  |  | 40 |  | 4 | Too focused on biochemical study rather than actual intercrops. |
| Orobanche | AND | Legum* |  |  |  |  | 644 |  | 240 | Again could be too broad. Needs triage |
| Orobanche | AND | Legume |  |  |  |  | 256 |  |  |  |
| Orobanche | AND | no | AND | till |  |  | 3 |  | 2 |  |
| Orobanche | AND | zero | AND | till |  |  | 1 |  | 0 |  |
| Striga | AND | Cover | AND | Crop |  |  | 69 |  | 52 |  |
| Striga | AND | Intercrop |  |  |  |  | 129 |  | 103 |  |
| Striga | AND | Inter* |  |  |  |  | 921 |  | 195 | Too broad. Only first 500 exported |
| Striga | AND | Trap* |  |  |  |  | 109 |  | 27 |  |
| Striga | AND | Push | AND | Pull |  |  | 52 |  | 17 |  |
| Striga | AND | Companion |  |  |  |  | 16 |  | 6 |  |
| Striga | AND | Conservation | AND | Agriculture |  |  | 33 |  | 16 |  |
| Striga | AND | Integrated | AND | Weed | AND | Management | 154 |  | 87 |  |
| Striga | AND | Cultural | AND | Control |  |  | 165 |  | 52 |  |
| Striga | AND | Suicid* |  |  |  |  | 74 |  | 48 | Too focused on biochemical study rather than actual intercrops. |
| Striga | AND | Legum* |  |  |  |  | 572 |  | 282 | Again could be too broad. Needs triage |
| Striga | AND | Legume |  |  |  |  | 275 |  |  |  |
| Striga | AND | no | AND | till |  |  | 2 |  | 0 |  |
| Striga | AND | zero | AND | till |  |  | 1 |  | 0 |  |
|  |  |  |  |  |  | **Total Gross:**  4496 | | **Total Net:**  1765 | |  |

## Appendix 1A: Pilot Search Returns Table for Web of Science

## Appendix 1B: Taxa list for parasitic weeds of economic importance (with notes on biological characteristics)

| **Family** | **Synonym** | **Genus** | **Synonym** | **Sub genus** | **Synonym** | **Species** | **Included** | **Notes** |
| --- | --- | --- | --- | --- | --- | --- | --- | --- |
| Convolvulaceae | Cuscutaceae | Cuscuta |  | Monogynella |  | - | No | Robust vines which attack fruit trees. No information when searched. |
| Convolvulaceae |  | Cuscuta |  | Cuscuta |  | - | Yes | Favour herbaceous hosts. Holoparasites (photosynthetically inactive).  Just genus included in search as over 200 species listed with taxonomic  ambiguity. |
| Convolvulaceae |  | Cuscuta |  | Grammica |  | - | No | No evidence of economic significance |
| Lauraceae |  | Cassytha |  |  |  | - | No | Perennial / climbers affect woody plants |
| Orobanchaceae | Scrophulariaceae | Striga |  |  |  | S. asiatica | Yes |  |
| Orobanchaceae |  | Striga |  |  |  | S.hermonthica | Yes |  |
| Orobanchaceae |  | Striga |  |  |  | S. gesnerioides | Yes |  |
| Orobanchaceae |  | Striga |  |  |  | S. hirsuta | Yes | Less likely to attack crops but still cited as a threat |
| Orobanchaceae |  | Striga |  |  |  | S. lutea | Yes | Less likely to attack crops but still cited as a threat |
| Orobanchaceae |  | Striga |  |  |  | S. forbesii | Yes |  |
| Orobanchaceae |  | Striga |  |  |  | S. angustifolia | Yes |  |
| Orobanchaceae |  | Striga |  |  |  | S. densiflora | Yes |  |
| Orobanchaceae |  | Striga |  |  |  | S. aspera | Yes |  |
| Orobanchaceae |  | Striga |  |  |  | S. curviflora | Yes |  |
| Orobanchaceae |  | Striga |  |  |  | S. parviflora | Yes |  |
| Orobanchaceae |  | Striga |  |  |  | S. latericea | Yes |  |
| Orobanchaceae |  | Orobanche | Phelipanche |  |  | O. cernua | Yes | Over 70 species. Orobanche is a parasite of colder climates |
| Orobanchaceae |  | Orobanche |  |  |  | O. crenata | Yes |  |
| Orobanchaceae |  | Orobanche |  |  | O. cernua var. cumana | O. cumana | Yes |  |
| Orobanchaceae |  | Orobanche |  |  | Phelipanche ramosa | O. ramosa | Yes |  |
| Orobanchaceae |  | Orobanche |  |  | Phelipanche aegyptiaca | O. aegyptiaca | Yes |  |
| Orobanchaceae |  | Orobanche |  |  |  | O. foetida | Yes |  |
| Orobanchaceae |  | Aeginetia |  |  |  | A. indica | Yes | Only in forests in India but found affecting limited cereal crops |
| Orobanchaceae |  | Aeginetia |  |  |  | A. flava | No | Endemic to Thailand in rainforest |
| Orobanchaceae |  | Alectra |  |  |  | Alectra vogelii | Yes | Less significant but still reported as damaging crops |
| Orobanchaceae |  | Christisonia |  |  |  | C. tomentosa | No | Only recently described |
| Orobanchaceae |  | Christisonia |  |  |  | C. tubulosa | No | Obscure rare plant only found in India |
| Orobanchaceae |  | Christisonia |  |  |  | C. scortechinii | Yes | Limited range in Malaysia and Thailand. Affects sugarcane in Philippines |
| Orobanchaceae |  | Christisonia |  |  |  | Christisonia spp | No | Several others but no evidence for economic significance |
| Loranthaceae |  |  |  |  |  |  | No | Perennial / Affect canopies of woody species |
| Viscaceae |  |  |  |  |  |  | No | Mistletoes found in canopy of trees |

## Appendix 1B:

## Meta-analysis full methodology

## Pilot Study

A pilot study was undertaken using Web of Science and a combination of provisional terms to describe the use of companion crops, in conjunction with the genera: Striga and Orobanche (being among the most economically significant parasitic weed genera). Records returned for separate search term combinations were saved on EndNote Online (Clarivate Analytics 2021). Duplicate records were removed producing a net search term results total. The number of returns for each search combination, accompanied by an assessment of relevance based on the title of each study, gave an indication of the relevance of each search combination. This determined the final list of terms for inclusion; as some terms were too broad and returned too many unrelated results. Search combinations returning very high (e.g. >400) numbers of records with a very large proportion of non-relevant studies indicated that the term was too broad. These were subsequently omitted from the main search (e.g.: “Taxon” AND inter*, “Taxon” AND Legum*).

Choice of taxa for inclusion in the main search was determined by a number of criteria. Firstly, a review was undertaken to determine a definitive list of economically significant parasitic plants using several sources (Nickrent and Musselman 2004, Sauerborn et al 2007, Parker 2012). This list was then subject to triage, based on the nature of their parasitism, which determined inclusion in the main. For example, stem parasites such as mistletoes (Loranthaceae, Viscaceae) occur in the canopies of woody, perennial plants and will thus be unaffected by intercrops planted in the soil. Likewise, perennial, vine taxa affecting tree species such as the genus Cassytha were omitted for the same reason. Genera which returned no results for the 12 search combinations were removed from the main search. In the case of genera containing high numbers of economically-important species (e.g.: Cuscuta, Striga), genus was included as a search term alone without going to species level. Widely-adopted synonyms at the family and genus level were also included. Appendix 1 details search combinations used for pilot with gross and net results and list of taxa, synonyms and details of inclusion or omission from main search.

Main Search

Multiple electronic databases and the internet were searched using a range of Boolean search terms. The databases searched on the internet were: Web of Science, Scopus and AGRICOLA. Searches were performed in February 2021 on the complete range of references available at that time.

Search terms were constructed as follows: taxon name (Aeginetia, Alectra, Christisonia, Cuscuta, Grammica, Orobanche, Phelipanche, Scrophulariaceae, Striga) AND cover AND crop, taxon name AND Intercrop, taxon name AND trap*, taxon name AND push AND pull, taxon name AND companion, taxon name AND conservation AND agriculture *, taxon name AND integrated weed management, taxon name AND cultural AND control, taxon name AND suicidal*, taxon name AND legume, taxon name AND no AND till, taxon name AND zero AND till.

Additional searches were performed by manually searching for citations within relevant sections of 20 review studies of control methods for all economically-significant parasitic weed taxa. The list of reviews used is included in appendix 1. Recognized experts and practitioners in the field of parasitic weed agronomy were also contacted to identify possible sources of data (including primary data) and to verify the thoroughness of our literature coverage. In instances where studies were not available electronically, Jisc Libray Hub and Worldcat were searched to locate institutions holding hard copies, which were requested via inter library requests.

Criteria for Inclusion of Studies

Studies were included if they fulfilled the following relevance criteria:

Subjects studied: Any annual parasitic weed species, host crop and intercrop combinations

Treatment used: Intercropping or rotation cropping

Study type: Any primary studies with appropriate comparators, continuous data with means, information on sample sizes, available / calculable measures of variance or sufficient information to impute values. Range of studies comprised: Landscape-level assessment, laboratory, field trials, farm trials, pot, bag and rhizotron experiments.

Response(s): Host yield (t ha-1/kg ha-1), stover yield (t ha-1), weed dry weight (t ha-1/g pot/ g plant/ gm2), weed / weed seed density (per petri dish / pot / plant / M2/ log10M2 / density / severity score), percentage weed reduction / ratio ( versus control / from original density).

Comparator: Appropriate controls: experimental units in which no intercrop was grown with the host crop, or monocrop / fallow / bare earth in the case of rotation studies.
