# Supplementary material for: Crop diversification and parasitic weed abundance: a global meta-analysis
Source: Sci Rep. 2022 Nov 12;12:19413. doi: 10.1038/s41598-022-24047-2 (PMC9653488; doi:10.1038/s41598-022-24047-2)
Supplement: Supplementary file 6 — Supplementary Information 6. [file 41598_2022_24047_MOESM6_ESM.docx]

Appendix 3: Appendix 3, Meta-analysis locality, weed, host, inter and rotation crop lists

| **Countries** | **Localities** | **Lat** | **Lon** | **Weed Species** | **Host Crop** | **Inter/Trap crop** | **Host crop** | **Variety** | **Intercrop** | **Variety** | **Rotation Crop** | **Variety** |
| --- | --- | --- | --- | --- | --- | --- | --- | --- | --- | --- | --- | --- |
| Benin | Oued Beja, Tunisia | 36.7358 | 9.2249 | *Cuscuta chinensis* | Canola | Alfalfa | Canola | Zarfam | Barley | Aspen | Berseem | Tavor |
| Cameroon | Adana, Turkey | 37.0371 | 35.3551 | *Orobanche foetida* | Chickling pea | Aniseed | Chickling pea | BG-1043 | Berssem | Fahl | Black-eyed pea | Parastou |
| China | Adi Bakel, Tigray, Ethiopia | 13.9466 | 37.7973 | *Orobanche aegyptiaca* | Faba bean | Aubergine | Chickling pea | BGE-1023558 | Common bean | mwezi moja | Broccoli | Italica |
| Egypt | Alexandra, Egypt | 31.2037 | 30.0512 | *Orobanche cernua* | Finger millet | Bambara | Faba bean | Aquadolce | Common bean | Nambale | Broccoli | Monopoly-Syngenta |
| Ethiopia | Al-Jubeiha, Jordan | 32.0168 | 35.8716 | *Orobanche crenata* | Garden pea | Barley | Faba bean | Badi | Common bean | Nyayo | Brown indian hemp | Farakhil |
| Germany | Alkaleri, Nigeria | 9.7833 | 10.0166 | *Orobanche cumana* | Maize | Basil | Faba bean | Brocal | Cowpea | Amary-sho | Brussel sprout | Oliver-Syngenta |
| Ghana | Alupe, Kenya | 0.4833 | 34.1333 | *Orobanche minor* | Mung bean | Beet | Faba bean | Giza 429 | Cowpea | B301 | Cabbage | Brunswick-May |
| India | Amman, Jordan | 31.8622 | 35.9311 | *Orobanche ramosa* | Pea | Berseem | Faba bean | Giza Blanca | Cowpea | BR1 | Canola | 8310 |
| Iran | Ankwa, Nigeria | 9.9266 | 7.7666 | *Phelipanche aegyptiaca* | Pearl millet | Bitter apple | Faba bean | Najeh | Cowpea | ICV 2 | Cauliflower | Igloo-Global Seeds |
| Israel | Assiut University, Egypt | 27.1848 | 31.1641 | *Striga asiatica* | Rapeseed | Black-eyed pea | Faba bean | Prothabon | Cowpea | IT82D-849 | Common bean | GPL 94 |
| Jordan | Bauchi, northern Nigeria | 10.2847 | 9.8211 | *Striga hermonthica* | Red clover | Broccoli | Faba bean | Reina Blanca | Cowpea | IT90K-59 | Common vetch | Sadot |
| Kenya | Bengou, Niger | 11.9907 | 3.592 |  | Rice | Brown Indian Hemp | Garden pea | Athos | Cowpea | IT90K-76 | Cotton | Varamin |
| Madagascar | Bingaguru, eastern Zimbabwe | -18.7589 | 32.6343 |  | Sorghum | Brussel sprout | Garden pea | Messire | Cowpea | IT93K452-1 | Cotton | Stam 4224 |
| Mali | Bondo, Kenya | -0.0949 | 34.2762 |  | Millet | Butternut squash | Lentil | Kırmızı-Local | Cowpea | IT93K-8-45-5-1-5 | Cowpea | IT-90K-284-2 |
| Nepal | Borno state, northeast Nigeria | 11.8333 | 10.4166 |  | Sunflower | Cabbage | Lentil | L-317 | Cowpea | Kavara | Cowpea | IT-90K-284-2 |
| Niger | Bugiri, Uganda | 0.5683 | 33.7494 |  | Tobacco | Canola | Maize | 0804-7STR | Cowpea | Suvita | Cowpea | IT93K452-1 |
| Nigeria | Bungoma, Kenya | 0.5693 | 34.5559 |  | Tomato | Cauliflower | Maize | 2000SYN-EE-W-STR | Cowpea | TVX – 1850-01F | Endive | crispum |
| Spain | Bunyore, Vihiga District, Kenya | 0.1111 | 34.5666 |  | Wild lentil | Celery | Maize | 2004TZE-W-DT-STR-C4 | Cowpea | Vya | Flax | Legina |
| The Gambia | Busia district, Kenya | 0.4599 | 34.1091 |  |  | Celosia argentia | Maize | 8322-13 | Fenugreek | Giza 2 | Foxtail millet | Cao Guzi |
| Tunisia | Busia, Uganda | 0.4661 | 34.0889 |  |  | Cereal | Maize | 8338-1 | Groundnut | Ex-Dakar | Foxtail millet | Jingu 29 |
| Turkey | Butere, Kenya | 0.2162 | 34.4921 |  |  | Chickpea | Maize | 8428-19 | Groundnut | Homabay | Giant spinach | Epinard greant |
| U.S.A | Cameroon | 10.5925 | 14.32101 |  |  | Chilli | Maize | 94TZE COMP5-W | Groundnut | ICGV 907048SM | Groundnut | RMP12 |
| Uganda | Chinyudze, eastern Zimbabwe | -18.1866 | 32.2005 |  |  | Clusterbean | Maize | 99EVDTSTR-W | Groundnut | Red Beauty | Lentil | Kırmızı-Local |
| Zimbabwe | Clackamas County, Oregon, USA | 45.1903 | -122.2023 |  |  | Common bean | Maize | 99TZEE-Y-STR | Groundnut | RMP-12 | Maize | 94TZE COMP5-W |
|  | Cordoba, Spain | 37.8921 | -4.7831 |  |  | Common vetch | Maize | ACROSS 97 TZL COMP1-W | Groundnut | RMP-91 | Maize | ACROSS 97 TZL COMP1-W |
|  | Ebuyangu, Vihiga District, Kenya | 0.1 | 34.5833 |  |  | Coriander | Maize | DMR-ESRW | Groundnut | S28/206 | Maize | H19 |
|  | Emabwi, Vihiga District, Kenya | 0.1 | 34.5833 |  |  | Cotton | Maize | Hybrid 511 | Groundnut | Yarkasa | Maize | N314 |
|  | Gotulis, Bawku district, Ghana | 11.0166 | -0.2666 |  |  | Cowpea | Maize | Hybrid 614 | Lupin | Giza 2 | Maize | Oba Super 1 |
|  | Govakova, eastern Zimbabwe | -18.7594 | 32.6323 |  |  | Mucuna | Maize | IRAT 200 | Lupin | Ultra | Maize | Q67 |
|  | Guyuan, Ningxia Hui Region, China | 35.9988 | 106.4191 |  |  | Crotalaria grahamiana | Maize | Longe 5 | Mung bean | Local | Maize | TZE COMP3 DT |
|  | Haifa, Israel | 32.7872 | 35.0031 |  |  | Crotalaria juncea | Maize | Oba Super 1 | Oat | Cory | Maize | TZL COMP1 SYN |
|  | Homa Bay, Kenya | -0.5375 | 34.4563 |  |  | Crotalaria ochroleuca | Maize | SC501 | Pepper | Shalhevet | Maize | Z6 |
|  | Ibadan, Nigeria | 7.4909 | 3.8945 |  |  | Cucumber | Maize | TZE COMP3 DT | Soya bean | EAI 3600 | Millet | Chalak |
|  | Isfahan, Iran | 32.7193 | 51.5321 |  |  | Cucumis prophetarum | Maize | TZL COMP1 SYN | Soya bean | Jupiter | Mung bean | Parto |
|  | Ivory, Mid-west Madagascar | 46.4112 | -19.5524 |  |  | Cumin | Maize | TZSR-W-1 | Soya bean | SAMSOY II | Pepper | Arkalohit |
|  | Kaduna, northern Nigeria | 10.7251 | 7.8683 |  |  | Desmodium distortum | Maize | Western Yellow | Soya bean | Tgm1039 | Pepper | Jinghong |
|  | Kafr-El Sheikh, Egypt | 31.1048 | 30.9435 |  |  | Desmodium intortum | Maize | WH403 | Soya bean | Tgm1576 | Pepper | Qingdao Xinlilai |
|  | Kano / Katsina, Nigeria | 11.9918 | 8.5209 |  |  | Desmodium uncinatum | Maize | WH502 | Soya bean | TGx 1448-2E / TGx 1864 | Pepper | Zi jinshan |
|  | Karaj, Iran | 35.8228 | 50.9583 |  |  | Desmodium intortum | Maize | WH505 | Soya bean | TGX1448-2E | Pigeon pea | ICPL 87091 |
|  | Kaya, Nigeria | 11.254 | 7.2389 |  |  | Desmodium spp | Maize | WH507 | Soya bean | TGX1876-4E | Sesame | Darab1 |
|  | Kibos, Kisumu dirtsict, Kenya | 0.0333 | 34.8001 |  |  | Desmodium uncinatum | Maize | WH511 | Soya bean | TXG1448-2E | Soya bean | Duika |
|  | Kisii, Kenya | -0.6792 | 34.7748 |  |  | Dill | Maize | WH513 | Triticale | Penarroya | Soya bean | TGx 1864 |
|  | Kisumu dirtsict, Kenya | -0.0661 | 34.7766 |  |  | Egyptian clover | Maize | WH624 | Wheat | Alamut | Soya bean | TGX1448-2E |
|  | Kumi District, Uganda | 1.4676 | 33.9341 |  |  | Endive | Millet | Manga Nara | Wheat | Alvand | Soya bean | TGx1740-2F |
|  | Kuria, Kenya | -1.2212 | 34.5449 |  |  | Faba bean | Mung bean | Pusa 105 | Wheat | Baiat | Soya bean | TGx1740-7F |
|  | Lambwe, Suba district, western Kenya | -0.5492 | 34.3638 |  |  | Faidherbia albida | Pea | Syrian local | Wheat | Chamran | Sugar beet | 143 |
|  | Layin Taki and Kayawa, northern Nigeria | 12.9568 | 8.1441 |  |  | Fallow | Red clover | Kenland | Wheat | Falat | Sugar beet | RG8001 |
|  | Lower River Division, The Gambia | 12.5524 | -15.9361 |  |  | Fenugreek | Sorghum | BES (KSV4) | Wheat | Kavir | Sugar beet | Ruima |
|  | Mahuta, Nigeria | 10.5002 | 7.5275 |  |  | Flax | Sorghum | Damougari/S35 | Wheat | Sepahan | Sunflower | Hybrid 8998 |
|  | Maiduguri, Nigeria | 11.8045 | 13.1966 |  |  | Foxtail millet | Sorghum | Djigari | Wheat | TRI11554 | Triticale | Bogo |
|  | Makerere University, Uganda | 0.3277 | 32.5674 |  |  | Garden pea | Sorghum | Gadam Hamam | Wheat | TRI11712 | Turnip | Local-Bursa Tohum |
|  | Mansajang Kunda, Gambia | 13.2867 | -14.1931 |  |  | Garlic | Sorghum | Ganseber | Wheat | TRI15593 | Wheat | Xinchun 6 M |
|  | McCarthy Island north, The Gambia | 12.8667 | -15.2163 |  |  | Giant spinach | Sorghum | ICSV 1002 | Wheat | TRI17606 | Wheat | Yongliang 15 |
|  | Melkassa, Ethiopia | 8.4056 | 39.3285 |  |  | Gourd | Sorghum | ICSV 1007 | Wheat | TRI18664 | Winter durum wheat | Connie |
|  | Merti, Ethiopia | 8.8714 | 39.9148 |  |  | Groundnut | Sorghum | Kadaga | Wheat | TRI19322 | Winter wheat | Foote |
|  | Migori, Kenya | -1.0675 | 34.4665 |  |  | Lentil | Sorghum | KSV8 | Wheat | TRI19652 | Winter wheat | Gene |
|  | Nara, Mali | 15.1657 | -7.2872 |  |  | Linseed | Sorghum | Kutbie | Wheat | TRI7259 | Winter wheat | Madsen |
|  | Nawalparasi, Nepal | 27.6475 | 83.9354 |  |  | Lupin | Sorghum | Sama Jabo |  |  | Winter wheat | Stephens |
|  | NGS, Borno state, northeast Nigeria | 10.6578 | 12.2668 |  |  | Maize | Sorghum | Ware warenbashi |  |  | Winter wheat | Weatherford |
|  | Nipani, Karnataka, India | 16.4084 | 74.3746 |  |  | Melon | Sorghum | wediaker |  |  | Winter wheat | Yamhill |
|  | North Bank Division, The Gambia | 12.6441 | -16.7006 |  |  | Mung bean | Sorghum | Mobal |  |  |  |  |
|  | Nyabeda, western Kenya | 0.1276 | 34.4007 |  |  | Mustard | Sunflower | Aidatou |  |  |  |  |
|  | Nyando, Kenya | -0.2011 | 35.0133 |  |  | Narbon vetch | Sunflower | T33 |  |  |  |  |
|  | Rachuonyo, Kenya | -0.5062 | 34.7322 |  |  | Oat | Tobacco | Anand-119 |  |  |  |  |
|  | Rimau, Nigeria | 10.4378 | 7.7533 |  |  | Okra | Tomato | M-82 |  |  |  |  |
|  | Rongo, Kenya | -0.7559 | 34.5981 |  |  | Onion | Tomato | Pomodoro ACE 55vF |  |  |  |  |
|  | Rongo, Kenya | -0.7559 | 34.5981 |  |  | Parsley | Tomato | Roma vf |  |  |  |  |
|  | Sadore, Niger | 13.2317 | 2.2756 |  |  | Pepper | Tomato | Shifan 33 |  |  |  |  |
|  | Sapu, Gambia | 13.5486 | -14.8987 |  |  | Pigeon pea | Tomato | Super Luna |  |  |  |  |
|  | SGS, Borno state, northeast Nigeria | 10.4346 | 11.8435 |  |  | Proso millet | Wild lentil | LENS166/92 |  |  |  |  |
|  | Sheraro, Tigray, Ethiopia | 14.3947 | 37.7723 |  |  | Radish |  |  |  |  |  |  |
|  | Siaya, Kenya | 0.0476 | 34.2869 |  |  | Rapeseed |  |  |  |  |  |  |
|  | Some` , Za-Kpota, Benin | 7.2167 | 2.1997 |  |  | Red cabbage |  |  |  |  |  |  |
|  | SS, Borno state, northeast Nigeria | 11.1527 | 12.7897 |  |  | Ricebean |  |  |  |  |  |  |
|  | Suba district, western Kenya | -0.4303 | 34.2069 |  |  | Roselle |  |  |  |  |  |  |
|  | Tahtay Maychew district, Tigray, Ethiopia | 12.7929 | 39.5277 |  |  | Senna didymobotrya |  |  |  |  |  |  |
|  | Tarime, Tanzania | -1.3429 | 34.3771 |  |  | Senna occidentalis |  |  |  |  |  |  |
|  | Terudig, Bawku district, Ghana | 11.0166 | -0.2666 |  |  | Senna spectabilis |  |  |  |  |  |  |
|  | Teso, Kenya | 0.4608 | 34.1129 |  |  | Sesame |  |  |  |  |  |  |
|  | Tororo, Uganda | 0.6829 | 34.1779 |  |  | Sesbania cinerascens |  |  |  |  |  |  |
|  | Trans Nzoia, Kenya | 1.0533 | 34.9874 |  |  | Sesbania sesban |  |  |  |  |  |  |
|  | Uganda | 0.9672 | 33.9183 |  |  | Silverleaf nightshade |  |  |  |  |  |  |
|  | University of Stuttgart, Germany | 48.7811 | 9.1736 |  |  | Smooth vetch |  |  |  |  |  |  |
|  | Upper River north, The Gambia | 12.8412 | -15.1736 |  |  | Snap bean |  |  |  |  |  |  |
|  | Usha, Israel | 32.7957 | 35.1134 |  |  | Sorhgum |  |  |  |  |  |  |
|  | Vihiga, Kenya | 0.0502 | 34.6915 |  |  | Soya bean |  |  |  |  |  |  |
|  | Vijayawada, Andhra Pradesh, India | 16.5369 | 80.6744 |  |  | Spinach |  |  |  |  |  |  |
|  | Western Division, The Gambia | 12.4626 | -16.4968 |  |  | Squash |  |  |  |  |  |  |
|  | Xianyang, Shaanxi, China | 34.2619 | 108.0729 |  |  | Squirting cucumber |  |  |  |  |  |  |
|  | Ziway, Ethiopia | 7.9304 | 38.7151 |  |  | Stylosanthes guianensis | |  |  |  |  |  |
|  |  |  |  |  |  | Sugar beet |  |  |  |  |  |  |
|  |  |  |  |  |  | Sunflower |  |  |  |  |  |  |
|  |  |  |  |  |  | Sweet potao |  |  |  |  |  |  |
|  |  |  |  |  |  | Syrian oregano |  |  |  |  |  |  |
|  |  |  |  |  |  | Tephrosia vogelii |  |  |  |  |  |  |
|  |  |  |  |  |  | Tithonia diversifolia |  |  |  |  |  |  |
|  |  |  |  |  |  | Tomato |  |  |  |  |  |  |
|  |  |  |  |  |  | Triticale |  |  |  |  |  |  |
|  |  |  |  |  |  | Turnip |  |  |  |  |  |  |
|  |  |  |  |  |  | Vigna mungo |  |  |  |  |  |  |
|  |  |  |  |  |  | Watermelon |  |  |  |  |  |  |
|  |  |  |  |  |  | Wheat |  |  |  |  |  |  |
|  |  |  |  |  |  | Wild rue |  |  |  |  |  |  |
|  |  |  |  |  |  | Winter durum wheat |  |  |  |  |  |  |
|  |  |  |  |  |  | Winter wheat |  |  |  |  |  |  |
