# Supplementary figures and images for: Crop diversification and parasitic weed abundance: a global meta-analysis
Source: Sci Rep. 2022 Nov 12;12:19413. doi: 10.1038/s41598-022-24047-2 (PMC9653488; doi:10.1038/s41598-022-24047-2)

A

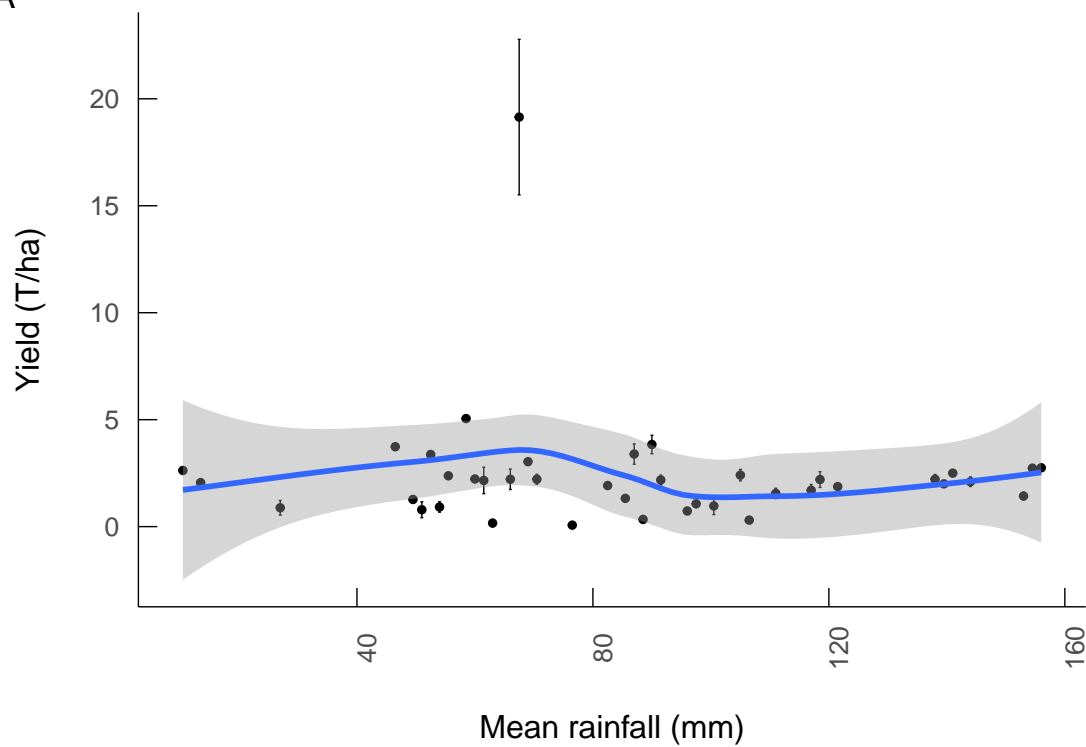

C

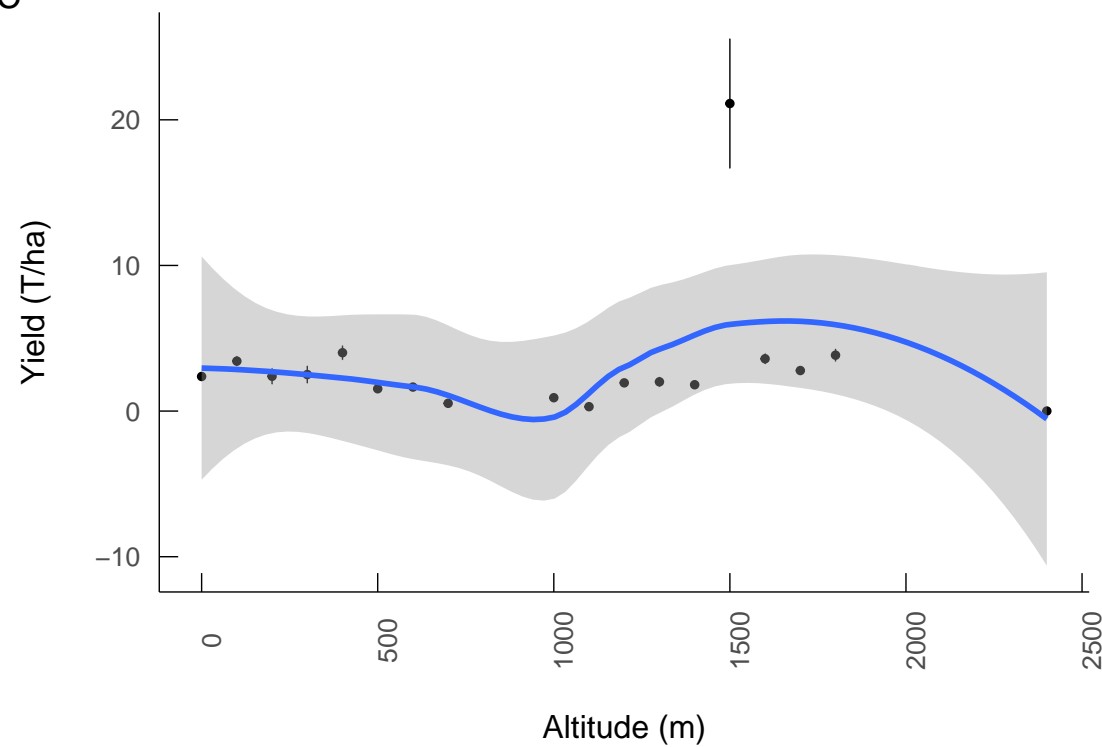

B

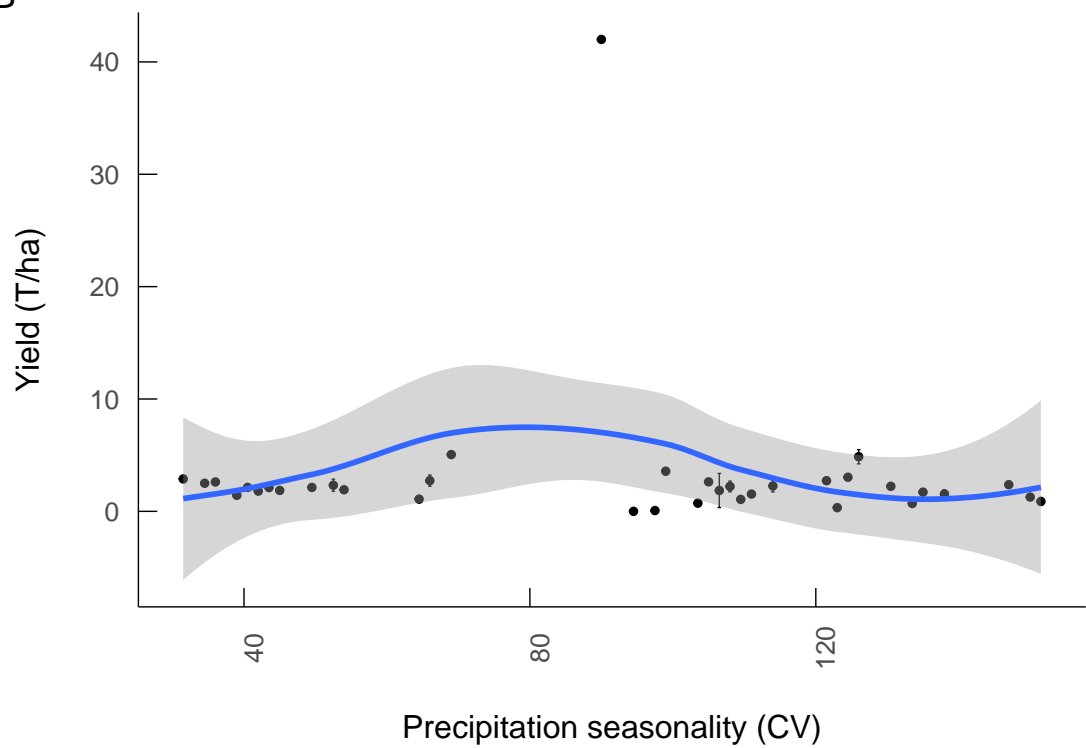

D

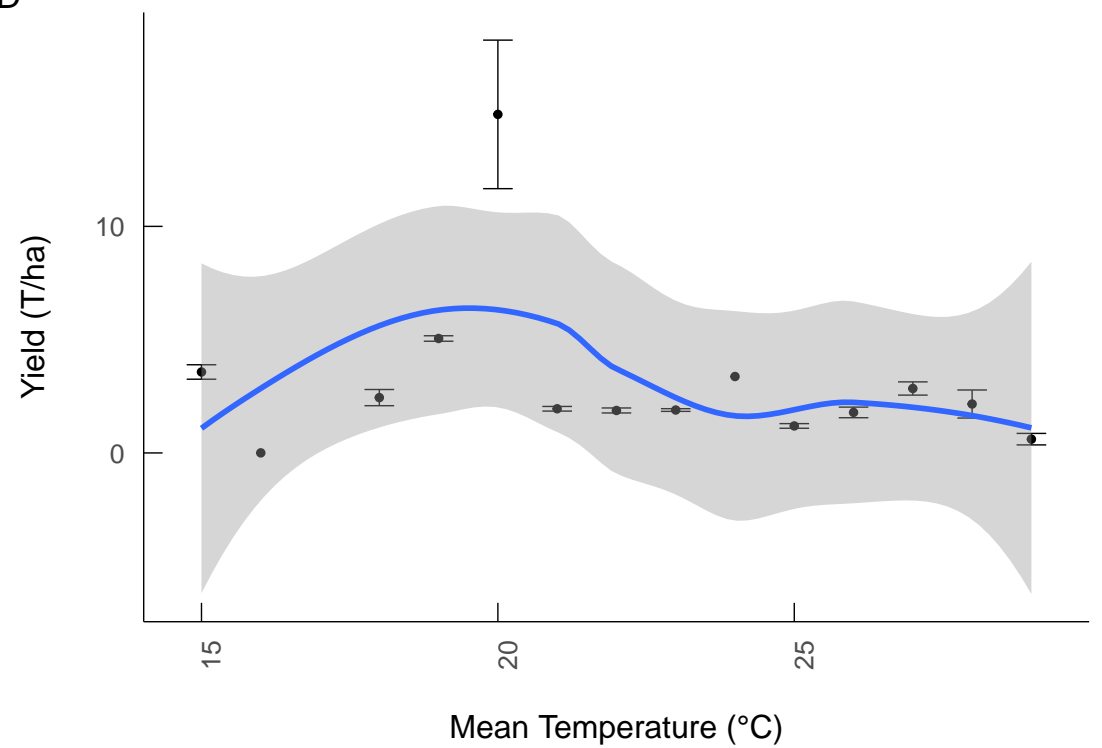

Supplement: Supplementary file 7 — Supplementary Information 7. [file 41598_2022_24047_MOESM7_ESM.pdf]

# A

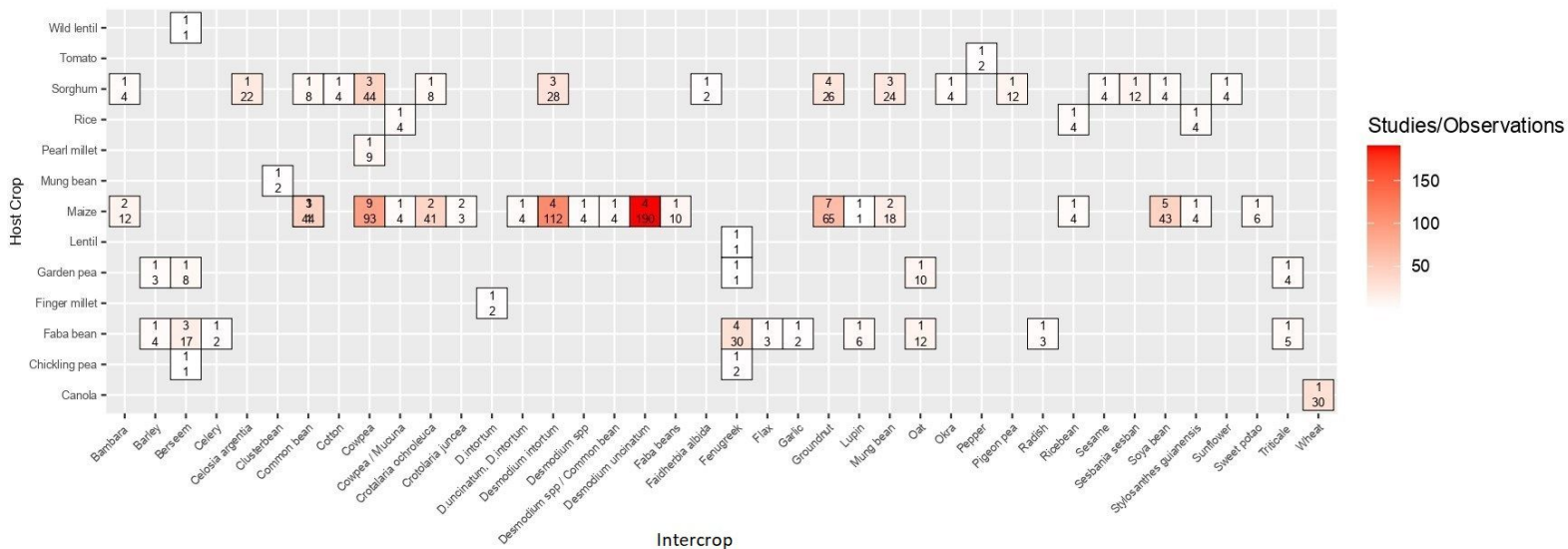

B

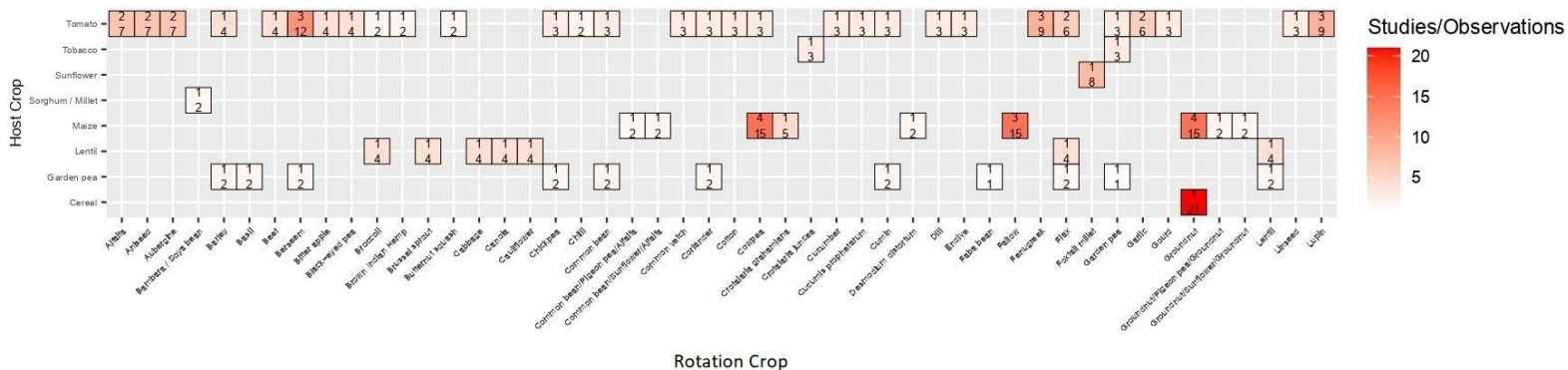

C

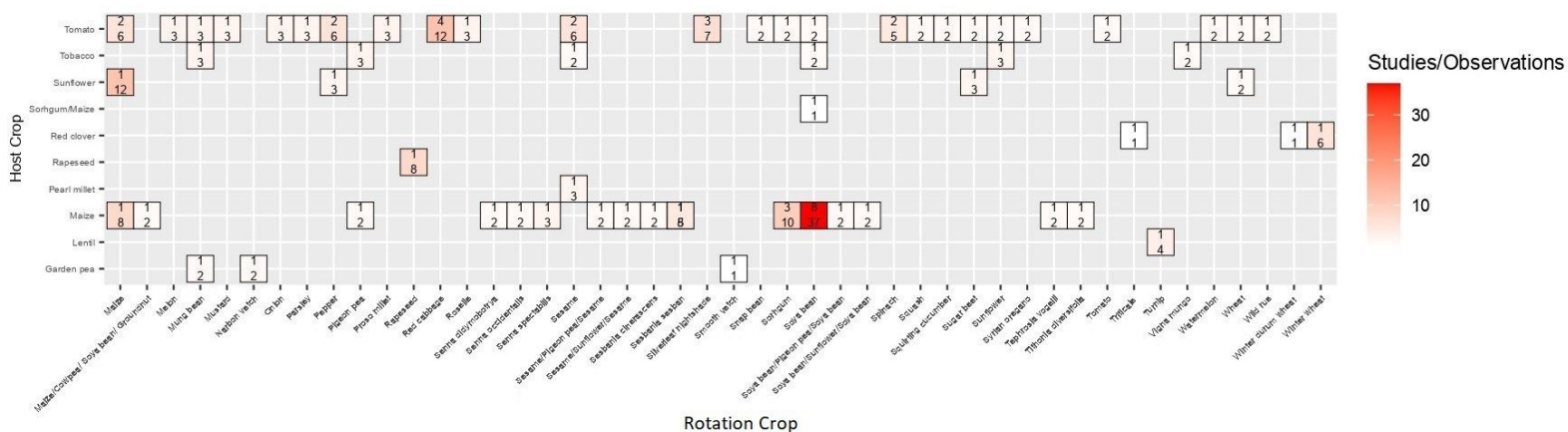

Supplement: Supplementary file 8 — Supplementary Information 8. [file 41598_2022_24047_MOESM8_ESM.pdf]

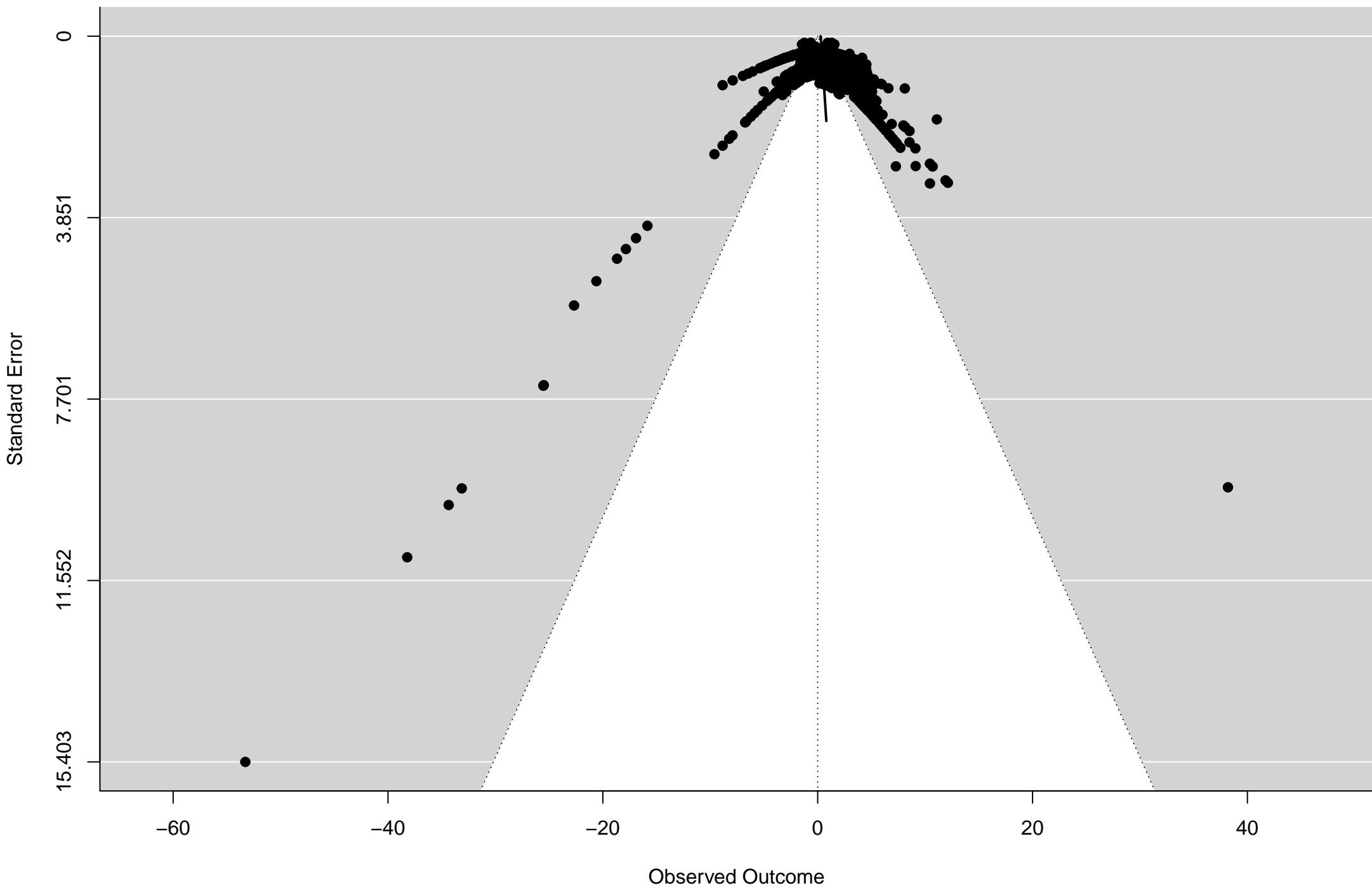

Supplement: Supplementary file 9 — Supplementary Information 9. [file 41598_2022_24047_MOESM9_ESM.pdf]

A

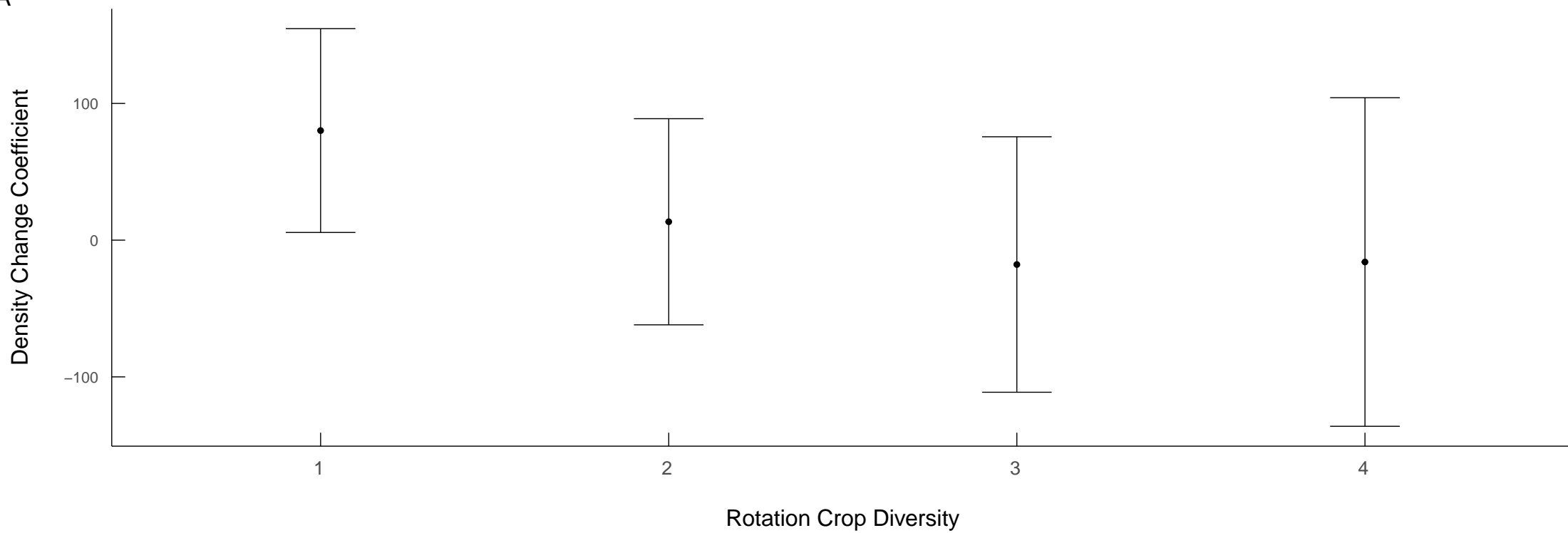

B

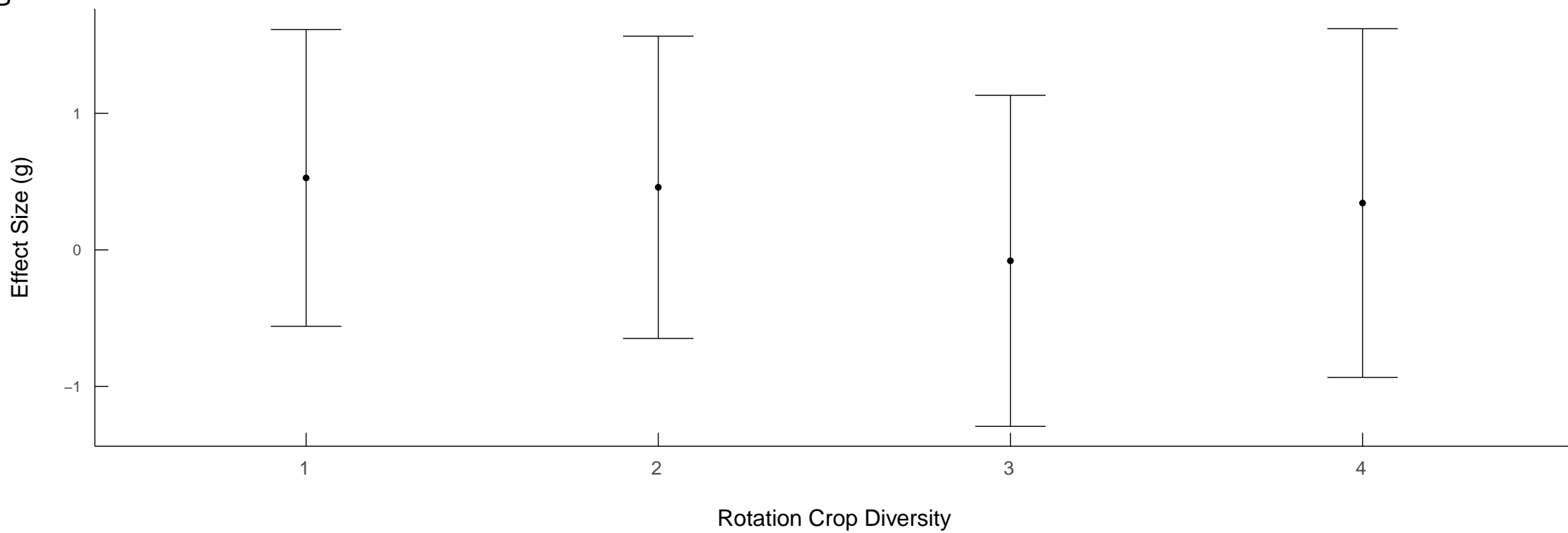

Supplement: Supplementary file 11 — Supplementary Information 11. [file 41598_2022_24047_MOESM11_ESM.pdf]
