# Supplementary material for: Crop diversification and parasitic weed abundance: a global meta-analysis
Source: Sci Rep. 2022 Nov 12;12:19413. doi: 10.1038/s41598-022-24047-2 (PMC9653488; doi:10.1038/s41598-022-24047-2)

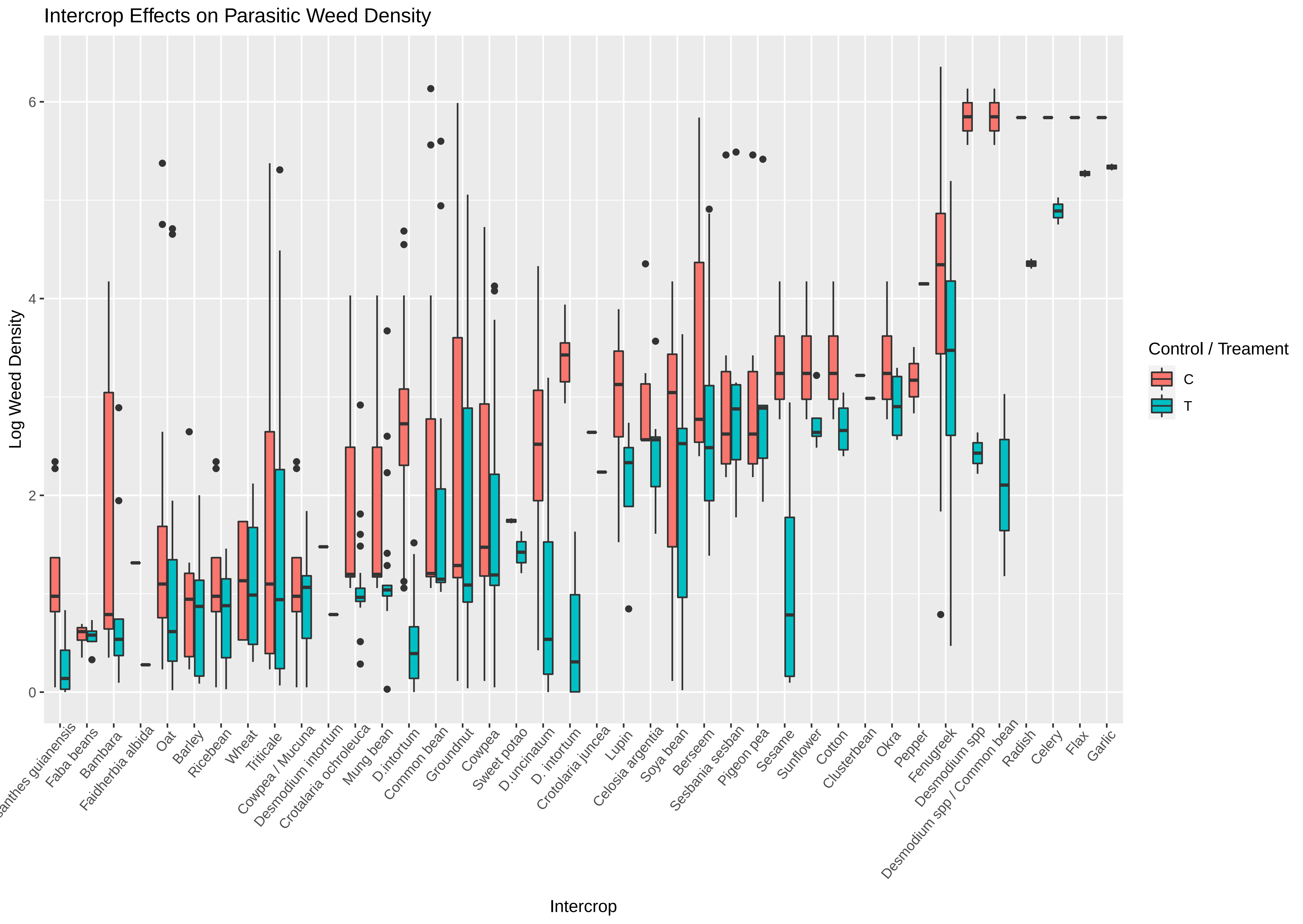

Intercrop Effects on Parasitic Weed Density
Log Weed Density
0
2
4
6
Intercrop
Control / Treament
C
T
santhes guianensis
Faba beans
Bambara
Faidherbia albida
Oat
Barley
Ricebean
Wheat
Triticale
Cowpea / Mucuna
Desmodium intortum
Crotalaria ochroleuca
Mung bean
D.intortum
Common bean
Groundnut
Cowpea
Sweet potao
D.uncinatum
D. intortum
Crotolaria juncea
Lupin
Celosia argentia
Soya bean
Berseem
Sesbania sesban
Pigeon pea
Sesame
Sunflower
Cotton
Clusterbean
Okra
Pepper
Fenugreek
Desmodium spp
Desmodium spp / Common bean
Radish
Celery
Flax
Garlic

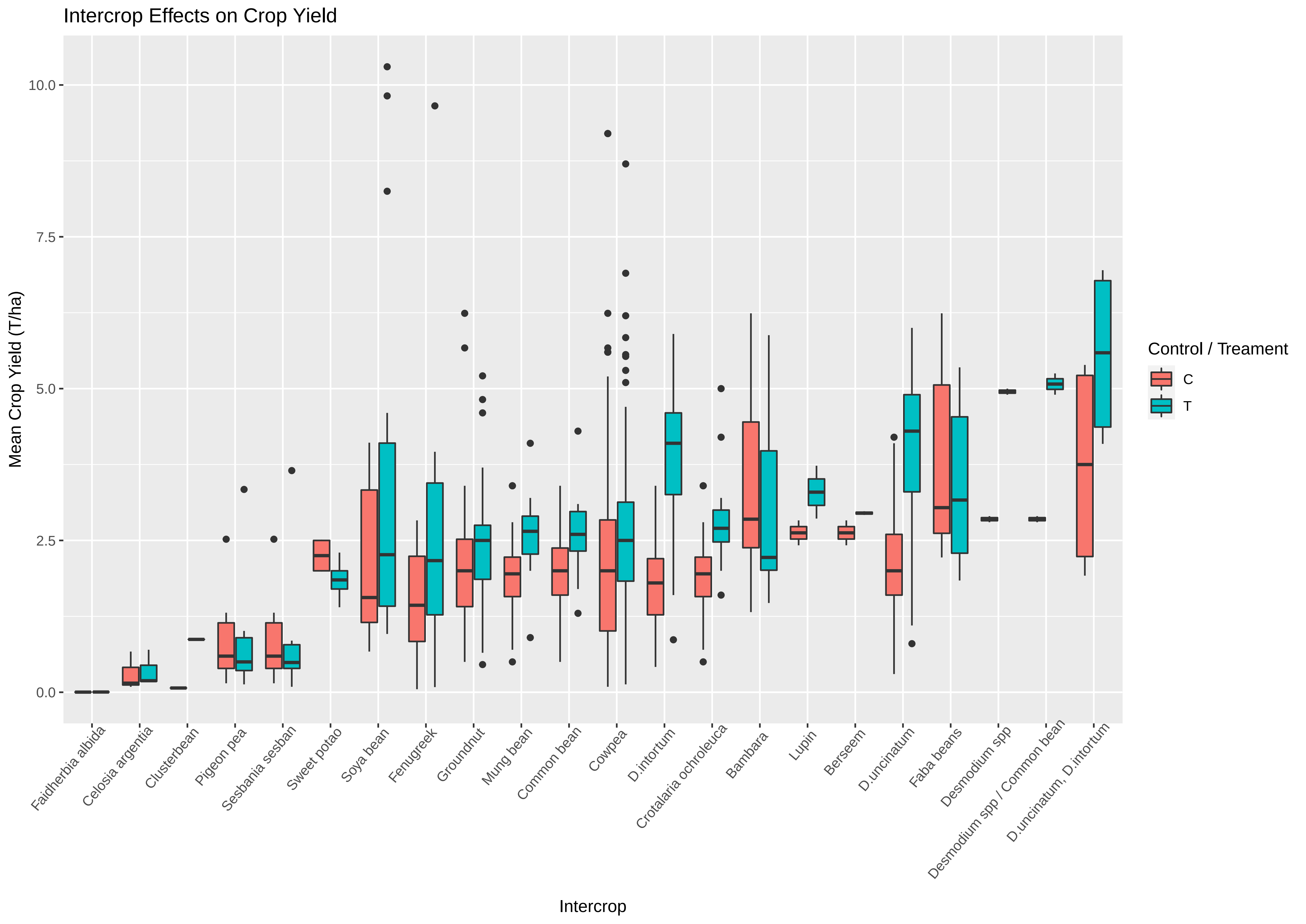

Intercrop Effects on Crop Yield
Mean Crop Yield (T/ha)
10.0
7.5
5.0
2.5
0.0
Faidherbia albida
Celosia argentia
Clusterbean
Pigeon pea
Sesbania sesban
Sweet potao
Soya bean
Fenugreek
Groundnut
Mung bean
Common bean
Cowpea
D.intortum
Crotalaria ochroleuca
Bambara
Lupin
Berseem
D.uncinatum
Faba beans
Desmodium spp
Desmodium spp / Common bean
D.uncinatum, D.intortum
Intercrop
Control / Treament
C
T

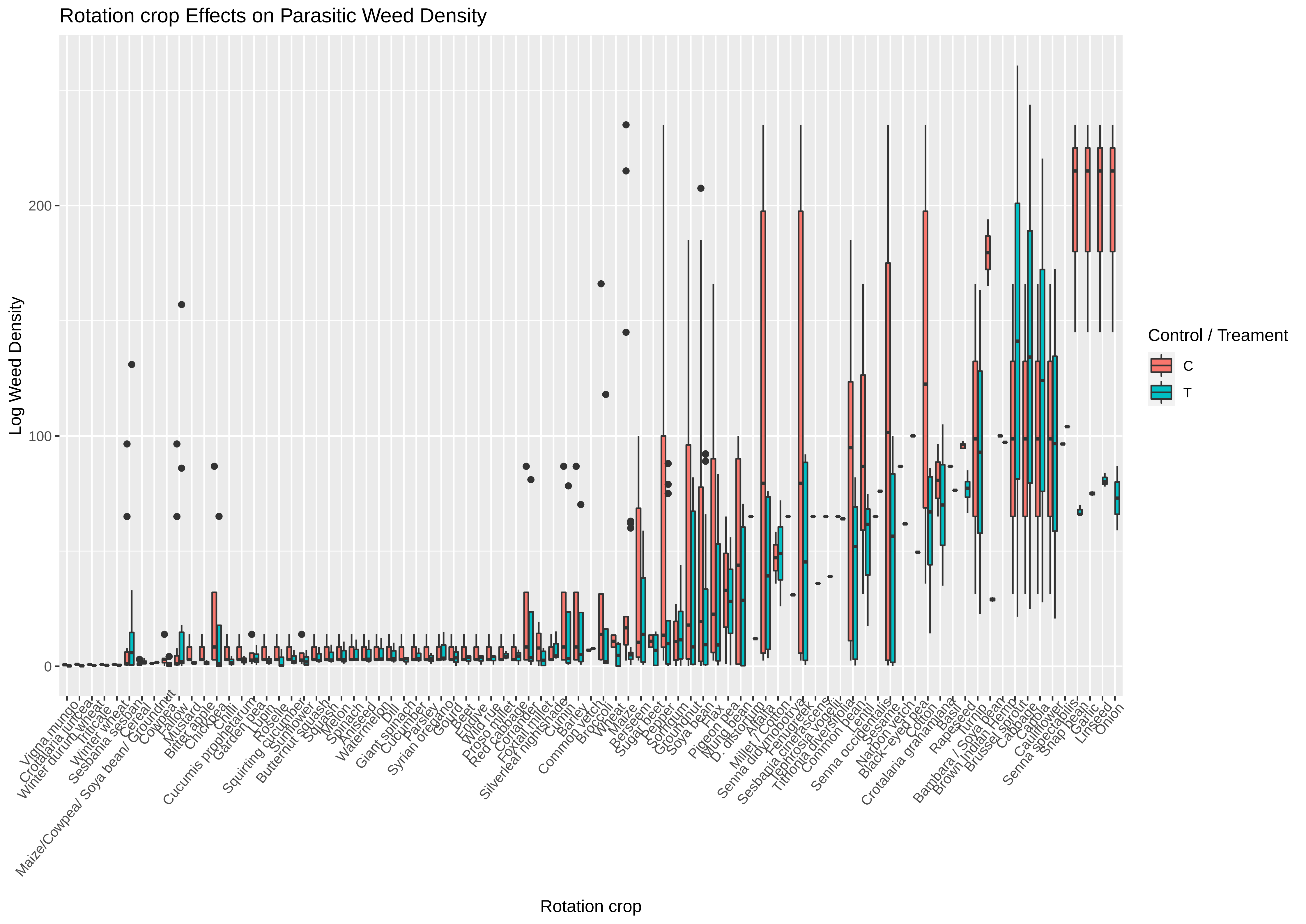
Rotation crop Effects on Parasitic Weed Density
Log Weed Density
200
100
0
Rotation crop
Vigna mungo
Crotalaria juncea
Winter durum wheat
Triticale
Winter wheat
Sesbania sesban
Cereal
Maize/Cowpea/ Soya bean/ Groundnut
Cowpea
Fallow
Mustard
Bitter apple
Chickpea
Chilli
Cucumis prophetarum
Garden pea
Lupin
Roselle
Squirting cucumber
Sunflower
Butternut squash
Squash
Melon
Spinach
Aniseed
Watermelon
Dill
Giant spinach
Cucumber
Parsley
Syrian oregano
Gourd
Beet
Endive
Wild rue
Proso millet
Red cabbage
Coriander
Foxtail millet
Silverleaf nightshade
Cumin
Barley
Common vetch
Broccoli
Wheat
Maize
Berseem
Sugar beet
Pepper
Sorghum
Groundnut
Soya bean
Flax
Pigeon pea
Mung bean
D. distortum
Alfalfa
Millet / Cotton
Senna didymobotrya
Fenugreek
Sesbania cinerascens
Tephrosia vogelii
Tithonia diversifolia
Common bean
Lentil
Senna occidentalis
Sesame
Narbon vetch
Black−eyed pea
Cotton
Crotalaria grahamiana
Basil
Rapeseed
Turnip
Bambara / Soya bean
Brown indian Hemp
Brussel sprout
Cabbage
Canola
Cauliflower
Senna spectabilis
Snap bean
Garlic
Linseed
Onion
Control / Treament
C
T

Rotation Crop Effects on Crop Yield

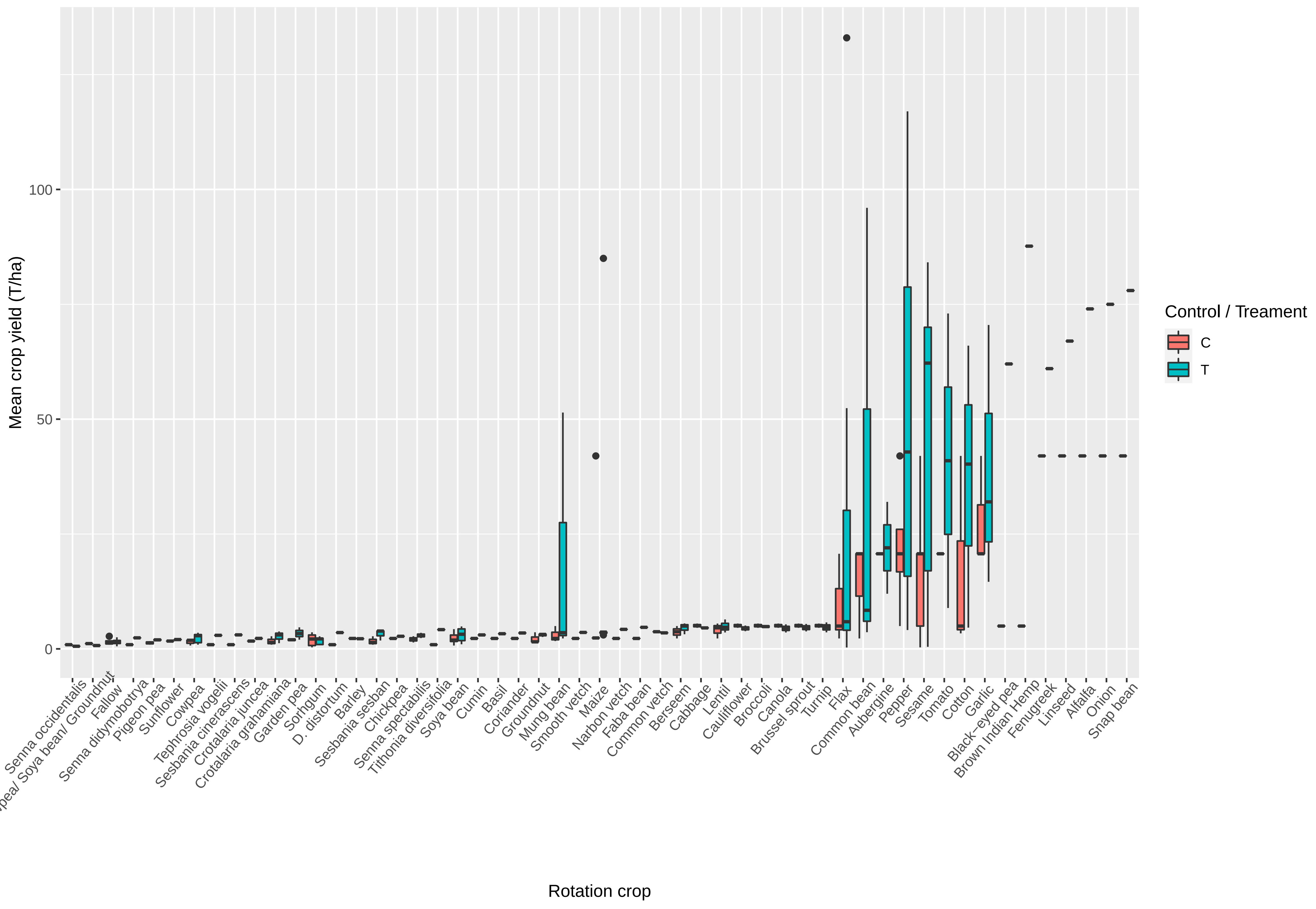

Supplement: Supplementary file 10 — Supplementary Information 10. [file 41598_2022_24047_MOESM10_ESM.pdf]
