# Supplementary material for: Crop diversification and parasitic weed abundance: a global meta-analysis
Source: Sci Rep. 2022 Nov 12;12:19413. doi: 10.1038/s41598-022-24047-2 (PMC9653488; doi:10.1038/s41598-022-24047-2)
Supplement: Supplementary file 12 — Supplementary Information 12. [file 41598_2022_24047_MOESM12_ESM.docx]

> Hedges_d_and_var_for_checks<-read.csv("Hedges d and var for checks.CSV")

> View(Hedges_d_and_var_for_checks)

> fsn(Effect.Size, Variance, data=Hedges_d_and_var_for_checks, type="Rosenberg")

Fail-safe N Calculation Using the Rosenberg Approach

Average Effect Size: 0.3965

Observed Significance Level: <.0001

Target Significance Level: 0.05

Fail-safe N: 311129

> regtest(Effect.Size, Variance, model="rma", predictor="Variance", ret.fit=FALSE, digits =4)

Error in regtest(Effect.Size, Variance, model = "rma", predictor = "Variance", :

object 'Effect.Size' not found

> library(metafor)

> setwd("C:/Users/Ragenaky/Desktop/Thesis chapter 3/Data/Bias Calc/FailSafeCalc/Hedges D and var for checks")

>

> Hedges_d_and_var_for_checks<-read.csv("Hedges d and var for checks.CSV")

>

> fsn(Effect.Size, Variance, data=Hedges_d_and_var_for_checks, type="Rosenberg")

Fail-safe N Calculation Using the Rosenberg Approach

Average Effect Size: 0.3965

Observed Significance Level: <.0001

Target Significance Level: 0.05

Fail-safe N: 311129

> regtest(Effect.Size, Variance, model="rma", predictor="Variance", ret.fit=FALSE, digits =4)

Error in regtest(Effect.Size, Variance, model = "rma", predictor = "Variance", :

object 'Effect.Size' not found

> res <- rma(Effect.Size, Variance, data=Hedges_d_and_var_for_checks)

Warning message:

Studies with NAs omitted from model fitting.

> res

Random-Effects Model (k = 1517; tau^2 estimator: REML)

tau^2 (estimated amount of total heterogeneity): 2.5348 (SE = 0.1098)

tau (square root of estimated tau^2 value): 1.5921

I^2 (total heterogeneity / total variability): 89.37%

H^2 (total variability / sampling variability): 9.41

Test for Heterogeneity:

Q(df = 1516) = 11578.0658, p-val < .0001

Model Results:

estimate se zval pval ci.lb ci.ub

0.4673 0.0449 10.3990 <.0001 0.3793 0.5554 ***

---

Signif. codes: 0 ‘***’ 0.001 ‘**’ 0.01 ‘*’ 0.05 ‘.’ 0.1 ‘ ’ 1

> res

Random-Effects Model (k = 1517; tau^2 estimator: REML)

tau^2 (estimated amount of total heterogeneity): 2.5348 (SE = 0.1098)

tau (square root of estimated tau^2 value): 1.5921

I^2 (total heterogeneity / total variability): 89.37%

H^2 (total variability / sampling variability): 9.41

Test for Heterogeneity:

Q(df = 1516) = 11578.0658, p-val < .0001

Model Results:

estimate se zval pval ci.lb ci.ub

0.4673 0.0449 10.3990 <.0001 0.3793 0.5554 ***

---

Signif. codes: 0 ‘***’ 0.001 ‘**’ 0.01 ‘*’ 0.05 ‘.’ 0.1 ‘ ’ 1

> regtest(res, model="lm")

Regression Test for Funnel Plot Asymmetry

Model: weighted regression with multiplicative dispersion

Predictor: standard error

Test for Funnel Plot Asymmetry: t = 2.2836, df = 1515, p = 0.0225

Limit Estimate (as sei -> 0): b = 0.1947 (CI: 0.0052, 0.3841)

> reg <- regtest(res)

> reg

Regression Test for Funnel Plot Asymmetry

Model: mixed-effects meta-regression model

Predictor: standard error

Test for Funnel Plot Asymmetry: z = 2.0058, p = 0.0449

Limit Estimate (as sei -> 0): b = 0.2716 (CI: 0.0614, 0.4819)

> reg
