# Supplementary material for: Crop diversification and parasitic weed abundance: a global meta-analysis
Source: Sci Rep. 2022 Nov 12;12:19413. doi: 10.1038/s41598-022-24047-2 (PMC9653488; doi:10.1038/s41598-022-24047-2)
Supplement: Supplementary file 13 — Supplementary Information 13. [file 41598_2022_24047_MOESM13_ESM.docx]

>

>

>

> rm(list=ls())# wipes slate clean

> library(mgcv)

> library(lme4)

> library(lmerTest)

> library(ggplot2)

> library(dplyr)

> library( geosphere )

> library( stringr)

> #Calculate a standard error

> stderr <- function(x, ...) sd(x, na.rm = TRUE) / sqrt(length(is.na(x == FALSE)) )

> ### Install this When you start for Multiplots!!!#####

> #

> # ggplot objects can be passed in ..., or to plotlist (as a list of ggplot objects)

> # - cols: Number of columns in layout

> # - layout: A matrix specifying the layout. If present, 'cols' is ignored.

> #

> # If the layout is something like matrix(c(1,2,3,3), nrow=2, byrow=TRUE),

> # then plot 1 will go in the upper left, 2 will go in the upper right, and

> # 3 will go all the way across the bottom.

> #

> multiplot <- function(..., plotlist=NULL, file, cols=1, layout=NULL) {

+ library(grid)

+

+ # Make a list from the ... arguments and plotlist

+ plots <- c(list(...), plotlist)

+

+ numPlots = length(plots)

+

+ # If layout is NULL, then use 'cols' to determine layout

+ if (is.null(layout)) {

+ # Make the panel

+ # ncol: Number of columns of plots

+ # nrow: Number of rows needed, calculated from # of cols

+ layout <- matrix(seq(1, cols * ceiling(numPlots/cols)),

+ ncol = cols, nrow = ceiling(numPlots/cols))

+ }

+

+ if (numPlots==1) {

+ print(plots[[1]])

+

+ } else {

+ # Set up the page

+ grid.newpage()

+ pushViewport(viewport(layout = grid.layout(nrow(layout), ncol(layout))))

+

+ # Make each plot, in the correct location

+ for (i in 1:numPlots) {

+ # Get the i,j matrix positions of the regions that contain this subplot

+ matchidx <- as.data.frame(which(layout == i, arr.ind = TRUE))

+

+ print(plots[[i]], vp = viewport(layout.pos.row = matchidx$row,

+ layout.pos.col = matchidx$col))

+ }

+ }

+ }

>

>

> #Fig 4a

>

> Open_Data_IC_RC_WD<-read.csv("Open_Data_IC_RC_WD.CSV")

>

> # Mean rainfall

> model1 <- lm( log( Control_Mean + 1) ~ Mean_RF, data = Open_Data_IC_RC_WD )

> anova(model1)

Analysis of Variance Table

Response: log(Control_Mean + 1)

Df Sum Sq Mean Sq F value Pr(>F)

Mean_RF 1 95.74 95.737 32.578 1.691e-08 ***

Residuals 701 2060.05 2.939

---

Signif. codes:

0 ‘***’ 0.001 ‘**’ 0.01 ‘*’ 0.05 ‘.’ 0.1 ‘ ’ 1

> summary(model1)

Call:

lm(formula = log(Control_Mean + 1) ~ Mean_RF, data = Open_Data_IC_RC_WD)

Residuals:

Min 1Q Median 3Q Max

-3.3773 -1.3805 -0.0301 1.6653 4.4122

Coefficients:

Estimate Std. Error t value Pr(>|t|)

(Intercept) 3.982144 0.167945 23.711 < 2e-16 ***

Mean_RF -0.009896 0.001734 -5.708 1.69e-08 ***

---

Signif. codes:

0 ‘***’ 0.001 ‘**’ 0.01 ‘*’ 0.05 ‘.’ 0.1 ‘ ’ 1

Residual standard error: 1.714 on 701 degrees of freedom

Multiple R-squared: 0.04441, Adjusted R-squared: 0.04305

F-statistic: 32.58 on 1 and 701 DF, p-value: 1.691e-08

>

> Open_Data_IC_WDA <- Open_Data_IC_RC_WD

> Open_Data_IC_WDA$rainCat <- round(Open_Data_IC_WDA$ Mean_RF / 1.5) * 1.5

> summaryRain <- Open_Data_IC_WDA %>%

+ group_by( rainCat ) %>%

+ summarise( meanN = mean(log( Control_Mean + 1), na.rm = TRUE), SE = stderr(log( Control_Mean + 1), na.rm = TRUE) )

`summarise()` ungrouping output (override with `.groups` argument)

>

> fig4a <- ggplot( summaryRain,aes(x = rainCat, y = meanN) ) +

+ geom_point(size = 1) +

+ geom_errorbar(aes( ymin = meanN - SE, ymax = meanN + SE), width = 0.5, size = 0.25 ) +

+ theme_bw() +

+ theme( panel.border = element_blank(),

+ panel.grid.major = element_blank(),

+ panel.grid.minor = element_blank(),

+ axis.line = element_line(colour = 'black', size = 0.25),

+ axis.ticks = element_line(colour = "black", size = 0.25),

+ axis.ticks.length=unit(-0.25, "cm"),

+ axis.text.x = element_text(margin=unit(c(0.5,0.5,0.5,0.5), "cm"), size = 10),

+ axis.text.y = element_text(margin=unit(c(0.5,0.5,0.5,0.5), "cm"), size = 10),

+ legend.position="none",

+ axis.title.x=element_text( size = 12 ),

+ axis.title.y=element_text( size = 12 ) ) +

+ labs( x = "Mean rainfall (mm)", y = "Log Weed density") +

+ theme(axis.text.x = element_text(angle = 90))

> fig4a

>

> # Precipitation seasonality

> model2 <- lm( log( Control_Mean + 1) ~ RFCV, data = Open_Data_IC_RC_WD )

> anova(model2)

Analysis of Variance Table

Response: log(Control_Mean + 1)

Df Sum Sq Mean Sq F value Pr(>F)

RFCV 1 40.88 40.884 13.551 0.00025 ***

Residuals 701 2114.90 3.017

---

Signif. codes:

0 ‘***’ 0.001 ‘**’ 0.01 ‘*’ 0.05 ‘.’ 0.1 ‘ ’ 1

> summary(model2)

Call:

lm(formula = log(Control_Mean + 1) ~ RFCV, data = Open_Data_IC_RC_WD)

Residuals:

Min 1Q Median 3Q Max

-3.1511 -1.2844 -0.2399 1.5664 4.9008

Coefficients:

Estimate Std. Error t value Pr(>|t|)

(Intercept) 3.615933 0.155335 23.278 < 2e-16 ***

RFCV -0.007191 0.001953 -3.681 0.00025 ***

---

Signif. codes:

0 ‘***’ 0.001 ‘**’ 0.01 ‘*’ 0.05 ‘.’ 0.1 ‘ ’ 1

Residual standard error: 1.737 on 701 degrees of freedom

Multiple R-squared: 0.01896, Adjusted R-squared: 0.01757

F-statistic: 13.55 on 1 and 701 DF, p-value: 0.00025

>

> Open_Data_IC_WDA <- Open_Data_IC_RC_WD

> Open_Data_IC_WDA$RFCVCat <- round(Open_Data_IC_WDA$ RFCV / 1.5) * 1.5

> summaryRFCV <- Open_Data_IC_WDA %>%

+ group_by( RFCVCat ) %>%

+ summarise( meanN = mean(log( Control_Mean + 1), na.rm = TRUE), SE = stderr(log( Control_Mean + 1), na.rm = TRUE) )

`summarise()` ungrouping output (override with `.groups` argument)

>

> fig4b <- ggplot(summaryRFCV, aes(x = RFCVCat, y = meanN) ) +

+ geom_point(size = 1) +

+ geom_errorbar(aes( ymin = meanN - SE, ymax = meanN + SE), width = 0.5, size = 0.25 ) +

+ theme_bw() +

+ theme( panel.border = element_blank(),

+ panel.grid.major = element_blank(),

+ panel.grid.minor = element_blank(),

+ axis.line = element_line(colour = 'black', size = 0.25),

+ axis.ticks = element_line(colour = "black", size = 0.25),

+ axis.ticks.length=unit(-0.25, "cm"),

+ axis.text.x = element_text(margin=unit(c(0.5,0.5,0.5,0.5), "cm"), size = 10),

+ axis.text.y = element_text(margin=unit(c(0.5,0.5,0.5,0.5), "cm"), size = 10),

+ legend.position="none",

+ axis.title.x=element_text( size = 12 ),

+ axis.title.y=element_text( size = 12 ) ) +

+ labs( x = "Precipitation seasonality (CV)", y = "Log Weed density") +

+ theme(axis.text.x = element_text(angle = 90))

> fig4b

>

> # ------------------------------

>

> # altitude

> model3 <- lm( log( Control_Mean + 1) ~ Alt, data = Open_Data_IC_RC_WD)

> anova(model3)

Analysis of Variance Table

Response: log(Control_Mean + 1)

Df Sum Sq Mean Sq F value Pr(>F)

Alt 1 44.45 44.451 14.759 0.0001333 ***

Residuals 701 2111.33 3.012

---

Signif. codes:

0 ‘***’ 0.001 ‘**’ 0.01 ‘*’ 0.05 ‘.’ 0.1 ‘ ’ 1

> summary(model3)

Call:

lm(formula = log(Control_Mean + 1) ~ Alt, data = Open_Data_IC_RC_WD)

Residuals:

Min 1Q Median 3Q Max

-3.1671 -1.3100 -0.2161 1.5885 4.4729

Coefficients:

Estimate Std. Error t value Pr(>|t|)

(Intercept) 2.6316704 0.1377839 19.100 < 2e-16 ***

Alt 0.0004957 0.0001290 3.842 0.000133 ***

---

Signif. codes:

0 ‘***’ 0.001 ‘**’ 0.01 ‘*’ 0.05 ‘.’ 0.1 ‘ ’ 1

Residual standard error: 1.735 on 701 degrees of freedom

Multiple R-squared: 0.02062, Adjusted R-squared: 0.01922

F-statistic: 14.76 on 1 and 701 DF, p-value: 0.0001333

>

> Open_Data_IC_WDA <- Open_Data_IC_RC_WD

> Open_Data_IC_WDA$altCat <- round(Open_Data_IC_WDA$Alt / 100) * 100

> summaryAlt <- Open_Data_IC_WDA %>%

+ group_by( altCat ) %>%

+ summarise( meanN = mean(log( Control_Mean + 1), na.rm = TRUE), SE = stderr(log( Control_Mean + 1), na.rm = TRUE) )

`summarise()` ungrouping output (override with `.groups` argument)

>

>

>

> fig4c <- ggplot(summaryAlt, aes(x = altCat, y = meanN) ) +

+ geom_point(size = 1) +

+ geom_errorbar(aes( ymin = meanN - SE, ymax = meanN + SE), width = 0.5, size = 0.25 ) +

+ theme_bw() +

+ theme( panel.border = element_blank(),

+ panel.grid.major = element_blank(),

+ panel.grid.minor = element_blank(),

+ axis.line = element_line(colour = 'black', size = 0.25),

+ axis.ticks = element_line(colour = "black", size = 0.25),

+ axis.ticks.length=unit(-0.25, "cm"),

+ axis.text.x = element_text(margin=unit(c(0.5,0.5,0.5,0.5), "cm"), size = 10),

+ axis.text.y = element_text(margin=unit(c(0.5,0.5,0.5,0.5), "cm"), size = 10),

+ legend.position="none",

+ axis.title.x=element_text( size = 12 ),

+ axis.title.y=element_text( size = 12 ) ) +

+ labs( x = "Altitude (m)", y = "Log Weed density") +

+ theme(axis.text.x = element_text(angle = 90))

> fig4c

>

> # Mean temperature

>

> model4 <- lm( log( Control_Mean + 1) ~ Mean_TA, data = Open_Data_IC_RC_WD)

> anova(model4)

Analysis of Variance Table

Response: log(Control_Mean + 1)

Df Sum Sq Mean Sq F value Pr(>F)

Mean_TA 1 1.28 1.2844 0.4179 0.5182

Residuals 701 2154.50 3.0735

> summary(model4)

Call:

lm(formula = log(Control_Mean + 1) ~ Mean_TA, data = Open_Data_IC_RC_WD)

Residuals:

Min 1Q Median 3Q Max

-3.1530 -1.3107 -0.2017 1.5242 4.7070

Coefficients:

Estimate Std. Error t value Pr(>|t|)

(Intercept) 2.77302 0.50619 5.478 6e-08 ***

Mean_TA 0.01469 0.02273 0.646 0.518

---

Signif. codes:

0 ‘***’ 0.001 ‘**’ 0.01 ‘*’ 0.05 ‘.’ 0.1 ‘ ’ 1

Residual standard error: 1.753 on 701 degrees of freedom

Multiple R-squared: 0.0005958, Adjusted R-squared: -0.0008299

F-statistic: 0.4179 on 1 and 701 DF, p-value: 0.5182

>

>

> Open_Data_IC_WDA <- Open_Data_IC_RC_WD

> Open_Data_IC_WDA$tempCat <- round(Open_Data_IC_WDA$Mean_TA / 1) * 1

> summaryTemp <- Open_Data_IC_WDA %>%

+ group_by( tempCat ) %>%

+ summarise( meanN = mean (log( Control_Mean + 1), na.rm = TRUE), SE = stderr(log( Control_Mean + 1), na.rm = TRUE) )

`summarise()` ungrouping output (override with `.groups` argument)

>

>

>

> fig4d <- ggplot(summaryTemp, aes(x = tempCat, y = meanN) ) +

+ geom_point(size = 1) +

+ geom_errorbar(aes( ymin = meanN - SE, ymax = meanN + SE), width = 0.5, size = 0.25 ) +

+ theme_bw() +

+ theme( panel.border = element_blank(),

+ panel.grid.major = element_blank(),

+ panel.grid.minor = element_blank(),

+ axis.line = element_line(colour = 'black', size = 0.25),

+ axis.ticks = element_line(colour = "black", size = 0.25),

+ axis.ticks.length=unit(-0.25, "cm"),

+ axis.text.x = element_text(margin=unit(c(0.5,0.5,0.5,0.5), "cm"), size = 10),

+ axis.text.y = element_text(margin=unit(c(0.5,0.5,0.5,0.5), "cm"), size = 10),

+ legend.position="none",

+ axis.title.x=element_text( size = 12 ),

+ axis.title.y=element_text( size = 12 ) ) +

+ labs( x = "Mean Temperature (\u00B0C)", y = "Log Weed Density") +

+ theme(axis.text.x = element_text(angle = 90))

> fig4d

>

> multiplot(fig4a + labs( tag = "A"), fig4b+ labs( tag = "B"), fig4c+ labs( tag = "C"), fig4d+ labs( tag = "D"), cols = 2)

>

>

>

>

>

>

> #Fig 4a

>

> Open_Data_IC_RC_YD<-read.csv("Open_Data_IC_RC_YD.CSV")

>

> # Mean rainfall

> model1 <- lm (Control_Mean ~ Mean_RF, data = Open_Data_IC_RC_YD )

> anova(model1)

Analysis of Variance Table

Response: Control_Mean

Df Sum Sq Mean Sq F value Pr(>F)

Mean_RF 1 231.7 231.659 6.9962 0.008431 **

Residuals 488 16158.6 33.112

---

Signif. codes:

0 ‘***’ 0.001 ‘**’ 0.01 ‘*’ 0.05 ‘.’ 0.1 ‘ ’ 1

> summary(model1)

Call:

lm(formula = Control_Mean ~ Mean_RF, data = Open_Data_IC_RC_YD)

Residuals:

Min 1Q Median 3Q Max

-4.023 -1.719 -0.940 0.202 38.526

Coefficients:

Estimate Std. Error t value Pr(>|t|)

(Intercept) 5.009795 0.802397 6.244 9.31e-10 ***

Mean_RF -0.022892 0.008655 -2.645 0.00843 **

---

Signif. codes:

0 ‘***’ 0.001 ‘**’ 0.01 ‘*’ 0.05 ‘.’ 0.1 ‘ ’ 1

Residual standard error: 5.754 on 488 degrees of freedom

Multiple R-squared: 0.01413, Adjusted R-squared: 0.01211

F-statistic: 6.996 on 1 and 488 DF, p-value: 0.008431

>

> Open_Data_IC_WDA <- Open_Data_IC_RC_YD

> Open_Data_IC_WDA$rainCat <- round(Open_Data_IC_WDA$ Mean_RF / 1.5) * 1.5

> summaryRain <- Open_Data_IC_WDA %>%

+ group_by( rainCat ) %>%

+ summarise( meanN = mean(Control_Mean , na.rm = TRUE), SE = stderr( Control_Mean , na.rm = TRUE) )

`summarise()` ungrouping output (override with `.groups` argument)

>

> fig4a <- ggplot( summaryRain,aes(x = rainCat, y = meanN) ) +

+ geom_point(size = 1) +

+ geom_errorbar(aes( ymin = meanN - SE, ymax = meanN + SE), width = 0.5, size = 0.25 ) +

+ theme_bw() +

+ theme( panel.border = element_blank(),

+ panel.grid.major = element_blank(),

+ panel.grid.minor = element_blank(),

+ axis.line = element_line(colour = 'black', size = 0.25),

+ axis.ticks = element_line(colour = "black", size = 0.25),

+ axis.ticks.length=unit(-0.25, "cm"),

+ axis.text.x = element_text(margin=unit(c(0.5,0.5,0.5,0.5), "cm"), size = 10),

+ axis.text.y = element_text(margin=unit(c(0.5,0.5,0.5,0.5), "cm"), size = 10),

+ legend.position="none",

+ axis.title.x=element_text( size = 12 ),

+ axis.title.y=element_text( size = 12 ) ) +

+ labs( x = "Mean rainfall (mm)", y = "Yield (T/ha)") +

+ theme(axis.text.x = element_text(angle = 90))

> fig4a

>

>

>

> # Precipitation seasonality

> model2 <- lm( Control_Mean ~ RFCV, data = Open_Data_IC_RC_YD )

> anova(model2)

Analysis of Variance Table

Response: Control_Mean

Df Sum Sq Mean Sq F value Pr(>F)

RFCV 1 155.6 155.564 4.6761 0.03107 *

Residuals 488 16234.7 33.268

---

Signif. codes:

0 ‘***’ 0.001 ‘**’ 0.01 ‘*’ 0.05 ‘.’ 0.1 ‘ ’ 1

> summary(model2)

Call:

lm(formula = Control_Mean ~ RFCV, data = Open_Data_IC_RC_YD)

Residuals:

Min 1Q Median 3Q Max

-3.955 -1.687 -0.851 0.023 38.693

Coefficients:

Estimate Std. Error t value Pr(>|t|)

(Intercept) 1.793042 0.616764 2.907 0.00381 **

RFCV 0.016711 0.007728 2.162 0.03107 *

---

Signif. codes:

0 ‘***’ 0.001 ‘**’ 0.01 ‘*’ 0.05 ‘.’ 0.1 ‘ ’ 1

Residual standard error: 5.768 on 488 degrees of freedom

Multiple R-squared: 0.009491, Adjusted R-squared: 0.007462

F-statistic: 4.676 on 1 and 488 DF, p-value: 0.03107

>

> Open_Data_IC_WDA <- Open_Data_IC_RC_YD

> Open_Data_IC_WDA$RFCVCat <- round(Open_Data_IC_WDA$ RFCV / 1.5) * 1.5

> summaryRFCV <- Open_Data_IC_WDA %>%

+ group_by( RFCVCat ) %>%

+ summarise( meanN = mean( Control_Mean, na.rm = TRUE), SE = stderr(Control_Mean , na.rm = TRUE) )

`summarise()` ungrouping output (override with `.groups` argument)

>

> fig4b <- ggplot(summaryRFCV, aes(x = RFCVCat, y = meanN) ) +

+ geom_point(size = 1) +

+ geom_errorbar(aes( ymin = meanN - SE, ymax = meanN + SE), width = 0.5, size = 0.25 ) +

+ theme_bw() +

+ theme( panel.border = element_blank(),

+ panel.grid.major = element_blank(),

+ panel.grid.minor = element_blank(),

+ axis.line = element_line(colour = 'black', size = 0.25),

+ axis.ticks = element_line(colour = "black", size = 0.25),

+ axis.ticks.length=unit(-0.25, "cm"),

+ axis.text.x = element_text(margin=unit(c(0.5,0.5,0.5,0.5), "cm"), size = 10),

+ axis.text.y = element_text(margin=unit(c(0.5,0.5,0.5,0.5), "cm"), size = 10),

+ legend.position="none",

+ axis.title.x=element_text( size = 12 ),

+ axis.title.y=element_text( size = 12 ) ) +

+ labs( x = "Precipitation seasonality (CV)", y = "Yield (T/ha)") +

+ theme(axis.text.x = element_text(angle = 90))

> fig4b

>

> # ------------------------------

>

> # altitude

> model3 <- lm( Control_Mean ~ Alt, data = Open_Data_IC_RC_YD)

> anova(model3)

Analysis of Variance Table

Response: Control_Mean

Df Sum Sq Mean Sq F value Pr(>F)

Alt 1 223.7 223.730 6.7535 0.00964 **

Residuals 488 16166.5 33.128

---

Signif. codes:

0 ‘***’ 0.001 ‘**’ 0.01 ‘*’ 0.05 ‘.’ 0.1 ‘ ’ 1

> summary(model3)

Call:

lm(formula = Control_Mean ~ Alt, data = Open_Data_IC_RC_YD)

Residuals:

Min 1Q Median 3Q Max

-5.040 -1.679 -1.148 -0.346 38.260

Coefficients:

Estimate Std. Error t value Pr(>|t|)

(Intercept) 1.5156081 0.6282540 2.412 0.01622 *

Alt 0.0014442 0.0005557 2.599 0.00964 **

---

Signif. codes:

0 ‘***’ 0.001 ‘**’ 0.01 ‘*’ 0.05 ‘.’ 0.1 ‘ ’ 1

Residual standard error: 5.756 on 488 degrees of freedom

Multiple R-squared: 0.01365, Adjusted R-squared: 0.01163

F-statistic: 6.753 on 1 and 488 DF, p-value: 0.00964

>

> Open_Data_IC_WDA <- Open_Data_IC_RC_YD

> Open_Data_IC_WDA$altCat <- round(Open_Data_IC_WDA$Alt / 100) * 100

> summaryAlt <- Open_Data_IC_WDA %>%

+ group_by( altCat ) %>%

+ summarise( meanN = mean( Control_Mean, na.rm = TRUE), SE = stderr(Control_Mean , na.rm = TRUE) )

`summarise()` ungrouping output (override with `.groups` argument)

>

> fig4c <- ggplot(summaryAlt, aes(x = altCat, y = meanN) ) +

+ geom_point(size = 1) +

+ geom_errorbar(aes( ymin = meanN - SE, ymax = meanN + SE), width = 0.5, size = 0.25 ) +

+ theme_bw() +

+ theme( panel.border = element_blank(),

+ panel.grid.major = element_blank(),

+ panel.grid.minor = element_blank(),

+ axis.line = element_line(colour = 'black', size = 0.25),

+ axis.ticks = element_line(colour = "black", size = 0.25),

+ axis.ticks.length=unit(-0.25, "cm"),

+ axis.text.x = element_text(margin=unit(c(0.5,0.5,0.5,0.5), "cm"), size = 10),

+ axis.text.y = element_text(margin=unit(c(0.5,0.5,0.5,0.5), "cm"), size = 10),

+ legend.position="none",

+ axis.title.x=element_text( size = 12 ),

+ axis.title.y=element_text( size = 12 ) ) +

+ labs( x = "Altitude (m)", y = "Yield (T/ha)") +

+ theme(axis.text.x = element_text(angle = 90))

> fig4c

>

> # Mean temperature

>

> model4 <- lm( Control_Mean ~ Mean_TA, data = Open_Data_IC_RC_YD)

> anova(model4)

Analysis of Variance Table

Response: Control_Mean

Df Sum Sq Mean Sq F value Pr(>F)

Mean_TA 1 471.5 471.46 14.453 0.0001619 ***

Residuals 488 15918.8 32.62

---

Signif. codes:

0 ‘***’ 0.001 ‘**’ 0.01 ‘*’ 0.05 ‘.’ 0.1 ‘ ’ 1

> summary(model4)

Call:

lm(formula = Control_Mean ~ Mean_TA, data = Open_Data_IC_RC_YD)

Residuals:

Min 1Q Median 3Q Max

-5.034 -1.626 -0.926 -0.054 38.097

Coefficients:

Estimate Std. Error t value Pr(>|t|)

(Intercept) 10.739 2.051 5.235 2.46e-07 ***

Mean_TA -0.346 0.091 -3.802 0.000162 ***

---

Signif. codes:

0 ‘***’ 0.001 ‘**’ 0.01 ‘*’ 0.05 ‘.’ 0.1 ‘ ’ 1

Residual standard error: 5.711 on 488 degrees of freedom

Multiple R-squared: 0.02876, Adjusted R-squared: 0.02677

F-statistic: 14.45 on 1 and 488 DF, p-value: 0.0001619

>

> Open_Data_IC_WDA <- Open_Data_IC_RC_YD

> Open_Data_IC_WDA$tempCat <- round(Open_Data_IC_WDA$Mean_TA / 1) * 1

> summaryTemp <- Open_Data_IC_WDA %>%

+ group_by( tempCat ) %>%

+ summarise( meanN = mean( Control_Mean, na.rm = TRUE), SE = stderr(Control_Mean , na.rm = TRUE) )

`summarise()` ungrouping output (override with `.groups` argument)

>

> fig4d <- ggplot(summaryTemp, aes(x = tempCat, y = meanN) ) +

+ geom_point(size = 1) +

+ geom_errorbar(aes( ymin = meanN - SE, ymax = meanN + SE), width = 0.5, size = 0.25 ) +

+ theme_bw() +

+ theme( panel.border = element_blank(),

+ panel.grid.major = element_blank(),

+ panel.grid.minor = element_blank(),

+ axis.line = element_line(colour = 'black', size = 0.25),

+ axis.ticks = element_line(colour = "black", size = 0.25),

+ axis.ticks.length=unit(-0.25, "cm"),

+ axis.text.x = element_text(margin=unit(c(0.5,0.5,0.5,0.5), "cm"), size = 10),

+ axis.text.y = element_text(margin=unit(c(0.5,0.5,0.5,0.5), "cm"), size = 10),

+ legend.position="none",

+ axis.title.x=element_text( size = 12 ),

+ axis.title.y=element_text( size = 12 ) ) +

+ labs( x = "Mean Temperature (\u00B0C)", y = "Yield (T/ha)") +

+ theme(axis.text.x = element_text(angle = 90))

> fig4d

>

> multiplot(fig4a + labs( tag = "A"), fig4b+ labs( tag = "B"), fig4c+ labs( tag = "C"), fig4d+ labs( tag = "D"), cols = 2)

>

> Linear Model for Diversity and plots

Error: unexpected symbol in "Linear Model"

>

> MST_RC_ASD_IMP_WD<-read.csv("MST_RC_ASD_IMP_WD.CSV")

>

> stderr <- function(x) sd(x) / sqrt(length(x))

>

> MST_RC_ASD_IMP_WD$DIV <- as.factor(MST_RC_ASD_IMP_WD$DIV)#To change DIV to 4 level factor

>

> #Divide treatment by control to make weed density % difference

> MST_RC_ASD_IMP_WD$WDDif<- (MST_RC_ASD_IMP_WD$Treat_Mean/MST_RC_ASD_IMP_WD$Control_Mean)*100

>

> #Look at diversity and change in weed density

> LM1 <- lm( WDDif ~ DIV, data=MST_RC_ASD_IMP_WD)

> anova(LM1)

Analysis of Variance Table

Response: WDDif

Df Sum Sq Mean Sq F value Pr(>F)

DIV 3 18175 6058 0.1363 0.9383

Residuals 365 16219701 44438

>

> summary(LM1)

Call:

lm(formula = WDDif ~ DIV, data = MST_RC_ASD_IMP_WD)

Residuals:

Min 1Q Median 3Q Max

-93.61 -63.61 -33.61 3.46 2033.66

Coefficients:

Estimate Std. Error t value Pr(>|t|)

(Intercept) 80.15 74.53 1.075 0.283

DIV2 13.46 75.40 0.179 0.858

DIV3 -17.81 93.43 -0.191 0.849

DIV4 -15.98 120.18 -0.133 0.894

Residual standard error: 210.8 on 365 degrees of freedom

Multiple R-squared: 0.001119, Adjusted R-squared: -0.007091

F-statistic: 0.1363 on 3 and 365 DF, p-value: 0.9383

>

> coeffs <- data.frame( summary(LM1)$coefficients )

>

> coeffs$names <- str_remove( rownames(coeffs), "MST_RC_ASD_IMP_WD" )

>

> RCD<- c("1", "2", "3","4")#For the x tick labels

>

> fig5a <- ggplot(coeffs, aes(x = names,Estimate, y = Estimate) ) +

+ geom_point(size = 1) +

+ geom_errorbar(aes( ymin = Estimate - Std..Error, ymax =Estimate + Std..Error ), width = 0.2, size = 0.25 ) +

+ theme_bw() + scale_x_discrete(labels= RCD)+

+ theme( panel.border = element_blank(),

+ panel.grid.major = element_blank(),

+ panel.grid.minor = element_blank(),

+ axis.line = element_line(colour = 'black', size = 0.25),

+ axis.ticks = element_line(colour = "black", size = 0.25),

+ axis.ticks.length=unit(-0.25, "cm"),

+ axis.text.x = element_text(margin=unit(c(0.5,0.5,0.5,0.5), "cm"), size = 10),

+ axis.text.y = element_text(margin=unit(c(0.5,0.5,0.5,0.5), "cm"), size = 8),

+ legend.position="none",

+ axis.title.x=element_text( size = 12 ),

+ axis.title.y=element_text( size = 12 ) ) +

+ labs( x = "Rotation Crop Diversity", y = "Density Change Coefficient") +

+ theme(axis.text.x = element_text(angle = 0, vjust = .7, hjust=.65))

> fig5a

>

> fig5b<-ggplot(data = MST_RC_ASD_IMP_WD, aes(x=DIV, y=WDDif)) +

+ geom_boxplot(fill=c('red', 'Yellow', 'blue','green'))+

+ labs( x = "Rotation Crop Diversity", y = "Weed Density Change")

>

>

> fig5b

>

> #Redo the LMER with diversity as a factor using effect size

>

> mixed.mod1 <- lmer(HEDGES ~

+ DIV +

+ (1|Study_ID) ,

+ data=MST_RC_ASD_IMP_WD,

+ weights = 1/VAR_G,

+ na.action = "na.omit")

> anova(mixed.mod1)

Type III Analysis of Variance Table with Satterthwaite's method

Sum Sq Mean Sq NumDF DenDF F value Pr(>F)

DIV 4.9463 1.6488 3 61.336 0.4474 0.72

> summary(mixed.mod1)

Linear mixed model fit by REML. t-tests use

Satterthwaite's method [lmerModLmerTest]

Formula: HEDGES ~ DIV + (1 | Study_ID)

Data: MST_RC_ASD_IMP_WD

Weights: 1/VAR_G

REML criterion at convergence: 1258

Scaled residuals:

Min 1Q Median 3Q Max

-3.3679 -0.4145 0.0830 0.7601 3.1894

Random effects:

Groups Name Variance Std.Dev.

Study_ID (Intercept) 0.971 0.9854

Residual 3.685 1.9197

Number of obs: 368, groups: Study_ID, 29

Fixed effects:

Estimate Std. Error df t value

(Intercept) 0.5271 1.0867 21.6851 0.485

DIV2 0.4585 1.1068 21.6969 0.414

DIV3 -0.0798 1.2123 27.6950 -0.066

DIV4 0.3431 1.2766 34.5926 0.269

Pr(>|t|)

(Intercept) 0.632

DIV2 0.683

DIV3 0.948

DIV4 0.790

Correlation of Fixed Effects:

(Intr) DIV2 DIV3

DIV2 -0.982

DIV3 -0.896 0.899

DIV4 -0.851 0.854 0.884

>

> coeffs <- data.frame( summary(mixed.mod1)$coefficients )

>

> coeffs$names <- str_remove( rownames(coeffs), "MST_RC_ASD_IMP_WD" )

>

> fig5c <- ggplot(coeffs, aes(x = names,Estimate, y = Estimate) ) +

+ geom_point(size = 1) +

+ geom_errorbar(aes( ymin = Estimate - Std..Error, ymax =Estimate + Std..Error ), width = 0.2, size = 0.25 ) +

+ theme_bw() + scale_x_discrete(labels= RCD)+

+ theme( panel.border = element_blank(),

+ panel.grid.major = element_blank(),

+ panel.grid.minor = element_blank(),

+ axis.line = element_line(colour = 'black', size = 0.25),

+ axis.ticks = element_line(colour = "black", size = 0.25),

+ axis.ticks.length=unit(-0.25, "cm"),

+ axis.text.x = element_text(margin=unit(c(0.5,0.5,0.5,0.5), "cm"), size = 10),

+ axis.text.y = element_text(margin=unit(c(0.5,0.5,0.5,0.5), "cm"), size = 8),

+ legend.position="none",

+ axis.title.x=element_text( size = 12 ),

+ axis.title.y=element_text( size = 12 ) ) +

+ labs( x = "Rotation Crop Diversity", y = "Effect Size (g)") +

+ theme(axis.text.x = element_text(angle = 0, vjust = .7, hjust=.65))

>

> fig5c

>

> fig5d<-ggplot(data = MST_RC_ASD_IMP_WD, aes(x=DIV, y=HEDGES)) +

+ geom_boxplot(fill=c('grey', 'grey', 'grey','grey'))+

+ labs( x = "Rotation Crop Diversity", y = "Effect Size (g)")

>

>

> fig5d

Warning message:

Removed 1 rows containing non-finite values

(stat_boxplot).

>

>

>

> Figure5e <- ggplot( MST_RC_ASD_IMP_WD, aes(x = DIV, y = HEDGES) ) +

+ geom_point( size = 1) +

+ geom_errorbar( aes(ymin = HEDGES - VAR_G, ymax = HEDGES + VAR_G, width = 0.1, )) +

+ theme_bw() +

+ theme( panel.border = element_blank(),

+ panel.grid.major = element_blank(),

+ panel.grid.minor = element_blank(),

+ legend.position="none",

+ axis.line = element_line(colour = 'black', size = 0.25),

+ axis.ticks = element_line(colour = "black", size = 0.25),

+ axis.text.x = element_text(size = 10),

+ axis.text.y = element_text(size = 8),

+ axis.title.x=element_text(size = 14),

+ axis.title.y=element_text(size = 14) ) +

+ geom_hline(yintercept = 0, linetype = "dashed") +

+ labs(x = "Rotation Crop Diversity") + labs( y = "Effect Size (g)", las=2)

>

> Figure5e

Warning message:

Removed 1 rows containing missing values

(geom_point).

>

> multiplot(fig5a + labs( tag = "A"), fig5c+ labs( tag

+
