# Supplementary material for: Crop diversification and parasitic weed abundance: a global meta-analysis
Source: Sci Rep. 2022 Nov 12;12:19413. doi: 10.1038/s41598-022-24047-2 (PMC9653488; doi:10.1038/s41598-022-24047-2)
Supplement: Supplementary file 14 — Supplementary Information 14. [file 41598_2022_24047_MOESM14_ESM.docx]

# Model 1

## (Intercrop Weed Density “G” ~ Weed Species + Host Crop Species + Intercrop Species)

> MST_IC_ASD_IMP_WD<-read.csv("MST_IC_ASD_IMP_WD.CSV")

>

> mixed.mod1 <- lmer(HEDGES ~ # this is the individual effect size as the response variable

+ W_SP+HC_SP+IC_SP+ # this is the fixed effects - so you could add the grouping variable here and it will tell you whether there is a difference in

+ # effect size between different levels of the variable

+ (1|Study_ID) , # this is the random effects (in this case grouping multiple effect sizes in each study together)

+ data=MST_IC_ASD_IMP_WD,

+ weights = 1/VAR_G, # this is the weighting variable required for a meta-analysis (var.g = the individual effect size variances)

+ na.action = "na.omit")

fixed-effect model matrix is rank deficient so dropping 7 columns / coefficients

> anova(mixed.mod1)

> summary(mixed.mod1)

# Model 2

## (Intercrop Weed Density “G” ~ Host Crop Variety + Intercrop Variety)

MST_IC_ASD_IMP_WD<-read.csv("MST_IC_ASD_IMP_WD.CSV")

mixed.mod2 <- lmer(HEDGES ~

HC_V + IC_V+

(1|Study_ID) ,

data=MST_IC_ASD_IMP_WD,

weights = 1/VAR_G,

na.action = "na.omit")

anova(mixed.mod2)

summary(mixed.mod2)

# Model 3

## (Intercrop Yield “G” ~ Weed Species + Host Crop Species + Intercrop Species)

mixed.mod3 <- lmer(HEDGES ~

HC_SP+ IC_SP+ W_SP+

(1|Study_ID) ,

data=MST_IC_ASD_IMP_YD,

weights = 1/VAR_G,

na.action = "na.omit")

anova(mixed.mod3)

summary(mixed.mod3)

# Model 4

## (Intercrop Yield “G” ~ Host Crop Variety + Intercrop Variety)

MST_IC_ASD_IMP_YD<-read.csv("MST_IC_ASD_IMP_YD.CSV")

mixed.mod4 <- lmer(HEDGES ~

HC_V + IC_V+

(1|Study_ID) ,

data=MST_IC_ASD_IMP_YD,

weights = 1/VAR_G,

na.action = "na.omit")

anova(mixed.mod4)

summary(mixed.mod4)

# Model 5

(Rotation crop Weed Density “G” ~ Weed Species + Host Crop Species + Rotation crop Species 1)

mixed.mod5 <- lmer(HEDGES ~

W_SP+ HC_SP+ RC_1_SP+

(1|Study_ID) ,

data=MST_RC_ASD_IMP_WD,

weights = 1/VAR_G,

na.action = "na.omit")

anova(mixed.mod5)

summary(mixed.mod5)

# Model 6

(Rotation crop Weed Density “G” ~ Host Crop Variety + Rotation Crop 1 Variety)

MST_RC_ASD_IMP_WD<-read.csv("MST_RC_ASD_IMP_WD.CSV")

mixed.mod6 <- lmer(HEDGES ~

HC_V+ RC_1_V+

(1|Study_ID) ,

data=MST_RC_ASD_IMP_WD,

weights = 1/VAR_G,

na.action = "na.omit")

anova(mixed.mod6)

summary(mixed.mod6)

# Model 7

(Rotation crop Yield “G” ~ Weed Species + Host Crop Species + Rotation crop 1 Species)

MST_RC_ASD_IMP_YD<-read.csv("MST_RC_ASD_IMP_YD.CSV")

mixed.mod7 <- lmer(HEDGES ~

W_SP + HC_SP + RC_1_SP+

(1|Study_ID) ,

data=MST_RC_ASD_IMP_YD,

weights = 1/VAR_G,

na.action = "na.omit")

anova(mixed.mod7)

summary(mixed.mod7)

# Model 8

(Rotation crop Yield “G” ~ Host Crop Variety + Rotation Crop 1 Variety)

rm(list=ls())

library(lme4)

library(lmerTest)

setwd("C:/Users/Ragenaky/Desktop/Thesis chapter 3/Data/Master Sheets")

MST_RC_ASD_IMP_YD<-read.csv("MST_RC_ASD_IMP_YD.CSV")

mixed.mod8 <- lmer(HEDGES ~

HC_V + RC_1_V+

(1|Study_ID) ,

data=MST_RC_ASD_IMP_YD,

weights = 1/VAR_G,

na.action = "na.omit")

anova(mixed.mod8)

summary(mixed.mod8)

# Model 9

# MST_IC_ASD_IMP_WD<-read.csv("MST_IC_ASD_IMP_WD.CSV")

# mixed.mod9 <- lmer(Control_Mean ~

# Treat_Mean +

# (1|Study_ID),

# data=MST_IC_ASD_IMP_WD,weights = 1/VAR_G, # this is the weighting variable required for a meta-analysis (var.g = the individual effect size variances)

# na.action = "na.omit")

# anova(mixed.mod9)

# summary(mixed.mod9)

# Model 10

# MST_IC_ASD_IMP_YD<-read.csv("MST_IC_ASD_IMP_YD.CSV")

# mixed.mod10 <- lmer(Control_Mean ~

# Treat_Mean +

# (1|Study_ID),

# data=MST_IC_ASD_IMP_YD,weights = 1/VAR_G, # this is the weighting variable required for a meta-analysis (var.g = the individual effect size variances)

# na.action = "na.omit")

# anova(mixed.mod10)

# summary(mixed.mod10)

# Model 11

MST_RC_ASD_IMP_WD<-read.csv("MST_RC_ASD_IMP_WD.CSV")

mixed.mod11 <- lmer(Control_Mean ~

Treat_Mean +

(1|Study_ID),

data=MST_RC_ASD_IMP_WD,weights = 1/VAR_G, # this is the weighting variable required for a meta-analysis (var.g = the individual effect size variances)

na.action = "na.omit")

anova(mixed.mod11)

summary(mixed.mod11)

# Model 12

MST_RC_ASD_IMP_YD<-read.csv("MST_RC_ASD_IMP_YD.CSV")

mixed.mod12 <- lmer(Control_Mean ~

Treat_Mean +

(1|Study_ID),

data=MST_RC_ASD_IMP_YD, weights = 1/VAR_G, # this is the weighting variable required for a meta-analysis (var.g = the individual effect size variances)

na.action = "na.omit")

anova(mixed.mod12)

summary(mixed.mod12)

# Diversity Models

# Model 13

# #Divide treatment by control to make weed density % difference

# MST_RC_ASD_IMP_WD$WDDif<- (MST_RC_ASD_IMP_WD$Treat_Mean/MST_RC_ASD_IMP_WD$Control_Mean)*100

# #Look at diversity and change in weed density

# mixed.mod13 <- lmer( WDDif ~ DIV + (1|Study_ID),data=MST_RC_ASD_IMP_WD,weights = 1/VAR_G, # this is the weighting variable required for a meta-analysis (var.g = the individual effect size variances)

# na.action = "na.omit")

# anova(mixed.mod13)

# summary(mixed.mod13)

# Model 14

# mixed.mod14 <- lmer(HEDGES ~

# DIV +

# (1|Study_ID) ,

# data=MST_RC_ASD_IMP_WD,

# weights = 1/VAR_G,

# na.action = "na.omit")

# anova(mixed.mod14)

# summary(mixed.mod14)

# Model 15

# MST_RC_ASD_IMP_YD<-read.csv("MST_RC_ASD_IMP_YD.CSV")

# mixed.mod15 <- lmer(HEDGES ~

# DIV +

# (1|Study_ID) ,

# data=MST_RC_ASD_IMP_YD,

# weights = 1/VAR_G,

# na.action = "na.omit")

# anova(mixed.mod15)

# summary(mixed.mod15)

# Climate v Weed Density

rm(list=ls())# wipes slate clean

library(mgcv)

library(lme4)

library(lmerTest)

library(ggplot2)

library(dplyr)

library( geosphere )

library( stringr)

#Calculate a standard error

stderr <- function(x, ...) sd(x, na.rm = TRUE) / sqrt(length(is.na(x == FALSE)) )

### Install this When you start for Multiplots!!!#####

#

# ggplot objects can be passed in ..., or to plotlist (as a list of ggplot objects)

# - cols: Number of columns in layout

# - layout: A matrix specifying the layout. If present, 'cols' is ignored.

#

# If the layout is something like matrix(c(1,2,3,3), nrow=2, byrow=TRUE),

# then plot 1 will go in the upper left, 2 will go in the upper right, and

# 3 will go all the way across the bottom.

#

multiplot <- function(..., plotlist=NULL, file, cols=1, layout=NULL) {

library(grid)

# Make a list from the ... arguments and plotlist

plots <- c(list(...), plotlist)

numPlots = length(plots)

# If layout is NULL, then use 'cols' to determine layout

if (is.null(layout)) {

# Make the panel

# ncol: Number of columns of plots

# nrow: Number of rows needed, calculated from # of cols

layout <- matrix(seq(1, cols * ceiling(numPlots/cols)),

ncol = cols, nrow = ceiling(numPlots/cols))

}

if (numPlots==1) {

print(plots[[1]])

} else {

# Set up the page

grid.newpage()

pushViewport(viewport(layout = grid.layout(nrow(layout), ncol(layout))))

# Make each plot, in the correct location

for (i in 1:numPlots) {

# Get the i,j matrix positions of the regions that contain this subplot

matchidx <- as.data.frame(which(layout == i, arr.ind = TRUE))

print(plots[[i]], vp = viewport(layout.pos.row = matchidx$row,

layout.pos.col = matchidx$col))

}

}

}

#Fig 4a

Open_Data_IC_RC_WD<-read.csv("Open_Data_IC_RC_WD.CSV")

# Mean rainfall

model1 <- lm( log( Control_Mean + 1) ~ Mean_RF, data = Open_Data_IC_RC_WD )

anova(model1)

summary(model1)

Open_Data_IC_WDA <- Open_Data_IC_RC_WD

Open_Data_IC_WDA$rainCat <- round(Open_Data_IC_WDA$ Mean_RF / 1.5) * 1.5

summaryRain <- Open_Data_IC_WDA %>%

group_by( rainCat ) %>%

summarise( meanN = mean(log( Control_Mean + 1), na.rm = TRUE), SE = stderr(log( Control_Mean + 1), na.rm = TRUE) )

fig4a <- ggplot( summaryRain,aes(x = rainCat, y = meanN) ) +

geom_point(size = 1) +

geom_errorbar(aes( ymin = meanN - SE, ymax = meanN + SE), width = 0.5, size = 0.25 ) +

theme_bw() +

theme( panel.border = element_blank(),

panel.grid.major = element_blank(),

panel.grid.minor = element_blank(),

axis.line = element_line(colour = 'black', size = 0.25),

axis.ticks = element_line(colour = "black", size = 0.25),

axis.ticks.length=unit(-0.25, "cm"),

axis.text.x = element_text(margin=unit(c(0.5,0.5,0.5,0.5), "cm"), size = 10),

axis.text.y = element_text(margin=unit(c(0.5,0.5,0.5,0.5), "cm"), size = 10),

legend.position="none",

axis.title.x=element_text( size = 12 ),

axis.title.y=element_text( size = 12 ) ) +

labs( x = "Mean rainfall (mm)", y = "Log Weed density") +

theme(axis.text.x = element_text(angle = 90))

fig4a

# Precipitation seasonality

model2 <- lm( log( Control_Mean + 1) ~ RFCV, data = Open_Data_IC_RC_WD )

anova(model2)

summary(model2)

Open_Data_IC_WDA <- Open_Data_IC_RC_WD

Open_Data_IC_WDA$RFCVCat <- round(Open_Data_IC_WDA$ RFCV / 1.5) * 1.5

summaryRFCV <- Open_Data_IC_WDA %>%

group_by( RFCVCat ) %>%

summarise( meanN = mean(log( Control_Mean + 1), na.rm = TRUE), SE = stderr(log( Control_Mean + 1), na.rm = TRUE) )

fig4b <- ggplot(summaryRFCV, aes(x = RFCVCat, y = meanN) ) +

geom_point(size = 1) +

geom_errorbar(aes( ymin = meanN - SE, ymax = meanN + SE), width = 0.5, size = 0.25 ) +

theme_bw() +

theme( panel.border = element_blank(),

panel.grid.major = element_blank(),

panel.grid.minor = element_blank(),

axis.line = element_line(colour = 'black', size = 0.25),

axis.ticks = element_line(colour = "black", size = 0.25),

axis.ticks.length=unit(-0.25, "cm"),

axis.text.x = element_text(margin=unit(c(0.5,0.5,0.5,0.5), "cm"), size = 10),

axis.text.y = element_text(margin=unit(c(0.5,0.5,0.5,0.5), "cm"), size = 10),

legend.position="none",

axis.title.x=element_text( size = 12 ),

axis.title.y=element_text( size = 12 ) ) +

labs( x = "Precipitation seasonality (CV)", y = "Log Weed density") +

theme(axis.text.x = element_text(angle = 90))

fig4b

# ------------------------------

# altitude

model3 <- lm( log( Control_Mean + 1) ~ Alt, data = Open_Data_IC_RC_WD)

anova(model3)

summary(model3)

Open_Data_IC_WDA <- Open_Data_IC_RC_WD

Open_Data_IC_WDA$altCat <- round(Open_Data_IC_WDA$Alt / 100) * 100

summaryAlt <- Open_Data_IC_WDA %>%

group_by( altCat ) %>%

summarise( meanN = mean(log( Control_Mean + 1), na.rm = TRUE), SE = stderr(log( Control_Mean + 1), na.rm = TRUE) )

fig4c <- ggplot(summaryAlt, aes(x = altCat, y = meanN) ) +

geom_point(size = 1) +

geom_errorbar(aes( ymin = meanN - SE, ymax = meanN + SE), width = 0.5, size = 0.25 ) +

theme_bw() +

theme( panel.border = element_blank(),

panel.grid.major = element_blank(),

panel.grid.minor = element_blank(),

axis.line = element_line(colour = 'black', size = 0.25),

axis.ticks = element_line(colour = "black", size = 0.25),

axis.ticks.length=unit(-0.25, "cm"),

axis.text.x = element_text(margin=unit(c(0.5,0.5,0.5,0.5), "cm"), size = 10),

axis.text.y = element_text(margin=unit(c(0.5,0.5,0.5,0.5), "cm"), size = 10),

legend.position="none",

axis.title.x=element_text( size = 12 ),

axis.title.y=element_text( size = 12 ) ) +

labs( x = "Altitude (m)", y = "Log Weed density") +

theme(axis.text.x = element_text(angle = 90))

fig4c

# Mean temperature

model4 <- lm( log( Control_Mean + 1) ~ Mean_TA, data = Open_Data_IC_RC_WD)

anova(model4)

summary(model4)

Open_Data_IC_WDA <- Open_Data_IC_RC_WD

Open_Data_IC_WDA$tempCat <- round(Open_Data_IC_WDA$Mean_TA / 1) * 1

summaryTemp <- Open_Data_IC_WDA %>%

group_by( tempCat ) %>%

summarise( meanN = mean (log( Control_Mean + 1), na.rm = TRUE), SE = stderr(log( Control_Mean + 1), na.rm = TRUE) )

fig4d <- ggplot(summaryTemp, aes(x = tempCat, y = meanN) ) +

geom_point(size = 1) +

geom_errorbar(aes( ymin = meanN - SE, ymax = meanN + SE), width = 0.5, size = 0.25 ) +

theme_bw() +

theme( panel.border = element_blank(),

panel.grid.major = element_blank(),

panel.grid.minor = element_blank(),

axis.line = element_line(colour = 'black', size = 0.25),

axis.ticks = element_line(colour = "black", size = 0.25),

axis.ticks.length=unit(-0.25, "cm"),

axis.text.x = element_text(margin=unit(c(0.5,0.5,0.5,0.5), "cm"), size = 10),

axis.text.y = element_text(margin=unit(c(0.5,0.5,0.5,0.5), "cm"), size = 10),

legend.position="none",

axis.title.x=element_text( size = 12 ),

axis.title.y=element_text( size = 12 ) ) +

labs( x = "Mean Temperature (\u00B0C)", y = "Log Weed Density") +

theme(axis.text.x = element_text(angle = 90))

fig4d

multiplot(fig4a + labs( tag = "A"), fig4b+ labs( tag = "B"), fig4c+ labs( tag = "C"), fig4d+ labs( tag = "D"), cols = 2)

# Climate v Yield

#Fig 4a

Open_Data_IC_RC_YD<-read.csv("Open_Data_IC_RC_YD.CSV")

# Mean rainfall

model1 <- lm (Control_Mean ~ Mean_RF, data = Open_Data_IC_RC_YD )

anova(model1)

summary(model1)

Open_Data_IC_WDA <- Open_Data_IC_RC_YD

Open_Data_IC_WDA$rainCat <- round(Open_Data_IC_WDA$ Mean_RF / 1.5) * 1.5

summaryRain <- Open_Data_IC_WDA %>%

group_by( rainCat ) %>%

summarise( meanN = mean(Control_Mean , na.rm = TRUE), SE = stderr( Control_Mean , na.rm = TRUE) )

fig4a <- ggplot( summaryRain,aes(x = rainCat, y = meanN) ) +

geom_point(size = 1) +

geom_errorbar(aes( ymin = meanN - SE, ymax = meanN + SE), width = 0.5, size = 0.25 ) +

theme_bw() +

theme( panel.border = element_blank(),

panel.grid.major = element_blank(),

panel.grid.minor = element_blank(),

axis.line = element_line(colour = 'black', size = 0.25),

axis.ticks = element_line(colour = "black", size = 0.25),

axis.ticks.length=unit(-0.25, "cm"),

axis.text.x = element_text(margin=unit(c(0.5,0.5,0.5,0.5), "cm"), size = 10),

axis.text.y = element_text(margin=unit(c(0.5,0.5,0.5,0.5), "cm"), size = 10),

legend.position="none",

axis.title.x=element_text( size = 12 ),

axis.title.y=element_text( size = 12 ) ) +

labs( x = "Mean rainfall (mm)", y = "Yield (T/ha)") +

theme(axis.text.x = element_text(angle = 90))

fig4a

# Precipitation seasonality

model2 <- lm( Control_Mean ~ RFCV, data = Open_Data_IC_RC_YD )

anova(model2)

summary(model2)

Open_Data_IC_WDA <- Open_Data_IC_RC_YD

Open_Data_IC_WDA$RFCVCat <- round(Open_Data_IC_WDA$ RFCV / 1.5) * 1.5

summaryRFCV <- Open_Data_IC_WDA %>%

group_by( RFCVCat ) %>%

summarise( meanN = mean( Control_Mean, na.rm = TRUE), SE = stderr(Control_Mean , na.rm = TRUE) )

fig4b <- ggplot(summaryRFCV, aes(x = RFCVCat, y = meanN) ) +

geom_point(size = 1) +

geom_errorbar(aes( ymin = meanN - SE, ymax = meanN + SE), width = 0.5, size = 0.25 ) +

theme_bw() +

theme( panel.border = element_blank(),

panel.grid.major = element_blank(),

panel.grid.minor = element_blank(),

axis.line = element_line(colour = 'black', size = 0.25),

axis.ticks = element_line(colour = "black", size = 0.25),

axis.ticks.length=unit(-0.25, "cm"),

axis.text.x = element_text(margin=unit(c(0.5,0.5,0.5,0.5), "cm"), size = 10),

axis.text.y = element_text(margin=unit(c(0.5,0.5,0.5,0.5), "cm"), size = 10),

legend.position="none",

axis.title.x=element_text( size = 12 ),

axis.title.y=element_text( size = 12 ) ) +

labs( x = "Precipitation seasonality (CV)", y = "Yield (T/ha)") +

theme(axis.text.x = element_text(angle = 90))

fig4b

# ------------------------------

# altitude

model3 <- lm( Control_Mean ~ Alt, data = Open_Data_IC_RC_YD)

anova(model3)

summary(model3)

Open_Data_IC_WDA <- Open_Data_IC_RC_YD

Open_Data_IC_WDA$altCat <- round(Open_Data_IC_WDA$Alt / 100) * 100

summaryAlt <- Open_Data_IC_WDA %>%

group_by( altCat ) %>%

summarise( meanN = mean( Control_Mean, na.rm = TRUE), SE = stderr(Control_Mean , na.rm = TRUE) )

fig4c <- ggplot(summaryAlt, aes(x = altCat, y = meanN) ) +

geom_point(size = 1) +

geom_errorbar(aes( ymin = meanN - SE, ymax = meanN + SE), width = 0.5, size = 0.25 ) +

theme_bw() +

theme( panel.border = element_blank(),

panel.grid.major = element_blank(),

panel.grid.minor = element_blank(),

axis.line = element_line(colour = 'black', size = 0.25),

axis.ticks = element_line(colour = "black", size = 0.25),

axis.ticks.length=unit(-0.25, "cm"),

axis.text.x = element_text(margin=unit(c(0.5,0.5,0.5,0.5), "cm"), size = 10),

axis.text.y = element_text(margin=unit(c(0.5,0.5,0.5,0.5), "cm"), size = 10),

legend.position="none",

axis.title.x=element_text( size = 12 ),

axis.title.y=element_text( size = 12 ) ) +

labs( x = "Altitude (m)", y = "Yield (T/ha)") +

theme(axis.text.x = element_text(angle = 90))

fig4c

# Mean temperature

model4 <- lm( Control_Mean ~ Mean_TA, data = Open_Data_IC_RC_YD)

anova(model4)

summary(model4)

Open_Data_IC_WDA <- Open_Data_IC_RC_YD

Open_Data_IC_WDA$tempCat <- round(Open_Data_IC_WDA$Mean_TA / 1) * 1

summaryTemp <- Open_Data_IC_WDA %>%

group_by( tempCat ) %>%

summarise( meanN = mean( Control_Mean, na.rm = TRUE), SE = stderr(Control_Mean , na.rm = TRUE) )

fig4d <- ggplot(summaryTemp, aes(x = tempCat, y = meanN) ) +

geom_point(size = 1) +

geom_errorbar(aes( ymin = meanN - SE, ymax = meanN + SE), width = 0.5, size = 0.25 ) +

theme_bw() +

theme( panel.border = element_blank(),

panel.grid.major = element_blank(),

panel.grid.minor = element_blank(),

axis.line = element_line(colour = 'black', size = 0.25),

axis.ticks = element_line(colour = "black", size = 0.25),

axis.ticks.length=unit(-0.25, "cm"),

axis.text.x = element_text(margin=unit(c(0.5,0.5,0.5,0.5), "cm"), size = 10),

axis.text.y = element_text(margin=unit(c(0.5,0.5,0.5,0.5), "cm"), size = 10),

legend.position="none",

axis.title.x=element_text( size = 12 ),

axis.title.y=element_text( size = 12 ) ) +

labs( x = "Mean Temperature (\u00B0C)", y = "Yield (T/ha)") +

theme(axis.text.x = element_text(angle = 90))

fig4d

multiplot(fig4a + labs( tag = "A"), fig4b+ labs( tag = "B"), fig4c+ labs( tag = "C"), fig4d+ labs( tag = "D"), cols = 2)

# Linear Model for Diversity and plots

MST_RC_ASD_IMP_WD<-read.csv("MST_RC_ASD_IMP_WD.CSV")

stderr <- function(x) sd(x) / sqrt(length(x))

MST_RC_ASD_IMP_WD$DIV <- as.factor(MST_RC_ASD_IMP_WD$DIV)#To change DIV to 4 level factor

#Divide treatment by control to make weed density % difference

MST_RC_ASD_IMP_WD$WDDif<- (MST_RC_ASD_IMP_WD$Treat_Mean/MST_RC_ASD_IMP_WD$Control_Mean)*100

#Look at diversity and change in weed density

LM1 <- lm( WDDif ~ DIV, data=MST_RC_ASD_IMP_WD)

anova(LM1)

summary(LM1)

coeffs <- data.frame( summary(LM1)$coefficients )

coeffs$names <- str_remove( rownames(coeffs), "MST_RC_ASD_IMP_WD" )

RCD<- c("1", "2", "3","4")#For the x tick labels

fig5a <- ggplot(coeffs, aes(x = names,Estimate, y = Estimate) ) +

geom_point(size = 1) +

geom_errorbar(aes( ymin = Estimate - Std..Error, ymax =Estimate + Std..Error ), width = 0.2, size = 0.25 ) +

theme_bw() + scale_x_discrete(labels= RCD)+

theme( panel.border = element_blank(),

panel.grid.major = element_blank(),

panel.grid.minor = element_blank(),

axis.line = element_line(colour = 'black', size = 0.25),

axis.ticks = element_line(colour = "black", size = 0.25),

axis.ticks.length=unit(-0.25, "cm"),

axis.text.x = element_text(margin=unit(c(0.5,0.5,0.5,0.5), "cm"), size = 10),

axis.text.y = element_text(margin=unit(c(0.5,0.5,0.5,0.5), "cm"), size = 8),

legend.position="none",

axis.title.x=element_text( size = 12 ),

axis.title.y=element_text( size = 12 ) ) +

labs( x = "Rotation Crop Diversity", y = "Density Change Coefficient") +

theme(axis.text.x = element_text(angle = 0, vjust = .7, hjust=.65))

fig5a

fig5b<-ggplot(data = MST_RC_ASD_IMP_WD, aes(x=DIV, y=WDDif)) +

geom_boxplot(fill=c('red', 'Yellow', 'blue','green'))+

labs( x = "Rotation Crop Diversity", y = "Weed Density Change")

fig5b

#Redo the LMER with diversity as a factor using effect size

mixed.mod1 <- lmer(HEDGES ~

DIV +

(1|Study_ID) ,

data=MST_RC_ASD_IMP_WD,

weights = 1/VAR_G,

na.action = "na.omit")

anova(mixed.mod1)

summary(mixed.mod1)

coeffs <- data.frame( summary(mixed.mod1)$coefficients )

coeffs$names <- str_remove( rownames(coeffs), "MST_RC_ASD_IMP_WD" )

fig5c <- ggplot(coeffs, aes(x = names,Estimate, y = Estimate) ) +

geom_point(size = 1) +

geom_errorbar(aes( ymin = Estimate - Std..Error, ymax =Estimate + Std..Error ), width = 0.2, size = 0.25 ) +

theme_bw() + scale_x_discrete(labels= RCD)+

theme( panel.border = element_blank(),

panel.grid.major = element_blank(),

panel.grid.minor = element_blank(),

axis.line = element_line(colour = 'black', size = 0.25),

axis.ticks = element_line(colour = "black", size = 0.25),

axis.ticks.length=unit(-0.25, "cm"),

axis.text.x = element_text(margin=unit(c(0.5,0.5,0.5,0.5), "cm"), size = 10),

axis.text.y = element_text(margin=unit(c(0.5,0.5,0.5,0.5), "cm"), size = 8),

legend.position="none",

axis.title.x=element_text( size = 12 ),

axis.title.y=element_text( size = 12 ) ) +

labs( x = "Rotation Crop Diversity", y = "Effect Size (g)") +

theme(axis.text.x = element_text(angle = 0, vjust = .7, hjust=.65))

fig5c

fig5d<-ggplot(data = MST_RC_ASD_IMP_WD, aes(x=DIV, y=HEDGES)) +

geom_boxplot(fill=c('grey', 'grey', 'grey','grey'))+

labs( x = "Rotation Crop Diversity", y = "Effect Size (g)")

fig5d

Figure5e <- ggplot( MST_RC_ASD_IMP_WD, aes(x = DIV, y = HEDGES) ) +

geom_point( size = 1) +

geom_errorbar( aes(ymin = HEDGES - VAR_G, ymax = HEDGES + VAR_G, width = 0.1, )) +

theme_bw() +

theme( panel.border = element_blank(),

panel.grid.major = element_blank(),

panel.grid.minor = element_blank(),

legend.position="none",

axis.line = element_line(colour = 'black', size = 0.25),

axis.ticks = element_line(colour = "black", size = 0.25),

axis.text.x = element_text(size = 10),

axis.text.y = element_text(size = 8),

axis.title.x=element_text(size = 14),

axis.title.y=element_text(size = 14) ) +

geom_hline(yintercept = 0, linetype = "dashed") +

labs(x = "Rotation Crop Diversity") + labs( y = "Effect Size (g)", las=2)

Figure5e

multiplot(fig5a + labs( tag = "A"), fig5c+ labs( tag = "B"), cols = 1)
